# Bio‐Inspired Electrodes with Rational Spatiotemporal Management for Lithium‐Ion Batteries

**DOI:** 10.1002/advs.202400405

**Published:** 2024-04-29

**Authors:** Zelai Song, Weifeng Li, Zhenhai Gao, Yupeng Chen, Deping Wang, Siyan Chen

**Affiliations:** ^1^ College of Automotive Engineering Jilin University Changchun 130022 China; ^2^ National Key Laboratory of Automotive Chassis Integration and Bionic Jilin University Changchun 130022 China; ^3^ CAS Key Laboratory for Biomedical Effects of Nanomaterials and Nanosafety National Center for Nanoscience and Technology Beijing 100190 China; ^4^ General Research and Development Institute China FAW Corporation Limited Changchun 130013 China

**Keywords:** bio‐inspired electrode, bionics, electrochemical performance, lithium‐ion battery, thermal safety

## Abstract

Lithium‐ion batteries (LIBs) are currently the predominant energy storage power source. However, the urgent issues of enhancing electrochemical performance, prolonging lifetime, preventing thermal runaway‐caused fires, and intelligent application are obstacles to their applications. Herein, bio‐inspired electrodes owning spatiotemporal management of self‐healing, fast ion transport, fire‐extinguishing, thermoresponsive switching, recycling, and flexibility are overviewed comprehensively, showing great promising potentials in practical application due to the significantly enhanced durability and thermal safety of LIBs. Taking advantage of the self‐healing core–shell structures, binders, capsules, or liquid metal alloys, these electrodes can maintain the mechanical integrity during the lithiation–delithiation cycling. After the incorporation of fire‐extinguishing binders, current collectors, or capsules, flame retardants can be released spatiotemporally during thermal runaway to ensure safety. Thermoresponsive switching electrodes are also constructed though adding thermally responsive components, which can rapidly switch LIB off under abnormal conditions and resume their functions quickly when normal operating conditions return. Finally, the challenges of bio‐inspired electrode designs are presented to optimize the spatiotemporal management of LIBs. It is anticipated that the proposed electrodes with spatiotemporal management will not only promote industrial application, but also strengthen the fundamental research of bionics in energy storage.

## Graphical abstract

This review illustrates that the bio‐inspired electrodes own rational spatiotemporal management in terms of self‐healing, fast ion transport, flexibility, recycling, self‐extinguishing, and thermoresponsive switching. Structures and materials in nature have evolved into the most efficient forms and adapted to various environmental conditions over 3.5 billion years. Taking advantage of the smart multi‐functions from natural structures and materials, the bio‐inspired electrodes present to optimize the spatiotemporal management of LIBs. It is anticipated that the proposed electrodes with spatiotemporal management will not only promote industrial application, but also strengthen the fundamental research of bionics in energy storage.

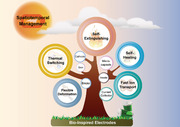

## 1 Introduction

Employing high‐energy‐density lithium‐ion batteries (LIBs) is an effective approach to improve human life, cope with climate change, promote green development and achieve the strategic goal of carbon peak carbon neutrality.^[^ ^1^, ^2^ ^]^ LIBs have been the state‐of‐the‐art energy storage technology due to their excellent cycling stability, high energy density, high power density, and technology integration.^[^ ^3^, ^4^, ^5^, ^6^, ^7^ ^]^ They are highly desirable for electric vehicles, portable electronics, renewable energy utilization and carbon negative society.^[^ ^8^ ^]^ However, the critical challenge is the simultaneous improvement of durability and fast charging capabilities, which is related to the energy density and power of LIBs.^[^ ^9^, ^10^, ^11^, ^12^, ^13^, ^14^ ^]^ Furthermore, mechanical, electrical, or thermal failure causes a temperature rise to the critical levels of fires, and thus the thermal runaway of LIB has seriously hindered large‐scale development.^[^ ^15^, ^16^, ^17^, ^18^, ^19^ ^]^ Nowadays, conventional safety measures to prevent thermal runaway are either internal, which stops the heat‐generating processes inside LIBs, or external, which controls the exterior temperature of LIBs and electrically isolates LIBs.^[^ ^20^ ^]^ It is an effective way to achieve task‐specific functions along with deepening understanding of the underlying mechanisms of various components in electrodes. Simultaneously, the constructions of electrodes aim to improve the structural stability, reliability, durability, and thermal safety of LIBs and to curb greenhouse gas emissions.

From the perspective of either engineering or chemistry, it is undoubtedly intelligent to learn from nature to remove the barriers to development.^[^ ^21^, ^22^, ^23^ ^]^ Taking inspiration from natural storage systems and energy metabolism, diverse approaches have been developed to effectively and rationally improve active electrode materials' electrochemical performance and manufacture advanced LIBs.^[^ ^24^ ^]^ Complex degradation in LIB involves various processes interconnected with materials' thermodynamic, chemical, and mechanical instability of materials.^[^ ^25^, ^26^ ^]^ From the above, different self‐healing approaches based on bio‐inspired concepts can be employed due to the diversity of degradation processes. LIBs comprise the cathode, anode, separator, current collector, electrolyte, and battery housing.^[^ ^27^, ^28^ ^]^ Their degradation can be minimized in smart electrodes via preventive steps, like artificial interfaces,^[^ ^29^ ^]^ coatings,^[^ ^30^, ^31^, ^32^, ^33^, ^34^, ^35^ ^]^ self‐healing binders,^[^ ^36^, ^37^, ^38^, ^39^, ^40^ ^]^ liquid metal alloys,^[^ ^41^, ^42^, ^43^, ^44^, ^45^, ^46^, ^47^ ^]^ microcapsules,^[^ ^48^, ^49^, ^50^ ^]^ and current collectors.^[^ ^51^ ^]^ For instance, silicon (Si) anode severe volume expansion of up to 300% in the lithiation state leads to solid electrolyte interface (SEI) rupture, which decreases the electrochemical performance of LIB during cycling processes, and its lifetime become shortened.^[^ ^52^, ^53^, ^54^, ^55^, ^56^ ^]^ Fortunately, self‐healing strategies provide new avenues to resolve the above issues via repairing the impaired parts to improve the electrochemical performance.^[^ ^57^, ^58^, ^59^ ^]^

On the other hand, bio‐inspired porous structure onto ultrathick electrode achieves high areal capacity and excellent rate capability.^[^ ^60^, ^61^, ^62^, ^63^ ^]^ Biomass materials own the potential application for LIBs due to their extraordinary properties, sustainability, and low cost.^[^ ^64^, ^65^ ^]^ High electrochemical performance has been achieved in biomass carbon materials with optimized porous structures that shorten the diffusion path of ions.^[^ ^66^, ^67^ ^]^ These electrodes can achieve some other special functions based on bio‐inspired structures. Many emerging bio‐inspired designs achieve high storage capacity and mechanically flexible LIBs (FLIBs) are successfully developed.^[^ ^68^, ^69^, ^70^, ^71^, ^72^, ^73^ ^]^ Furthermore, the microstructures on geckos feet have amazing controllable attachment and detachment capabilities,^[^ ^74^, ^75^ ^]^ which provide the inspiration for preparing the structured composite film with similar adhesion properties to achieve the easy‐to‐recycle of electrodes.^[^ ^76^ ^]^ Thus, it is expected that bio‐inspired electrodes will contribute significantly to the innovations of upcoming LIBs.

Equally as importantly, thermal runaway risks faced by LIBs are classified into the specific categories of thermal stability, fire hazard, rate hazard, pressure hazard, and heat hazard.^[^ ^77^ ^]^ The conventional flame‐retardant method is to modify the components of LIB to improve thermal safety.^[^ ^78^ ^]^ For example, adding flame retardants into electrolytes has been an effective way to enhance LIB safety,^[^ ^79^ ^]^ but it often decays electrochemical performance.^[^ ^18^ ^]^ Thus, the other components of the electrode materials, such as binders,^[^ ^10^, ^80^ ^]^ microcapsules,^[^ ^81^, ^82^, ^83^ ^]^ and current collectors,^[^ ^84^, ^85^, ^86^, ^87^ ^]^ can add flame retardants. Some emerging materials can change significantly in a controllable manner when subjected to various stimuli such as thermal, electrical, environmental, mechanical, and magnetic influences.^[^ ^88^ ^]^ Subsequently, these modified properties can promptly revert to their initial states once the external stimulus disappears.^[^ ^89^ ^]^ Ensuring thermal safety during operation is the major problem in the development of the electrodes for LIBs. Developing thermoresponsive switching electrodes is a considerable strategy to enhance their thermal safety.^[^ ^90^ ^]^ Thus, mixing electrode or coating current collector with positive temperature coefficient (PTC) materials can rapidly switch LIB off when it overheats, and quickly regain functionality normal operating conditions are restored.^[^ ^91^, ^92^, ^93^, ^94^ ^]^

**Figure 1:**
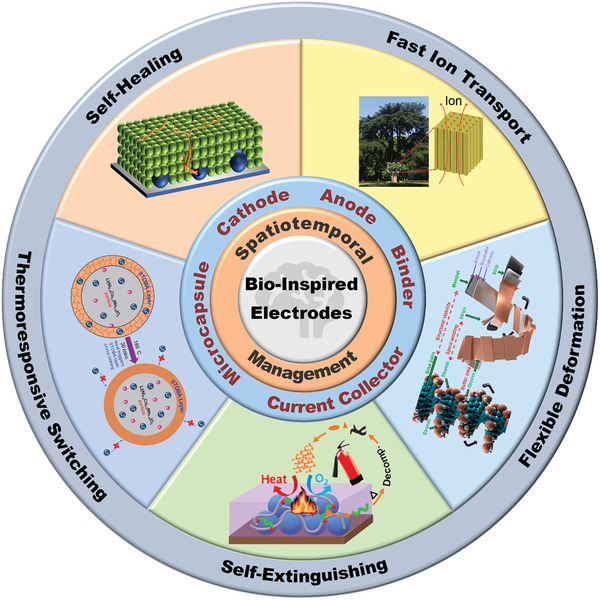
Schematic summary of bio‐inspired electrodes possessing spatiotemporal management on LIBs in this review. Reproduced with permission.^[^ ^48^ ^]^ Copyright 2022, ACS Applied Energy Materials. Reproduced with permission.^[^ ^62^ ^]^ Copyright 2018, Advanced Materials. Reproduced with permission.^[^ ^72^ ^]^ Copyright 2022, Nano Letters. Reproduced with permission.^[^ ^10^ ^]^ Copyright 2020, ACS Applied Materials & Interfaces. Reproduced with permission.^[^ ^94^ ^]^ Copyright 2014, RSC Advances.

The commercial application of LIBs has led to further breakthroughs in electric vehicles and other energy storage devices. However, electrochemical performance and thermal safety remain crucial issues and obstacles to their widespread application.^[^ ^95^ ^]^ Facing these challenges, many efforts have focused on enhancing the electrochemical performance, thermal safety, and specific capacity of current materials or developing new ones for bio‐inspired electrodes. Herein, the review innovatively summarizes the pioneering and representative progress in developing nature‐inspired LIBs‐related materials and structures. **Figure** **1** illustrates that the electrodes possess spatiotemporal management in terms of self‐healing, fast ion transport, flexibility, recycling, self‐extinguishing, and thermoresponsive switching, which have the best application prospects in energy storage devices. Structures and materials in nature have evolved into highly efficient forms and have adapted to diverse environmental conditions over 3.5 billion years. With the deepened understanding of the relationship between structures and functions of the bio‐inspired electrodes, it can be envisioned that they can be widespread application in the near future.

## 2 Challenges of Electrodes

LIB is the primary kind of power source for electric vehicles and energy storage devices.^[^ ^3^ ^]^ Extremely fast charging, with a target of a 15 min recharge time, has the potential to accelerate the mass market adoption of electric vehicles, lessen the greenhouse effect, and ultimately increase energy security for countries.^[^ ^11^ ^]^ LIBs continue to age and decay, resulting in continual changes in their behavioral characteristics and parameters.^[^ ^12^ ^]^ The influencing factors of LIB health status include cycling lifetime, temperature, humidity, dust, and application scenarios. Furthermore, thermal safety is one of nonnegligible issues as well. The following sections discuss current challenges of the electrodes in detail.

### 2.1 Electrochemical Performance

**Figure 2:**
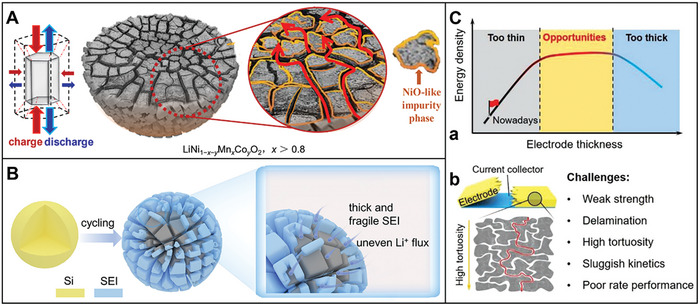
Capacity fading schematic illustration of the electrodes: A) Cathode.^[^ ^97^ ^]^ Copyright 2018, Chemistry of Materials. B) Anode.^[^ ^56^ ^]^ Copyright 2023, Journal of Electroanalytical Chemistry. C) Thick‐film electrode. a) Opportunities and b) challenges accompanying the thick‐film electrode design.^[^ ^98^ ^]^ Copyright 2019, Advanced Energy Materials.

The lifetime of LIBs is limited and requires enhancement to meet the long‐term usage demands of electric vehicles, which are expected to remain in service for 20 years or more.^[^ ^35^, ^96^ ^]^ However, the degradation processes in LIB are the crucial issues impeding their long lifetimes. The major degradation processes include mechanical degradation, and chemical and electrochemical degradation.^[^ ^25^ ^]^ Therein, mechanical degradation includes particle and electrode surface cracking and thus loss of electrical connectivity. Moreover, chemical and electrochemical degradations are the processes in terms of solid electrolyte interface (SEI) growth and decomposition, gas evolution, dissolution of transition metals, current collector corrosion, and dendrite formation, among other factors.^[^ ^25^ ^]^ Thus, electron/ion transport, electrode charge transfer, and temperature are critical obstacles to fast charging. **Figure** **2** illustrates that the challenges of enhancing electrochemical performance are caused by the cathode materials,^[^ ^97^ ^]^ anode materials,^[^ ^56^ ^]^ and thick‐film electrodes.^[^ ^98^ ^]^ Capacity fading progresses of electrodes will be discussed in the following sections.

#### 2.1.1 Cathode Degradation

Cathode materials are prone to bulk and interfacial degradation issues, which adversely affect their electrochemical performance.^[^ ^30^, ^99^ ^]^ For instance, LiNi_1−_ *_x_*_−_ *_y_* Mn*_x_* Co*_y_* O_2_ (NMC) will deteriorate the electrochemical performance and structural stability during charge–discharge cycling.^[^ ^100^, ^101^ ^]^ And many commercial cathode materials (for example, LiFePO_4_, LiCoO_2_, etc.) for lithium‐ion batteries face the same issues. The degradation processes of Ni‐rich NMC includes different types of capacity loss, such as initial capacity loss, sudden capacity loss and gradual capacity loss, all accompanied by an increase in impedance.^[^ ^102^ ^]^ Therein, the relative amounts of Ni, Mn and Co can be adjusted (typically increases in Ni) to make for augmented performances and different negative features. As a result, the relatively high content of Ni^4+^ in the charged state shortens the lifetime of LIB owing to inevitable capacity and voltage degradation during cycling.^[^ ^103^ ^]^

On the other hand, the performance degradation of LiNi_0.6_Mn_0.2_Co_0.2_O_2_ (NMC 622) is related to its structural instability. As a results, oxygen releases and byproduct forms around the cathode surface at a high charging voltage and temperature.^[^ ^104^ ^]^ The degradation process of Ni‐rich NMC‐based cathodes originates from the lithiation/delithiation process during charge/discharge cycling.^[^ ^105^ ^]^ The anisotropic lattice strains generate intense stresses at primary particle boundaries during the lithiation–delithiation cycling. The expansion and contraction of primary particles (crystal lattices) gradually generate tension. Cathode suffers structural degradation, the formation and propagation of microcracks and then secondary particle crushing (Figure 2A).^[^ ^97^, ^101^, ^106^ ^]^ Besides, higher Ni content triggers the reconstruction of cathode surface structure during long‐term cycling, which accompanies the dissolution of metal ions. This progress accelerates the collapse of the crystal structure. Furthermore, the degradation process will be speeded up by the large release of lattice oxygen from the host structure, which simultaneously can bring about a thermal safety crisis.

Further, electrolyte infiltration and oxygen release can exacerbate the abovementioned issues. The corrosion caused by electrolyte leads to the formation of an irreversible NiO‐like impurity layer at the surfaces along the primary particle boundaries, known as the rock‐salt phase. This layer significantly increases interface impedance, consequently producing capacity decay. And the inhomogeneity of electrochemical reactions will become more serious when the cathode particles become more aspherical.^[^ ^107^ ^]^ Thus, construction of the surface protective layer and suppression of the anisotropic volume changes are crucial to overcome capacity decaying. Improving electrodes needs a delicate control of active materials and electrode formulation.^[^ ^105^ ^]^ The modification and design strategies of the ion pathways include coating, element doping, new functional structures, and composite pathways.^[^ ^6^ ^]^ Therefore, more valuable strategies need to be proposed to solve these issues.

#### 2.1.2 Anode Degradation

Anode is a carrier of electrons and lithium‐ions during charge–discharge cycling, and plays a role of the energy storage and release. The potential candidates for anode materials include hard carbon,^[^ ^108^, ^109^ ^]^ Si‐based anodes,^[^ ^110^, ^111^, ^112^, ^113^, ^114^ ^]^ graphite,^[^ ^115^, ^116^, ^117^ ^]^ Sn‐based anodes,^[^ ^118^, ^119^, ^120^ ^]^ Ga‐based anode,^[^ ^121^, ^122^ ^]^ and TiO_2_,^[^ ^123^ ^]^ etc. They usually possess wide availability, sustainability, high capacity, and stable physicochemical properties. But different anode materials possess various defects, such as low Li diffusivity, poor initial Coulombic efficiency, low electron conductivity, poor cycling performance, unsatisfactory reversible capacities, and severe safety concerns.^[^ ^124^, ^125^, ^126^ ^]^ Therein, silicon (Si) is a promising material for high‐capacity anodes due to its natural abundance, low working potential, and high theoretical capacity (4200 mAh g^−1^) compared to the state‐of‐the‐art carbonaceous counterpart.^[^ ^10^, ^54^, ^127^, ^128^, ^129^, ^130^, ^131^ ^]^ Since each anode material structure has its strengths and weaknesses. There are two main drawbacks that impede its further practical development and applications.^[^ ^132^ ^]^

The primary challenge associated with Si anodes is the structural degradation and instability of the SEI owing to the substantial volume change (≈300%) during lithiation (Figure 2B).^[^ ^52^, ^53^, ^56^ ^]^ This can lead to electrode delamination from the current collector.^[^ ^133^ ^]^ Consequently, side reactions with the electrolyte occur, causing severe structural pulverization and rapid capacity fading of the electrode.^[^ ^38^, ^134^, ^135^, ^136^, ^137^, ^138^, ^139^, ^140^, ^141^ ^]^ Therein, the Si anode forms SEI and achieves prelithiation during the first cycle, resulting in excess lithium‐ion consumption and large irreversible capacity losses.^[^ ^142^, ^143^, ^144^ ^]^ The other challenge is the relatively low intrinsic electronic conductivity, which is severely affecting the rate capability.^[^ ^132^, ^145^, ^146^ ^]^ The instability of SEI seriously causes electrical contact failure inside the anode and thus the rapid decline of cycling lifetime.^[^ ^56^, ^147^, ^148^ ^]^ Rational design of the anode is an effective means to cope with volume expansion, poor conductivity, and capacity decay due to SEI rupture.^[^ ^52^, ^149^ ^]^ Thus, mitigating Si particle fracture, unstable SEI, and electrode cracking failure remain primary challenges.

In addition, silicon oxide (SiO*_x_*) is a promising and potential anode material for LIBs, owing to its high capacity, low cost, abundance, and safety.^[^ ^150^, ^151^, ^152^, ^153^, ^154^, ^155^ ^]^ But conventional binder polyvinylidene difluoride (PVDF) can hardly keep the integrity of the SiO*_x_* and Si particles because of its weak van der Waals force.^[^ ^156^, ^157^ ^]^ The challenges of poor cyclability, unsatisfactory electrical conductivity, severe capacity decay, sluggish charge transfer, and low initial Coulombic efficiency require urgent resolution.^[^ ^158^ ^]^ Therefore, greater attention must be directed toward understanding the internal relationship between electrochemical performance and structural characteristics. Si‐based anodes with high areal capacities are frequently made of porous materials to mitigate the significant volume shift that occurs during cycling, which compromises their volumetric capacities.^[^ ^110^ ^]^ Furthermore, composite carbon materials as anodes can enhance the capacity, cycling lifetime, and safety of LIB. Nano‐structured carbon materials such as fullerene, carbon nanotube and graphene possess excellent electrochemical performance.^[^ ^144^, ^159^, ^160^ ^]^ Further, the optimized combination of Si and high‐performance graphite will create synergistic effects arising from the high theoretical capacity of Si and the ultra‐high stability of graphite. This approach will expand the practical applications of Si‐based anode materials.^[^ ^52^, ^161^ ^]^ Designing new highly practical Si‐based anodes with low‐volume expansion, high initial Coulombic efficiency, low volume fluctuations, high cyclability, and high electrical conductivity are avenues for developing smart anodes.^[^ ^162^ ^]^

The other emerging anode materials for the next‐generation advanced LIBs include Sn‐based,^[^ ^119^ ^]^ and Ga‐based anodes.^[^ ^121^ ^]^ Ga‐based liquid metals (LMs) are potential anode materials for LIBs owing to their self‐healing capability, nontoxic nature, and high theoretical capacity. However, Ga‐based alloys experience significant volume changes, leading to poor cycling lifetime owing to the lithium storage alloying/dealloying reaction mechanism. Additionally, the poor wettability of LMs on various substrates (such as carbon materials, stainless steel, etc.) and their tendency to form alloys with many metallic current collectors (Cu and Al foil) make them challenging to incorporate into electrodes with superior electrochemical performance.^[^ ^163^ ^]^ Thus, rationally developing self‐healing LM anodes is one of the best candidate materials for smart LIBs when these issues are resolved.

#### 2.1.3 Ultrathick Electrodes

As society advances, there has been a growing push in the industry to enhance the capacity of electrode materials, thereby increasing the energy density of LIBs while simultaneously lowering costs.^[^ ^3^, ^164^, ^165^ ^]^ Ultrathick electrodes enable to reduce the fraction of inactive battery components like separators and current collectors.^[^ ^166^, ^167^, ^168^, ^169^ ^]^ As a result, ultrathick electrode design is an effective strategy to improve the specific energy of LIBs without changing the underlying material chemistry (Figure 2C‐a).^[^ ^170^ ^]^ But after LIB assembly from thick‐film electrodes, some issues occur in electrochemical reaction processes during operation. With mass‐specific capacity enhancement, cathode enable to achieve this at the expense of rate capability and structural stability.^[^ ^102^ ^]^ The low lithium‐ion conductivity and electrolyte infiltration difficulty are prompted by the ultralong electron and ion transport pathways in conventional random micro‐structured electrodes (Figure 2C).^[^ ^62^, ^63^, ^171^, ^172^ ^]^ It heavily degrades the rate capability of the ultrathick electrodes and their large unusable capacity prompted by high internal resistances.^[^ ^13^ ^]^ With the thickness of composite electrodes increasing from ≈80 – 100 µm to several hundred µm, the ultrathick electrodes will heighten the energy density, but at the expense of the power density.^[^ ^173^ ^]^ The heterogeneous ion and electron transport prompts a larger state‐of‐charge variation in thick‐film electrodes, a more nonuniform current distribution, and thus decreased cycling stability.^[^ ^174^, ^175^, ^176^ ^]^

Further, the higher mass loading will prompt more severe mechanical strain and stress accumulation in thick‐film electrodes, which leads to more cracks and exfoliations in the coating electrode layers during cycling process (Figure 2C‐b).^[^ ^98^, ^177^, ^178^ ^]^ The higher areal loading electrode leads to reduce the quantity of the SEI formed.^[^ ^144^ ^]^ The thick‐film electrodes sustain drastic volume changes and lower mechanical strength. Their electrochemical performance will be faster degraded by more severe pulverization of active materials.^[^ ^179^ ^]^ Herein, enhancing the electrochemical performance of high‐energy LIBs is and will be an important issue to be solved urgently in the future. Both the optimization of LIB structures and the selection of electrode materials enable to improve electrochemical performance and inhibit thermal runaway in LIBs.^[^ ^180^ ^]^ Material improvement strategies include material modification, novel functional additives, and material development.^[^ ^95^ ^]^ The successful development of bio‐inspired electrodes will accelerate the application of bio‐inspired technology to further enhance the performance of LIBs, namely, energy density, cycling stability, lifetime, and safety.^[^ ^66^ ^]^ Thus, ultrathick electrodes with fast ion transport characteristics based on bio‐inspired structures have the best application prospects in the future.

### 2.2 Flexible and Recycle Functions

With the development of the times, the exploitation of flexible LIBs (FLIBs) is critical for the next generation of electronics.^[^ ^68^ ^]^ And high‐performance stretchable FLIBs are essential components for flexible devices as well.^[^ ^181^ ^]^ However, they may be broken under the twisting and bending deformations, causing the work failure or even acute safety issues.^[^ ^69^ ^]^ In this context, the fast development of flexible and wearable electronics proposes persistent requirements for high‐performance FLIBs. The different components under mechanical deformation are prone to cause delamination. Even though all separators, electrodes, and current collectors can achieve flexibility, the outer packages need to possess the same flexible level as well, requiring coping with the mechanical strength of inner metals to release strain in metal layers.^[^ ^182^ ^]^ Much progress has been achieved recently, but it is challenging to obtain remarkable stretchability and flexibility, high power density, and high energy density simultaneously.^[^ ^71^, ^182^ ^]^ As flexible devices evolve, there is a growing demand to engineer high‐performance FLIBs that offer superior flexibility, durability, and safety. It is essential to ensure these batteries can deform along with the device while maitaining their retaining electrochemical performance. Most importantly, the artificial synthesis of nanomaterials with superior mechanical properties to achieve excellent flexible functions is time‐consuming and expensive. The application of bio‐inspired structures and materials is the efficient solution to address the above limitations. Unlike conventional rechargeable LIBs, FLIBs are essential for bendable and biocompatible characteristics. Thus, bio‐inspired electrodes can achieve multiple functions owing to the bio‐inspired structures in nature.

Recycling end‐of‐life electronic devices containing expensive metals, such as LIBs, is a critical challenge for both cost reduction and environmental reasons.^[^ ^183^ ^]^ The estimated two million metric tons of annual garbage from LIB worldwide,^[^ ^184^ ^]^ and thus the rapidly growing LIB mass market demands both high energy density and waste‐management solutions.^[^ ^185^ ^]^ The anode accounts for ≈5% – 15% in LIB cost, and it is one of the most significant raw materials for LIBs.^[^ ^186^ ^]^ Our society promises to gain significantly from the recycling of previously utilized LIBs in terms of economic growth, environmental protection, and raw material savings. The separation process of a current collector from the composite film of electrode is a typical, crucial problem for LIB recycling even if many process chains have been used or are being developed to recycle LIBs.^[^ ^76^, ^187^ ^]^ Fortunately, the advantages of materials and structures in nature create through a bio‐inspired process far more than make compensate for the drawbacks of conventional materials and structures.^[^ ^21^ ^]^ Thus, bio‐inspired electrodes possessing easy‐to‐recycle function and flexible deformation can be developed based on bio‐inspired structures for future usage.

### 2.3 Thermal Safety

In LIB developments, the significant enhancement of performance may cause some potential safety hazards. High safety and energy density have become the important goals for the exploitation of state‐of‐the‐art LIBs.^[^ ^87^, ^188^ ^]^ However, thermal safety is the most prominent and concerning problem for the widespread application of LIBs. Currently, thermal runaway has become the most concerning safety hazard, posing a serious concern for large‐scale energy storage applications.^[^ ^19^ ^]^ Several product recalls and high‐profile mishaps over the past ten years have been caused by thermal runaway and the ensuing flame and explosion.^[^ ^20^ ^]^ Thermal runaways result from the intrinsic qualities of LIBs being destroyed by abuse and improper use. In practical applications, three common abuse conditions can lead to cause the failure of LIBs: mechanical damage (such as crushing, collision, and nail penetration), thermal abuse (resulting from overheating), and electrical abuse (including overcharge, overdischarge, and short‐circuits).^[^ ^88^, ^189^ ^]^ If the heats caused by abuse conditions can hardly dissipate, the temperatures of LIBs will arise further, and hence accelerating the process of heat release.

**Figure 3:**
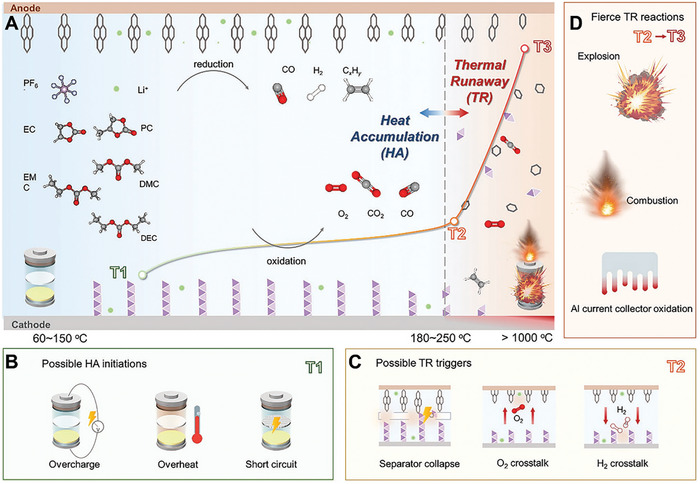
Schematic illustration of the two‐stage development of thermal failure: A) The thermal failure process in LIBs; B) Improper operations that initiate the heat accumulation stage (causes of T1); C) Possible events that trigger the thermal runaway (causes of T2); D) Fierce T3 reactions.^[^ ^9^ ^]^ Copyright 2023, Advanced Energy Materials.

Further, thermal failure has two stages: mild heat accumulation and intense thermal runaway (**Figure** **3**A).^[^ ^9^, ^93^ ^]^ The three characteristic temperatures of LIBs separate these stages: 1) the onset temperature of battery self‐heating (T1); 2) the triggering temperature of the intense thermal runaway (T2); and 3) the maximum temperature reached during thermal runaway (T3).^[^ ^9^ ^]^ As the temperature increases, several chemical transformations occur in the LIB, including electrolyte decomposition, reactions between the electrolyte/cathode and anode materials, SEI layer decomposition, and reactions between the binder and electrodes.^[^ ^15^ ^]^ The decomposition of the SEI exposes the anode surface to the electrolyte, initiating a series of chemical reactions between the anode and electrolyte.^[^ ^186^ ^]^ These processes raise the internal temperature of the LIB, ultimately leading to thermal runaway.

The heat accumulation stage initiates at T1 (60 – 150 °C), triggered by internal exothermic reactions resulting from improper operating conditions, such as overheating, overcharging, external or internal short circuits, etc. (Figure 3B).^[^ ^9^ ^]^ Internal short circuiting is a complex phenomenon involving multiple processes, including electrochemistry, thermodynamics, and heat transfer.^[^ ^190^ ^]^ It is a common thermal runaway characteristic and can arise from mechanical, thermal, or electrical abuse.^[^ ^191^, ^192^ ^]^ Typically, if the separator fails to adequately isolate the cathode and anode, it can lead to a short circuit in the LIB, potentially resulting in an explosion or combustion (Figure 3C,D).^[^ ^78^ ^]^ The materials' properties and the SEI play a significant role in determining the critical temperature for thermal runaway.^[^ ^193^ ^]^ Generally, the heat sources in LIBs are categorized as reversible heat, primarily generated by electrochemical reactions, and irreversible heat, which is largely attributed to the Joule effect.^[^ ^194^ ^]^ Under extreme conditions, LIBs are inevitably prone to failure, resulting in the release of significant heat. Excessive heat generation can lead to adverse effects such as capacity loss, electrical imbalance, and ultimately, thermal runaway,^[^ ^180^ ^]^ severely limiting restricts the application potential of LIBs.

Consequently, the significant heat generated by electrical abuse is a primary safety concern for LIBs. However, this issue can be mitigated by preventing overcharge, overdischarge, overheating, mechanical damage, etc.^[^ ^88^ ^]^ As depicted in Figure 3A, maintaining temperatures below 60 °C is optimal for ensuring LIB safety, effectively preventing the onset of thermal runaway from abusive conditions.^[^ ^77^ ^]^ To address these challenges, conventional active methods, such as fans and refrigerants, alongside passive techniques like heat sinks or heat shields, are employed to cool LIBs.^[^ ^20^ ^]^ Combining these approaches ensures thermal safety by adapting to changing external conditions. However, traditional strategies for averting thermal runaway cannot adjust charge–discharge rates based on temperature fluctuations or restore original performance after cooling to room temperature.^[^ ^195^ ^]^ Thus, modifying the internal components of LIBs is an alternative appoach to enhancing thermal safety. Optimization of LIB structure and careful selection of electrode materials can effectively improve and mitigate the kind of thermal runaway.^[^ ^180^ ^]^ Developing thermal‐responding and flame‐retardant materials for bio‐inspired electrodes is also promising in addressing thermal runaway and enhancing LIB safety.^[^ ^196^ ^]^

Fortunately, many great efforts have been made to create reliable batteries through fire safety characterization, thermal management, and advanced materials design.^[^ ^197^ ^]^ The emerging bio‐inspired electrodes containing flame‐retardant materials possess intrinsic nonflammability and fire‐extinguishing properties, which enable to ensure LIB safety radically by suppressing fire hazards.^[^ ^198^ ^]^ The electrodes containing thermal‐responding materials can switch LIB off via insulating the electron/ion transport at risky temperatures, preventing LIB overheating, eradicating possible thermal runaway, and retaining LIB at a safe state. Consequently, conceptual thermoresponsive switching electrodes have been proposed to enhance the thermal safety of large‐scale LIBs applications. Since it will result from the abnormal temperature of LIB increasing when some side reactions occur.^[^ ^19^ ^]^ The bio‐inspired electrodes contain a positive temperature coefficient (PTC) compound as the conductive material, which increases its resistivity at temperatures above the PTC melting point. The electrodes containing PTC materials increase resistance, and in turn, LIB reactions will be switched off.^[^ ^90^ ^]^ Thermoresponsive switching electrodes enable to provide a safety control for LIBs under a wide range of applications.^[^ ^199^ ^]^ Due to the lack of electrochemically compatible materials owning suitable thermal‐responding capabilities, such an electrode is difficult to be developed. Furthermore, shape memory polymer is a bio‐inspired thermal‐responding material as well. Herein, an emerging shape‐memorized current collector can successfully brake thermal runaway at LIB internal overheating status.^[^ ^200^ ^]^ The smart current collector is a new avenue to achieve fire‐extinguishing LIBs. Meanwhile solving the problem of thermal runaway, the electrochemical performance should be strengthened with attention.

### 2.4 Bio‐Inspired Electrode Design

From the above, the optimization and design of electrode architecture and microstructure are essential to capitalize on their material superiority and eventually accomplish their mission of surpassing state‐of‐the‐art LIBs.^[^ ^5^, ^201^, ^202^ ^]^ Comprehensive understanding of the physical and electrochemical processes at the micro‐scale is critical to rationalize the microstructural engineering strategy for different applications.^[^ ^131^, ^203^ ^]^ Nowadays, extensive use of nanomaterials and related techniques achieves high capacities that surpass those of conventional electrodes.^[^ ^171^ ^]^ From the perspective of either engineering or chemistry, it is undoubtedly intelligent to learn from nature to remove the barriers to development.^[^ ^21^, ^22^, ^23^ ^]^ Natural structural materials are constructed from a relatively limited array of components at typical operating temperatures. While bio‐inspired materials are lightweight and frequently exhibit unique combinations of strength and toughness, their synthetic replication has posed considerable challenges.^[^ ^24^, ^204^ ^]^ With characteristic dimensions ranging from the nanoscale to the macroscale, they usually comprise soft and hard phases arranged in complex hierarchical architectures and thus achieve the special functions.^[^ ^205^ ^]^ The bio‐inspired concept will be introduced in bio‐inspired electrode design and preparation to improve the electrochemical performance and thermal safety of LIBs.

Electrode architecture can strongly affect its properties and performance in a complex manner.^[^ ^206^ ^]^ Based on the bio‐inspired structures from our lives, smart electrodes achieve a specific function in terms of electrochemical performance, flexible deformation, recycling function, and thermal safety. The electrodes containing various additive materials can obtain different specific capabilities. Smart features of electrodes (including self‐healing, fast ion transport, self‐extinguishing, thermoresponsive switching, easy‐to‐recycle function, and flexible deformation) can be achieved via coating electrode (cathode or anode) particles, developing specific binders, current collectors, and microcapsules, and bio‐inspired structures, which are classified in **Table** **1**. Many factors influence the morphology and growth of nanostructures in electrodes, encompassing the selection of precursors (salts, co‐precursors, and solvents), reaction conditions (concentration, duration, temperature, pressure, and pH), synthesis method (bulk, templated, and directly grown on current collectors), and structural modifications after post‐treatment and post‐cycling.^[^ ^207^ ^]^ A rational combination of architectural design and material strengths, considering the characteristics of both structures and materials to match each other, enable to bring maximum benefits. The bio‐inspired structures and their functions will be discussed in the following sections.

**Table 1:** Factors involved in bio‐inspired electrodes with spatiotemporal management.

Spatiotemporal management	Self‐healing	Fast ion transport	Self‐extinguishing	Thermoresponsive switching	Easy‐to‐recycle function	Flexible deformation
Cathode	√	√	√	√		
Anode	√	√	√			
Binder	√		√			
Current collector	√		√	√	√	√
Microcapsule	√		√	√		
Bio‐inspired structure	√	√	√	√	√	√

## 3 Self‐Healing Electrodes

Developing novel electrode materials with self‐healing capabilities to repair internal or external damages is a crucial and highly effective strategy for mitigating the degradation of LIBs.^[^ ^208^ ^]^ Self‐healing is the ability to restore damage naturally, such as stopping bleeding, skin wound healing, and repair of broken bones, which is a significant survival feature that increases the lifetime of most creatures.^[^ ^34^, ^209^ ^]^ Self‐healing capabilities are applied in the various areas of material science. To prolong the lifetime of rechargeable LIBs, this feature is highly desirable for the bio‐inspired electrode design. Herein, modification strategies of electrodes include structure optimization, surface/interface regulation, alloying, nano‐crystallization, compositing, novel binders, and innovative design of electrolyte.^[^ ^148^, ^210^ ^]^

Self‐healing mechanisms includes autonomous and non‐autonomous, and thus their behaviors enable to be classified into the physical, chemical, and physical–chemical synthetic approaches.^[^ ^53^, ^131^ ^]^ Therein, self‐healing materials applied in LIBs presently have primarily used chemical approaches to achieve self‐healing capability. The chemical approaches are either reversible chemical bonds or supramolecular interactions. The self‐healing materials can achieve specific healing behaviors based on various self‐healing mechanisms, such as reversible covalent bonds, reversible non‐covalent bonds, microcapsules, etc.^[^ ^211^ ^]^ Furthermore, the element doping, surface coating, and optimization of the synthesis process in electrode materials enable to achieve significant electrochemical performance improvement on super high discharge capacity or capacity retention of LIBs.^[^ ^100^ ^]^

Bio‐inspired electrodes are developed with self‐healing core–shell structure,^[^ ^30^, ^31^, ^32^, ^33^, ^34^, ^35^ ^]^ self‐healing binders,^[^ ^36^, ^37^, ^38^, ^39^, ^40^ ^]^ liquid metal alloys,^[^ ^41^, ^42^, ^43^, ^44^, ^45^, ^46^ ^]^ microcapsules,^[^ ^48^, ^49^, ^50^ ^]^ and current collectors.^[^ ^51^ ^]^ These electrodes owning self‐healing capabilities possess much more stable mechanical characteristics than conventional electrode materials that can be shortened their durability by mechanical fractures generated during cycling. These capabilities can repair the damages of bio‐inspired electrodes, and thus enhance the lifetime and long‐term cycling stability while simultaneously resolving economic and safety issues.^[^ ^212^ ^]^ All the same, there is still vast development potential for the relentless exploitation of self‐healing electrodes. And hence, it is looking forward to combining multiple modifications in bio‐inspired electrodes as a breakthrough direction to further pursue the higher power density, energy density, and longer lifetime of LIBs.

### 3.1 Coating Electrode Particles

The interfacial stability of Ni‐rich cathodes, Si‐containing anodes, and Sn‐based anodes is the key to producing high‐energy LIBs.^[^ ^213^, ^214^ ^]^ Modifying the structure of the electrode materials is an efficient approach to suppress the pulverization of electrodes.^[^ ^215^ ^]^ Herein, the coating electrode particles are inspired by core–shell structures like cells, seeds, eggs, and fruits. In these natural products, their shells can protect the core under stress, and thus they will live for a long time. Similarly, the shells of coating electrode particles can prevent the electrode particles pulverization during the lithiation–delithiation cycling processes without affecting the electrochemical performance of LIBs. As a result, the core–shell structured electrodes enable the improvement of their electrochemical performance in terms of rate capability, cycling stability, and scalability for large‐scale production of LIBs.^[^ ^35^, ^216^ ^]^

#### 3.1.1 Core–Shell Structured Cathode Particles

**Figure 4:**
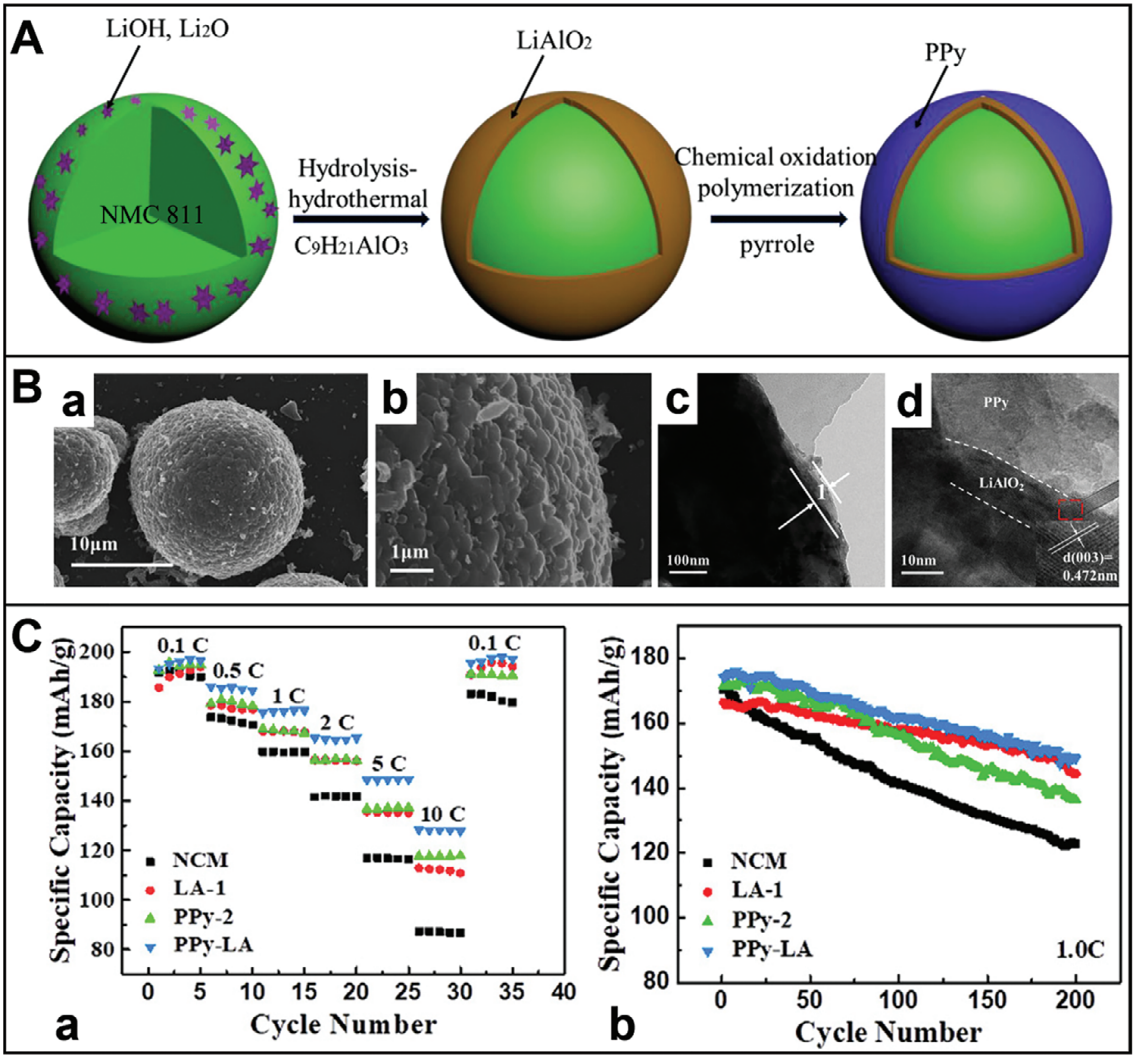
A) Synthesis schematic and micro‐structures of dual‐conductive coating of NMC 811 particle with PPy and LiAlO_2_. B) SEM images of a,b) PPy–LA; TEM images of c,d) PPy–LA samples. HRTEM image d) of the selected area 1 in c. C) a) Rate capability; b) Cycling performance of pristine NMC 811, LA‐1, PPy‐2, and PPy–LA samples.^[^ ^33^ ^]^ Copyright 2020, Journal of Alloys and Compounds.

Both poor rate capability and insufficient cycling stability have been considered the greatest obstacles to the extensive commercialization of Ni‐rich layered cathode materials. The design and modification methods of the ion pathways include coating, composite pathways, element doping, and new functional structures.^[^ ^6^, ^217^ ^]^ Herein, the cycling lifetime of LIB can be improved by coating the cathode particle with oxide, polyimide, inorganic particles, and conducting polymer.^[^ ^30^, ^31^, ^32^ ^]^ A fraction of the cathode particles is affected by the delithiation process, which creates local inhomogeneities for cathode film.^[^ ^218^ ^]^ Thus, developed bio‐inspired cathodes owning self‐healing capabilities are significant for advanced LIBs, and core–shell structured electrodes possess excellent electrochemical performances and thermal safety. The general coating Ni‐rich cathode active materials include LiNi_0.8_Mn_0.1_Co_0.1_O_2_ (NMC 811),^[^ ^33^, ^219^, ^220^, ^221^, ^222^, ^223^, ^224^ ^]^ LiNi_0.6_Mn_0.2_Co_0.2_O_2_ (NMC 622),^[^ ^225^, ^226^, ^227^ ^]^ LiNi_0.5_Mn_0.3_Co_0.2_O_2_ (NMC 532),^[^ ^228^, ^229^ ^]^ LiNi_0.4_Mn_0.4_Co_0.2_O_2_ (NMC 442),^[^ ^230^ ^]^ Li_1.2_Ni_0.13_Mn_0.54_Co_0.13_O_2_ (NMC 13),^[^ ^231^, ^232^, ^233^, ^234^, ^235^, ^236^, ^237^ ^]^ LiNi_0.85_Mn_0.10_Co_0.05_O_2_ (NMC 8510),^[^ ^238^ ^]^ LiNi_0.85_Mn_0.05_Co_0.1_O_2_ (NMC 8505),^[^ ^239^ ^]^ LiNi_0.83_Mn_0.06_Co_0.11_O_2_ (NMC 83),^[^ ^240^ ^]^ LiNi_0.5_Mn_1.5_O_4_ (LNM),^[^ ^241^, ^242^ ^]^ LiCoO_2_ (LCO),^[^ ^243^ ^]^ etc.^[^ ^244^, ^245^, ^246^, ^247^ ^]^ For instance, polypyrrole–LiAlO_2_ (PPy–LA) coats NMC 811 composites based on a dual‐conductive coating strategy (**Figure** **4**).^[^ ^33^ ^]^ The core–shell structured NMC 811 particles are synthesized via in situ chemical polymerization method, and hydrolysis hydrothermal approach. Figure 4A displays the fabrication process of the PPy–LA sample with double shells. Al(OH)_3_ reacts with LiOH to manufacture an initial coating layer on the surface of NMC 811 particles. Then the NMC–LA attained using annealing, as shown below:

(1) $$\mathrm{Al}{\left(\mathrm{OH}\right)}_{3}\;+\;H_{2}O\;+\;\mathrm{LiOH}\;\overset{\mathrm{Hydrothermal}}{\to }\;\mathrm{LiAl}{\left(\mathrm{OH}\right)}_{4}\cdot H_{2}O$$

(2)

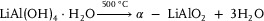




In the final step, PPy is applied onto the surface of the NMC–LA particle through in situ chemical polymerization. This process forms an LA coating, which effectively suppresses side reactions and enhance ionic conductivity. Additionally, the PPy‐coated cathode particles enhance electronic conductivity in the composite materials. This layer mitigates the growth of internal resistance stemming from the uncontrollable evolution of the cathode−electrolyte interface (CEI) and acts as a channel for lithium‐ion diffusion during cycling.^[^ ^103^ ^]^ Consequently, this cathode configuration exhibits an impressive capacity retention ratio of 92.8% after 100 cycles, alongside a high capacity of 128 mAh g^−1^ at 2 A g^−1^ (Figure 4C). Leveraging double conductive surface engineering preserves the structural integrity of NMC 811 while facilitating ensure the rapid diffusion of electrons/lithium‐ions.^[^ ^221^ ^]^ Thus, the double‐shell structured cathode demonstrates outstanding cycling stability and rate capability.

Moreover, applying a few nanometers thick Al_2_O_3_ coating on NMC particles has a beneficial effect on electrochemical performance.^[^ ^248^ ^]^ This protective layer prevents electrolyte side reactions with highly active Ni oxide sites, facilitating charge compensation through Ni oxidation and enabling high‐voltage cycling stability.^[^ ^230^ ^]^ Despite a high surface impedance, the Al_2_O_3_ coating remains stable during cycling, contributing to improved cycling stability.^[^ ^227^, ^236^, ^245^ ^]^ Additionally, uncoated thin layers of NMC particles are prone to corrosion and mechanical damage, leading to crack formation and increased surface roughness. Therefore, amorphous Al_2_O_3_‐coated NMC 532 particles synthesized via carboxymethyl cellulose (CMC)‐assisted wet chemical method exhibit enhanced performance. This cathode configuration maintains a capacity retention of 86.30% after 100 cycles at 1C, surpassing that of pristine NMC 532 (76.40%).^[^ ^229^ ^]^ Moreover, TiO_2_ is another coating material for cathode particles.^[^ ^249^ ^]^ Nanolayers of TiO_2_ and Al_2_O_3_ coatings are applied to lithium‐ and manganese‐rich cathode NMC 13 particles using an atomic layer deposition method.^[^ ^231^ ^]^ The TiO_2_ layers appear as particulates dispersed across the particle surfaces, while the Al_2_O_3_ surface layer exhibits a conformal and uniform appearance. The TiO_2_ layer exhibits increased reactivity with Li, forming a Li*_x_* TiO_2_ interface that contributes to a modest enhancement in rate capability. Additionally, Li_3_BO_3_ nanoparticles on the cathode surface suppress side reactions, forming a protective layer between the cathode surfaces and electrolytes, thereby enhancing structural stability.^[^ ^238^ ^]^ This layer facilitates lithium‐ion diffusion across the cathode surface as a lithium‐ion conductor and enhances the electron conductivity of the cathode, thereby mitigating impedance growth during cycling. Li_3_BO_3_‐coated NMC 8510 particles are synthesized via the conventional coprecipitation method, followed by a wet chemistry process. NMC 8510 coated with 2 wt.% Li_3_BO_3_ exhibits significantly improved capacity retention, reaching 90.1% after 300 cycles at 45 °C, compared to pristine particles, which retain only 80.4% under similar conditions. Additionally, HfO_2_ nanoparticles serve as secondary particles, coating the surface of NMC 8505 particles, thereby enabling these cathodes to effectively mitigate electro–chemo‐mechanical degradation.^[^ ^239^ ^]^

Moreover, in LIBs, generating large amounts of reactive Ni^4+^ can lead to direct reactions with the electrolyte at high voltages, forming thick CEI layers.^[^ ^35^, ^250^, ^251^, ^252^ ^]^ Lithium borate dioxalate (LiBOB) is an effective electrolyte additive for Ni‐rich cathodes. An electrolyte layer‐forming additive (Al(Li)BOB nanolayer) is grown on individual NMC 811 surfaces through lithium consumption, developing an organic CEI layer rich in boron–oxygen bonds during cycling.^[^ ^253^ ^]^ Compounds such as Li_2_CO_3_, Li*_x_* PO*_y_* F*_z_*, Li*_x_* PF*_y_*, and LiF are predominant in the CEI of Ni‐rich materials.^[^ ^226^, ^247^, ^254^ ^]^ LiF is an exceptional protective layer on the cathode surface, impeding direct contact between the cathode and electrolyte, thus stabilizing the cathode surface structure.^[^ ^243^ ^]^ Consequently, the electrochemically stable LiF‐rich surface facilitates the formation of a favorable CEI layer, effectively preventing electrolyte attack. LiNO_3_, NaF, and H_3_BO_3_ are utilized to construct outer LiF‐rich layers and inner B, F gradient‐doped hierarchical nanostructures.^[^ ^255^ ^]^ The gradient doping of B and F in the subsurface region enhances lattice stability at high temperatures and potential, thereby increasing cycling stability. The unique core–shell structured LCO demonstrates stable cycling at a high voltage of 4.6 V with a capacity retention of 93.2% after 800 cycles and 95.2% retention after 100 cycles at a high temperature of 45 °C. The coating layer surrounding the cathode surface increases LIB lifetime and retains a larger capacity at high and low temperatures.

The oligomer is a potential material for coating the Ni‐rich oxide cathode material particles to improve electrochemical performance and thermal safety. Lithium‐containing hybrid oligomer is synthesized through the polymerization of bismaleimide with a polyether monoamine, trithiocyanuric acid, and LiOH.^[^ ^219^ ^]^ It can be coated on the surface of Ni‐rich NMC811 particles. Further, polyimide is a special functional polymer and possesses high mechanical strength, excellent chemical inertness, and extremely high thermal stability.^[^ ^256^, ^257^ ^]^ The thin polyimide layer is coated on the surface of NMC 13 particles using poly(acrylic acid) (PAA).^[^ ^29^ ^]^ The polyimide coating layer (≈3 nm) effectively separated core–shell structured NMC 13 particles from the electrolyte and stabilized CEI at high voltage (≥ 4.5 V), leading to better cycling stability and rate capability.^[^ ^232^ ^]^ NMC 532 particle surfaces coated with a 1 wt.% bismaleimide/trithiocyanuric acid oligomer possess a capacity retention of 91% to the bare NMC 532 after 30 cycles at 0.1C.^[^ ^228^ ^]^ For insight into thermal safety, such cathode without electrolyte disintegrates at a higher temperature than the bare one (317 vs 284 °C). Furthermore, the total heat generation of the prepared cathode is much lower than the bare cathode (599 vs 824 J g^−1^). The surface modification is one of significant ways to enhance high‐temperature electrochemical performance of cathode as well.^[^ ^258^ ^]^ Consequently, the cathode particle coating polymer could widen the range of its decomposition temperatures while reducing the heat generated simultaneously.

In summary, the bio‐inspired design strategy of core–shell structures protects structural stability and reduces side reactions at the cathode interface. The protective and buffering effects of the coating layer result in LIBs exhibiting exceptional electrochemical performance, including superior rate capability and cycling stability. Furthermore, employing a dual‐coating layer strategy enhances the electrochemical performance of Ni‐rich cathodes. Additionally, integrating Al‐based materials effectively enhances the thermal stability of Ni‐rich cathodes. Coating an electrolyte additive onto the surface of cathode particles facilitates the formation of a robust CEI. Moreover, cathode partilces coated with polymer show promise for high‐energy LIB applications without susceptibility to thermal runaway. These approaches pave the way for the commercialized design and synthesis of Ni‐rich layered cathode materials.

#### 3.1.2 Core–Shell Structured Anode Particles

Si offers a capacity of more than an order of magnitude higher than graphite, but it undergoes significant volume changes during cycling, often resulting in rapid anode degradation.^[^ ^134^ ^]^ The poor cycling stability of LIBs, attributable to the substantial volume expansion during lithiation, is a critical challenge for anodes.^[^ ^113^ ^]^ Employing various coating matrices to modify the anode is a promising strategy for addressing these issues.^[^ ^259^, ^260^, ^261^, ^262^ ^]^ Applying a coating approach to the bio‐inspired yolk–shell structured anodes is promising for enhancing the electrochemical performance of anode materials with low conductivity and high volume expansion during cycling.^[^ ^214^, ^263^, ^264^, ^265^, ^266^ ^]^ Supramolecular chemistry and mechanostereochemistry provide unique tools for maintaining interparticle interaction even during the drastic volume change of Si particles.^[^ ^38^ ^]^ The randomly branched hydrogen bonding polymer can spontaneously repair cracks and damage in the coating during cycling.^[^ ^34^ ^]^ Anode composites can be classified into 0D (nanoparticles), 1D (nanowires, nanotubes, nanofibers, and nanorods), 2D (nanoplates and nanosheets), and 3D (micro‐nanostructures assembled by 1D or 2D nanostructures) materials based on the stereo‐structure of Si.^[^ ^267^, ^268^, ^269^ ^]^ Thus, the core–shell structured anodes coated with self‐healing materials possess both a higher cycling lifetime and excellent electrochemical performance.

**Figure 5:**
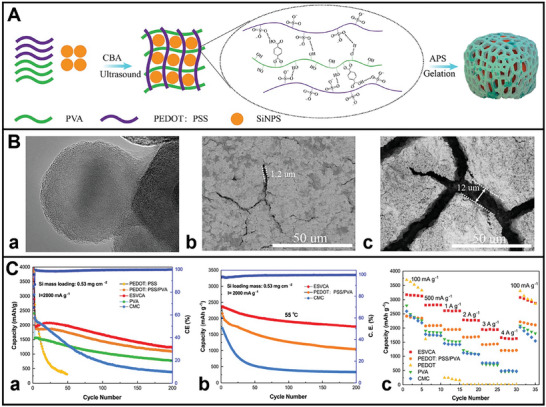
A) Schematic illustration of the fabrication process for Si anode with self‐healing conductive hydrogel binder. B) a) TEM image of Si particle coated self‐healing conductive binder. SEM images of Si anodes containing b) ESVCA, and c) CMC after 200 cycles. C) Electrochemical performance of Si anodes containing different binders. a) Cycling performance of the Si@ESVCA, Si@PEDOT:PSS/PVA, Si@PEDOT:PSS, Si@PVA, Si@CMC anodes at 2 A g^−1^. b) Cycling performance of Si anodes containing different binders at 55 °C. c) The rate capability of Si anodes containing different binders at different current densities from 0.1 to 4 A g^−1^.^[^ ^36^ ^]^ Copyright 2020, Journal of Power Sources.

The core–shell structured anode particles can maintain the integrity of the anode and achieve self‐healing capability.^[^ ^256^, ^270^ ^]^ The main types of silicon coatings include inorganic, organic, carbon, binder materials and double layer coatings.^[^ ^32^, ^271^, ^272^ ^]^ The emerging binder is elastic and adhesive, enabling it to accommodate the significant volume changes of the Si anode while maintaining its structural integrity.^[^ ^273^ ^]^ Through a codissolution method, the optimized binder and electrolyte can develop a nanolayer on the surface of Si, facilitated by beneficial functional groups.^[^ ^274^ ^]^ Consequently, a novel 3D network self‐healing conductive hydrogel (ESVCA) binder comprising poly(3, 4‐ethylenedioxythiophene): poly(styrenesulfonate) (PEDOT: PSS) polymer and poly(vinyl alcohol) (PVA) polymer has been developed (**Figure** **5**A).^[^ ^36^ ^]^ The PEDOT coating layer effectively suppresses the volume expansion of Si and establishes a cross‐linked conductive network on its surface.^[^ ^112^ ^]^ This 3D network self‐healing conductive hydrogel binder demonstrates exceptional stretchability, rapid self‐healing capability, and high conductivity under typical operating temperatures. In the bio‐inspired anode, the stretchable 3D self‐healing network structure coating on the surface of Si particles (Si@ESVCA) efficiently mitigates the considerable volume changes experienced during the lithiation–delithiation cycling process (Figure 5B). Si@ESVCA anodes exhibit outstanding cycling stability, retaining 74.1% capacity and maintaining a high reversible capacity of 1743 mAh g^−1^ after 200 cycles at 2 A g^−1^ at elevated temperatures (Figure 5C). Consequently, this coating maintains the mechanical integrity of Si anodes while ensuring the presence of 3D continuous electron transport pathways that promote high conductivities and improved electrical contacts among active Si particles. The emerging binder embedding both 3D conductive pathways and fast self‐healing capability is promising for application in high‐capacity anode materials for next‐generation high‐energy LIBs.

Besides, the polymethyl methacrylate (PMMA) shell coats the Si particle via microemulsion polymerization, and thus the bio‐inspired anode has high elasticity, high lithium conductivity, a certain viscosity in electrolytes, and excellent electrolyte retention capability.^[^ ^275^ ^]^ Further, the amino‐functionalized Si and dopamine‐modified PAA are used to prepare the Si anode network structure based on supramolecular self‐assembly as well.^[^ ^276^ ^]^ It is a multifunction dynamic cross‐linking strategy for bio‐inspired anodes possessing self‐healing chemistry and enhanced ionic conductivity. The dynamic reversible hydrogen and ionic bonds provide the anode with strong adhesion and self‐healing capability. Furthermore, the polar groups present can significantly improve the transport kinetics of lithium ions. Thus, a multifunctional polymeric binder is synthesized by crosslinking conducting polymer and stretchable polymer poly(ether‐thioureas).^[^ ^277^ ^]^ This results in an interweaving continuous 3D network formed by the multifunctional polymeric binder, which wraps around the surfaces of nano Si particles, facilitating electron transmission and ensuring mechanical stability.^[^ ^278^ ^]^ Leveraging the excellent binding strength of polydopamine (PDA), it can serve as a nanobinder to effectively adhere active and conductive materials together.^[^ ^279^ ^]^ The PDA layer acts as a bionic ionic permeable layer. It can be coated onto the surfaces of ZnFe_2_O_4_ particles through the self‐polymerization of dopamine in the presence of an alkaline buffer solution.^[^ ^280^ ^]^

The SEI, a crucial component, forms as a passivation layer on the surface of anode materials owing to electrolyte decomposition.^[^ ^281^, ^282^, ^283^ ^]^ In the first cycle, the anode develops the SEI and undergoes prelithiation.^[^ ^142^ ^]^ The presence of an SEI layer enhances the anode's thermal stability and mechanical properties, thereby improving thermal safety and cycling stability.^[^ ^17^, ^125^, ^284^ ^]^ An artificial SEI with uniform and elastic properties can effectively accommodate volume changes while mainntaining SEI layer stability, reducing resistance, facilitating electron transfer and lithium‐ion migration, and enhancing structural stability.^[^ ^285^, ^286^, ^287^ ^]^ Polymeric materials possessing self‐healing capabilities, can autonomously repair damage to prevent anode cracking and pulverization or stabilize the SEI layer.^[^ ^212^ ^]^ An artificial SEI can consist of poly‐(1,3‐dioxolane) and high‐modulus fluorinated products formed from the in situ decomposition of Li salts.^[^ ^288^ ^]^ This flexible layer acts as a stress‐relieving barrier, accommodating the significant volume changes experienced by the Si anode during lithiation–delithiation cycling. Simultaneously, a high electronic‐conductivity Ni–P alloy and high elasticity styrene‐butadiene rubber are coated onto the surface of the Si anode.^[^ ^289^ ^]^ The alloy disperses around the Si particles, enhancing the electronic conductivity of the anode. In contrast, the styrene‐butadiene rubber distributed around the Si particles enhances the anode's elasticity to manage volume expansion. Consequently, these polymer‐shell structured anodes exhibit superior electrochemical performance, characterized by enhanced cycling stability, high rate‐capability, and reversible capacity.

Enhancing the electrical conductivity and material plasticity of Si particles through carbon modification is a promising method.^[^ ^267^, ^290^, ^291^, ^292^, ^293^ ^]^ Carbon materials own special physical and chemical properties,^[^ ^193^, ^294^, ^295^, ^296^, ^297^ ^]^ and carbon has working potential for rational optimization of SiO*_x_*/carbon composites improves performance.^[^ ^151^, ^298^, ^299^ ^]^ A porous carbon shell is coated onto the Si particle, serving not only to maintain excellent interface contact between the Si particle and graphite but also to enhance electrolyte infiltration, lithium‐ion diffusion, and composite anode conductivity.^[^ ^300^, ^301^, ^302^ ^]^ The porous structure acts as a buffer, mitigating structural strains and reducing excessive pulverization of the anode materials.^[^ ^303^ ^]^ Si nanoparticles enclosed in hollow carbon tubes create space between them, allowing the anode to achieve high gravimetric capacity and prolonged cycling lifetime.^[^ ^141^, ^304^ ^]^ An in situ doped Si/carbon anode material can be synthesized using SiO_2_ and CO_2_ as Si and carbon sources and Mg as the reduction medium (Mg_2_Si + CO_2_ → MgO + Si + C).^[^ ^305^ ^]^ Consequently, the embedded carbon facilitates the nanosizing of Si (less than 50 nm) and forms a 3D carbon network, acting as a buffer layer and enhancing electrical conductivity.^[^ ^149^, ^306^ ^]^ An anode simultaneously equipped with the SiO_2_ layer and carbon scaffold exhibits an excellent reversible capacity of 1071 mAh g^−1^ and retains 70% of its capacity after 200 cycles at 0.42 A g^−1^.^[^ ^307^ ^]^

Further, the emerging multi‐shell core anodes,^[^ ^308^ ^]^ such as double carbon‐shelled Si nanoparticles, exhibit high rechargeable specific capacity, superior rate capability, and excellent cycling performance for up to 1000 cycles.^[^ ^309^ ^]^ The inner flexible carbon shell creates finite inner voids to accommodate the substantial volume changes of Si particles, while the outer rigid shell facilitates the formation of stable SEI and preserves structural integrity.^[^ ^310^ ^]^ Notably, the preserved inner‐shell spaces buffer volume changes and alleviate mechanical stress from the inner carbon shell. Additionally, carbon shells enhance conductivity and significantly reduce the charge transfer resistance of Si‐based anodes.^[^ ^154^, ^311^, ^312^, ^313^ ^]^ The double‐shelled hollow structures effectively tune the vigorous volume change, facilitates the formation of a highly stable SEI layer, shortens the electron/lithium‐ion transport distances and provides fast electron transport in the interconnected hollow structure.^[^ ^314^ ^]^ Thus, a compact micron‐sized composite anode with a tight binding and double‐shell architecture possesses superior deformation resistance and electrical conductivity, contributing to excellent cycling stability and good rate capability in a thick electrode.^[^ ^315^ ^]^ A composite anode with hierarchical buffer structure coated Si nanoparticles possesses good rate performance.^[^ ^316^ ^]^ Therein, resorcinol–formaldehyde resin serves as a structural buffer, conductive layer, and provides quick routes for lithium‐ion diffusion and electron transfer inside. It also accommodates the volume change of Si. The special turbostratic structure of mesophase pitch effectively improves the structural stability and conductivity of the anodes.

Natural spherical graphite, widely employed as an anode material in LIBs,^[^ ^299^, ^317^ ^]^ can perform better when coated with MnCl_2_.^[^ ^115^ ^]^ This coating forms a cladding layer with internal pores on the surface, enhancing the prepared anode's charging/discharging capacity and rate capability. These improvements surpass those observed in natural spherical graphite mixed solely with MnCl_2_. Moreover, the carbon coating layer plays a dual role: it suppresses the release of intercalated lithium from natural graphite at high temperatures and shields the graphite structure from electrolyte attack.^[^ ^318^ ^]^ The ternary composite anode with a 3D triple buffering structure possesses an enhanced electrochemical performance and higher rate capability.^[^ ^116^ ^]^ Thus, an emerging Si@Li_4_SiO_4_/amorphous carbon/carbon nanotube anode is designed with a 3D network structure.^[^ ^159^ ^]^ The carbon nanotubes as conductive additives can effectively mitigate the anode issues of sluggish kinetics and poor stability.^[^ ^111^, ^144^, ^319^ ^]^ Such anode possesses an exceptionally high initial discharge capacity, prominent rate capability, and excellent long‐term cycling stability. The metal‐organic frameworks possess tunable properties and outstanding morphological and structural advantages.^[^ ^320^ ^]^ The graphene oxide confined Si@Cu core–shell structure serves as an anode, with the Cu shell enveloping the surface of Si particles to enhance electronic conductivity between them and graphene.^[^ ^160^ ^]^ This combination of Cu shell and graphene prevents changes in anode morphology during cycling, ensuring sustained excellent contact between the anode and current collector. The flexible Cu greatly can accelerate the charge transfer and lithium‐ions transfer as well.^[^ ^321^ ^]^ In addition to successfully preventing the aggregation of core‐shell structured anode nanoparticles, cladding graphene also leaves adequate room for Si to expand during lithiation. The 3D redox graphene layer builds a conductive structure that accelerates the reaction kinetics of LIB.^[^ ^112^ ^]^ Such anode possesses a satisfactory electrochemical performance. Therefore, the composite anode has excellent long cycle performance and rate capability, which provides a new idea for the preparation of high‐performance anode materials.

The high specific capacity of Sn as an anode material is an attractive alternative to graphite for next‐generation advanced LIBs.^[^ ^214^ ^]^ However, gradual capacity decay is a persistent issue stemming from particle fracture, pulverization, and cracking induced by the substantial volume changes during the lithiation–delithiation cycling process.^[^ ^322^ ^]^ A 3D structured Sn anode material featurecs large particles composed of nanowires with voids between them, enabling tolerance of volume expansion during lithiation while ensuring close contact between Sn and conducting additives.^[^ ^323^ ^]^ These nanowires, characterized by their small diameter and extended, continuous routes for electron transport, exhibit exceptional rate capability and reduce lithium‐ion diffusion distances. Additionally, a carbon‐coated composite comprising Sn, SnO_2_, and a porous carbon‐nanofiber membrane is utilized to fabricate a 3D nanofiber network structure for the Sn‐based anode.^[^ ^120^ ^]^ The 3D nanofiber network structure enables the composite membrane to function directly as an anode without requiring additional polymer binders or electrical conductors.^[^ ^324^ ^]^ Benefiting from the protective carbon coating and the 3D carbon nanofiber membrane, the Sn–SnO_2_ particle‐based composite anodes exhibit outstanding cycling stability and exceptional rate capability.^[^ ^120^ ^]^ Additionally, interconnected carbon networks anchored with Sn‐core/carbon nanotube shell nanocables in a hierarchical nanostructure yield Sn‐based anodes with superior electrochemical performance.^[^ ^118^ ^]^ The presence of mesopore‐equipped carbon networks facilitates easier electrolyte passage. These unique structures and shapes shorted electronic and ionic transport pathways at the nanoscale, mitigating volumetric expansion and constriction, enhancing nanocomposite conductivity through carbon shells, maintaining structural stability, preventing severe aggregation of the nanostructures, and protecting lithium against dendrite growth.^[^ ^154^, ^317^, ^325^ ^]^

Further, the heterostructural microcube facilitates accelerated lithium‐ion transfer rates by shortening transmission paths.^[^ ^326^ ^]^ Encapsulating heterostructural Sn/SnO_2_ microcube powders with a nitrogen‐doped carbon coating as an anode material results in high initial discharge specific capacity, enhanced rate capability, and enhanced cycling stability.^[^ ^327^, ^328^ ^]^ Nitrogen introduction enhances the electronic conductivity, while the porous structure increases specific surface area.^[^ ^329^ ^]^ Utilizing the metal–organic framework ZIF‐67 as a template and carbon source, with SnCl4 as the tin supply, a new Sn–Co nanoalloy is synthesized to enhance the rate capacity and cycle stability of Sn‐based anodes. This composite, resembling a micro box with a diameter of ≈2 mm and containing uniformly embedded ≈10 nm Sn–Co nanoalloy particles,^[^ ^119^ ^]^ exhibits superior electrochemical performance owing to well‐dispersed, nano‐sized alloy and the buffering effect of porous nitrogen‐doped carbon coating.^[^ ^330^, ^331^ ^]^ The uniform particles remain intact during cycling, contributing to the material's improved electrochemical stability.

In summary, the core–shell structure protects the bio‐inspired anode from pulverization during cycling, while the entire hierarchical structure forms a conductive network enabling rapid electron transportation. The stretchable 3D self‐healing network structure coating on the surface of anode particles can efficiently restrain the vast volume change of anode particles during lithiation–delithiation cycling and thus retain the mechanical integrity of bio‐inspired anodes. The multi‐shell coating layer can effectively reinforce the structural stability and simultaneously enhance the electronic conductivity of bio‐inspired anodes, and control SEI growth. Such core–shell structured anodes are helpful for prelithiation and can stabilize the SEI layer to reduce side reactions. This novel, sustainable, and efficient design of the unique structure is a promising method to obtaining excellent electrochemical performance and cost‐effective composite anodes.

### 3.2 Self‐Healing Binders

#### 3.2.1 Binder Materials

With their high specific capacity, Si‐based and Si/carbon composite anodes are the most promising candidates for developing advanced rechargeable LIBs.^[^ ^55^, ^156^, ^332^, ^333^, ^334^, ^335^ ^]^ However, challenges such as low electrical conductivity, pronounced volume changes during cycling, and unstable SEI significantly impede their application in LIBs.^[^ ^54^ ^]^ Incorporating self‐healing capability, achieved through supramolecular interactions, is crucial for maintaining the structural integrity of Si nanoparticle anodes.^[^ ^38^ ^]^ Traditional binders like polyvinylidene fluoride (PVDF), prevalent in the battery industry, suffer from recognized limitations, including limited binding strength owing to inadequate mechanical properties, lack of chemical bonds with electroactive materials, and low electronic and lithium‐ion conductivities.^[^ ^157^ ^]^ Emerging binders, comprised of developed molecules and polymers possessing intrinsic self‐healing capabilities based the dynamic supramolecular assembly,^[^ ^54^ ^]^ such as hydrogen bonds, electrostatic crosslinking, and host–guest or van der Waals interactions,^[^ ^57^, ^133^, ^156^ ^]^ offer promising solutions. These self‐healing binders maintain electrode integrity through mechanical properties and interactions with electrode surfaces.^[^ ^158^ ^]^

**Figure 6:**
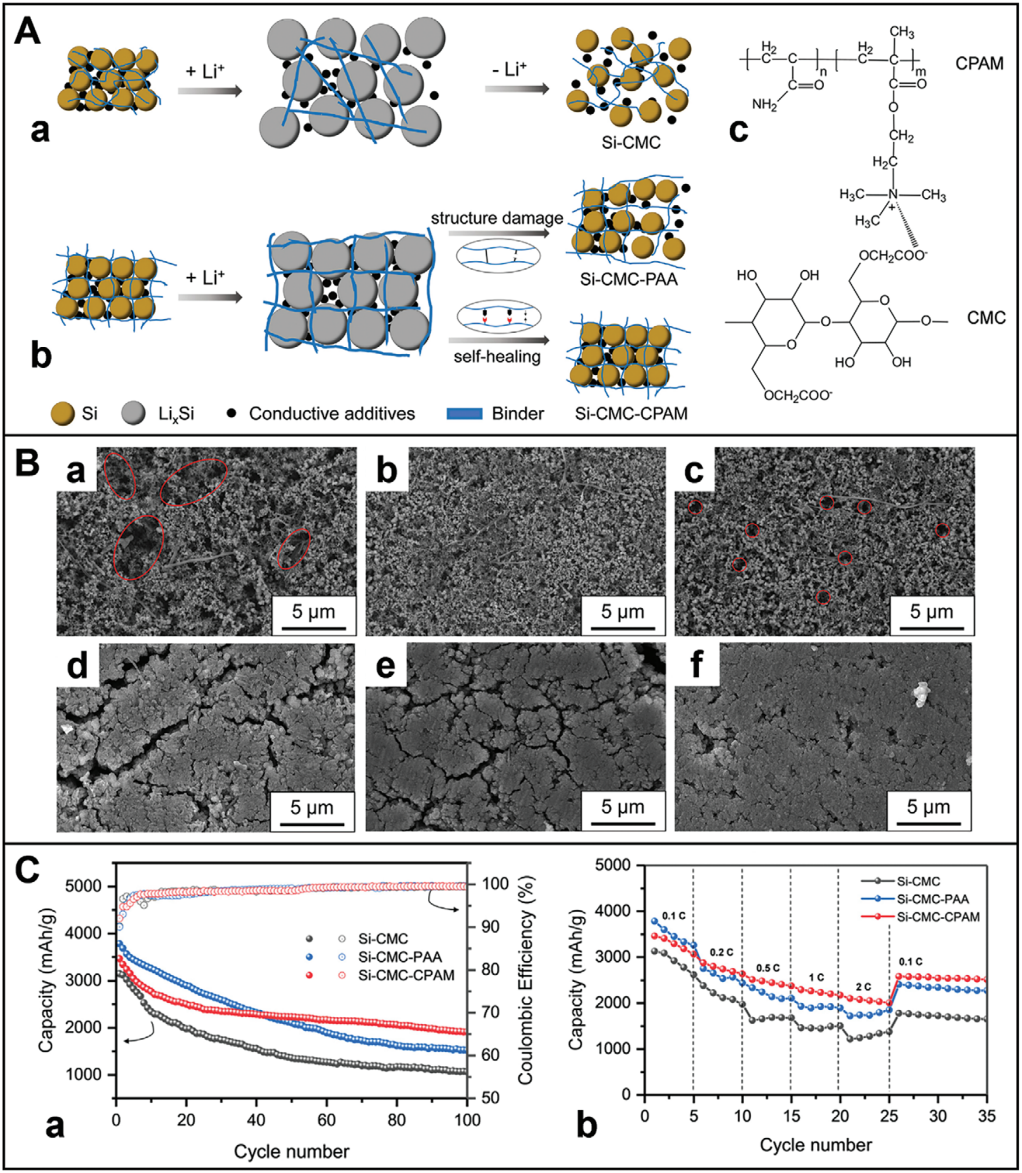
A) Schematic illustrations of the working mechanism of a) linear CMC binder, and b) covalent cross‐linked CMC–PAA binder and self‐healing CMC–CPAM binder. c) Schematic illustration of the electrostatic interaction induced cross‐linking between CMC and CPAM. B) SEM images of surface micro‐structures of a) Si–CMC, b) Si–CMC–PAA, and c) Si–CMC–CPAM anodes before cycling and d) Si–CMC, e) Si–CMC–PAA, and f) Si–CMC–CPAM anodes after 100 cycles. C) Electrochemical performance. a) The long‐term cycling behavior at 0.3 A g^−1^, and b) rate capabilities at 0.1, 0.2, 0.5, 1, and 2C.^[^ ^39^ ^]^ Copyright 2020, Journal of Colloid and Interface Science.

The comprehensive design of multifunctional binders involves integrating various structures, interactions, crosslinking chemistries, ionic or electronic conductivities, and soft and hard segments.^[^ ^336^, ^337^ ^]^ One example of a cycling robust network binder is the composite of carboxymethyl cellulose (CMC) and cationic polyacrylamides (CPAM) (CMC–CPAM). This binder achieves efficient self‐healing capability for Si anode particles through reversible electrostatic interactions between CMC and CPAM (**Figure** **6**A).^[^ ^39^ ^]^ SEM images depicting Si anode surfaces coated with different binders before and after charge–discharge cycling are shown in Figure 6B. The self‐healing CMC–CPAM binder effectively repairs structural damage in the anode. The Si anode mixed with the self‐healing CMC–CPAM binder exhibits superior cycling stability compared to the covalently crosslinked CMC–PAA and linear CMC binders (Figure 6C). This anode maintains a remaining capacity of 1906.4 mAh g^−1^ after 100 cycles. While strong bonding of binders contributes to better morphological control of anodes, supramolecular interactions (weaker strength) have proven more beneficial for long‐term cycling lifetime compared to covalently crosslinked binders (higher strength) lacking supramolecular interactions or dynamic components.^[^ ^38^ ^]^ Si‐based anodes mixed with this novel functional binder demonstrate excellent cycling stability. The enhanced electrochemical performance can be attributed to the synergistic effect of superior mechanical adhesive strength and the self‐healing capability of the 3D network binder.

A novel binder, Al/Alg–poly(ether‐thioureas) (Al/Alg–TUEG), incorporates hydrogen bonding and Al–O coordination bonds.^[^ ^338^ ^]^ The ether oxygen groups of TUEG reduce charge transfer resistance between the electrolyte and Si nanoparticles, facilitating faster transport of lithium ions and enhancing conductivity in the Si@Al/Alg–TUEG anode. Consequently, this anode demostrates outstanding electrochemical performance, retaining 77.4% of its capacity after 300 cycles at 0.5C. With the formation of a uniform and stable SEI layer, the initial specific capacity reaches 3204 mAh g^−1^. Moreover, the LiFePO_4_‐equipped LIB featuring Si@Al/Alg–TUEG achieves a capacity retention of 94% after 100 cycles. The LIB equipped with NMC 811 and Si@Al/Alg–TUEG also possesses excellent electrochemical performance with capacity retention of 92.5%. As a result, polymer binders acquire a quick self‐healing capability that includes both hydrogen bonding and metal coordination at typical working temperatures. And with improved cycle stability in LIBs, it has already demonstrated considerable promise for commercial applications in Si‐based anodes.

**Table** **2** provides a comparative ananlysis of various self‐healing binders utilized for bio‐inspired anodes. Establishing robust interactions between Si particles and the binder (via hydrogen or covalent bonds) is essential for maintaining the anode integrity.^[^ ^339^ ^]^ PAA as an additive material and hydrogen bonds are employed in anodes to achieve self‐healing capabilities. The self‐healing binder incorporates non‐covalent and reversible covalent bonds, effectively enhancing the cycling stability of LIBs by repairing internal and external damage resulting from the substantial volume change of Si‐based anodes.^[^ ^156^ ^]^ Rational design and utilization of natural biomass‐based or synthetic binders, can significantly enhance the cycling performance and SEI stability of Si‐based anodes. LIBs incorporating a bio‐inspired anode with self‐healing binders demonstrate remarkable capacity retention, reaching 95% after 600 cycles at 0.5 A g^−1^. The multifunctional polymeric binder is a promising avenue for advancing superior electrochemical performance owing to its high conductivity, flexibility, and self‐healing capacity. Compared to conventional binders, self‐healing binders feature reversible bonds with dynamic sensitivity at the molecular level, enabling rapid repair of microcracks in anodes during long‐term cycling.^[^ ^54^ ^]^ These binders are crucial in maintaining anode integrity, leveraging their mechanical properties and interactions with anode particle surfaces.^[^ ^158^ ^]^ Consequently, such binders are promising for enhancing the electrochemical performance of bio‐inspired anodes.

**Table 2:** Self‐healing effect via supramolecular interactions for bio‐inspired anodes.

Self‐healing material	Healing bond	Electrode material	Electrolyte	Performance	Year	Reference
PEG	H‐bond	Si microparticle anode	1 M LiPF_6_ in EC/DEC/FEC (1:1:0.1 vol.%)	80% capacity retention after 150 cycles at 0.5C	2018	[340]
PVD–SBR	H‐bond	Si/graphite anode	1 M LiPF_6_ in EC/DEC/EMC (2:3:1 vol.%)	71.7% capacity retention after 100 cycles at 0.5C	2018	[341]
PAA–UPy	Quadruple H‐bond	Si anode	EC/EMC/DMC (1:1:1 vol.%)	2638 mAh g^−1^ after 110 cycles at 0.5C	2018	[342]
Fe^3+^–(tris)catechol	Fe^3+^–(tris)catechol coordination bond	Si nanoparticle anode	1 M LiPF_6_ in EC/DEC (1:1 vol.%) with 10 wt.% FEC	81.9% capacity retention after 350 cycles at 1C	2019	[343]
PAA–PR	π–π stack and H‐bond	Si anode	1 M LiPF_6_ in EC/DEC (1:1 vol.%)	82.5% capacity retention after 150 cycles at 0.5C	2019	[344]
Alg–C–chitosan	H‐bond	Si anode	1 M LiPF_6_ in EC/DEC/DMC (1:1:1 vol.%)	60.6% capacity retention after 160 cycles at 0.1 A g^−1^	2019	[345]
PVA–CBA/PEDOT:PSS	H‐bond	Si anode	1 M LiFP_6_ in EC/DEC (1:1 wt.%) with 10% FEC	71.3% capacity retention after 200 cycles at 0.5 A g^−1^	2020	[36]
FPAA	Thermal Diels‐Alder (D‐A) click chemistry	Si anode	1 M LiPF_6_ in EC/DEC (1:1 vol.%) with 10 wt.% FEC	1076 mAh g^−1^ after 200 cycles at 0.5C	2020	[346]
UPy–PAA–PEG	H‐bond	Si anode	1 M LiPF_6_ in EC/DEC (1:2 vol.%) with 10 wt.% FEC	1450.2 mAh g^−1^ after 350 cycles at 0.5C	2020	[347]
PAA–BFPU	H‐bond	Si anode	1 M LiPF_6_ in EC/DEC (1:1 vol.%) with 10 wt.% FEC	88% capacity retention after 200 cycles at 2 A g^−1^	2020	[348]
CMC–CPAM	H‐bond	Si‐based anode	1 M LiPF_6_ in EC/DEC/EMC (1:1:1 vol.%)	78% capacity retention after 350 cycles at 0.5C	2020	[39]
*x*PEG–GCS	Imine bond and H‐bond	Si anode	1 M LiPF_6_ in EC/EMC (1:2 vol.%) with 10% FEC	64.7% capacity retention after 150 cycles at 0.5C	2021	[349]
PET	H‐bond	Si anode	1 M LiFP_6_ in EC/DEC (1:1 vol.%) with 10 wt.% FEC	85.6% capacity retention after 250 cycles at 4.2 A g^−1^	2021	[350]
PAA–HPR	Boronic ester bond	Si anode	1 M LiPF_6_ in EC/DEC (1:1 vol.%) with 0.5 wt.% VC and 5% wt.% FEC	64.13% capacity retention after 250 cycles at 1 A g^−1^	2021	[351]
PAA–PEO	H‐bond	Si anode	1 M LiPF_6_ in EC/DEC/DMC (1:1:1 vol.%) with 10% FEC	2346 mAh g^−1^ after 100 cycles at 0.84 A g^−1^	2021	[209]
CB–GG	H‐bond	Si nanoparticle anode	1 M LiPF_6_ in FEC/EC/EMC (2:9:9 wt.%)	1200 mAh g^−1^ after 300 cycles at 1C	2021	[352]
GCS–OSA	Imine bond, dynamic covalent‐bond and H‐bond	Si anode	1M LiPF_6_ in EC/EMC (1:2 vol.%) with 10 wt.% FEC	68.4% capacity retention after 100 cycles at 0.2C	2021	[353]
CFG–PEG	H‐bond	Si anode	1M LiPF_6_ in DMC/EC (1:1 vol.%) with 10 wt.% FEC	1500 mAh g^−1^ after 200 cycles at 2 A g^−1^	2021	[129]
PEG–PAA	H‐bond	Si anode	1 M LiPF_6_ in EC/DEC (1:1 vol.%) with 10 vol.% FEC	1596 mAh g^−1^ after 800 cycles at 2 A g^−1^	2021	[141]
CA–PAA	H‐bond	Si anode	1 M LiPF_6_ in EC/DEC (1:1 vol.%) with 10 wt.% FEC	74% capacity retention after 100 cycles at 0.1C	2021	[354]
PVA	H‐bond	Si/carbon anode	1 M LiPF_6_ in EC/DEC/FEC (1:1:1 vol.%) with 10 vol.% FEC	73.7% capacity retention after 800 cycles at 1 A g^−1^	2022	[355]
Py–PAA/*γ*CDp	H‐bond	Si/graphite anode	1 M LiPF_6_ in EC/DEC (1:1 vol.%) with 10 wt.% FEC	86.4% capacity retention after 300 cycles at 0.5C	2022	[356]
PAA–PEG	H‐bond	Si anode	1 M LiFP_6_ in EC/DMC (1:1 vol.%) with 5 wt.% FEC	870 mAh g^−1^ after 200 cycles at 0.5C	2022	[357]
P‐BIAN/PAA	H‐bond	Si anode	1 M LiFP_6_ in EC/DEC (1:1 vol.%)	95% capacity retention after 600 cycles at 0.5 A g^−1^	2022	[358]
PAA–DA/PVA	Covalent bond, H‐bond, and dynamic bond	Si anode	1 M LiFP_6_ in EC/DEC (1:1 vol.%) with 5 vol.% FEC	73% capacity retention after 100 cycles at 0.2 A g^−1^	2022	[359]
GG–CA	H‐bond	Si anode	1 M LiPF_6_ in EC/DEC (1:1 vol.%) with 10% FEC and 1% VC	1184 mAh g^−1^ after 740 cycles at 2 A g^−1^	2022	[360]
AM/AA	H‐bond and covalent bond	SiO*_x_* anode	1 M LiPF_6_ in EC/DEC/EMC (1:1:1 vol.%) with 10 vol. % FEC	734 mAh g^−1^ after 300 cycles at 0.5C	2022	[156]
PR–PAA	*α*‐cyclodextrin	Si anode	1 M LiPF_6_ in EC/DEC (1:1 vol.%) with 5 wt.% FEC	82% capacity retention after 100 cycles at C/10	2023	[361]
GG–PAA–CA	H‐bond	Si anode	1 M LiPF_6_ in EC/DEC (1:1 vol.%) with 10% FEC and 2% VC	82.7% capacity retention after 200 cycles at 0.5 A g^−1^	2023	[362]
PAA–UPy/PEO	Gradient H‐bond	Si anode	1 M LiPF_6_ in EC/DEC (1:1 vol.%) with 5 wt.% FEC	1245 mAh g^−1^ after 200 cycles at 0.5C	2023	[339]
Al/Alg–TUEG	Dynamic coordination bond (Al–O) and H‐bond	Si anode	1 M LiPF_6_ in EC/EMC/DEC (1:1:1 vol.%)	77.4% capacity retention after 300 cycles at 0.5C	2023	[338]
PEDOT:PAA:PA	H‐bond	Si anode	1 M LiPF_6_ in EC/DEC (1:1 vol.%) with 15 wt.% FEC	74% capacity retention after 250 cycles at 0.5C	2023	[363]

PEG: polyethylene glycol; PVDF: polyvinylidene fluoride; SBR: 1,3‐butadiene polymer; PAA: poly(acrylic acid); UPy: ureido‐pyrimidinone; PR: polyrotaxane; Alg–C–chitosan: alginate–carboxymethyl chitosan; PVA: polyvinyl alcohol; CBA: 4‐carbonxybenzaldehyde; FPAA: furfurylamine‐functionalized poly(acrylic acid); BFPU: bifunctional polyurethane; CMC: carboxymethyl cellulose; CPAM: cationic polyacrylamides; GCS: glycol chitosan; PET: poly(ether‐thioureas); HPR: hydroxypropyl polyrotaxane; PEO: poly(ethylene oxide); CB: carbon black; GG: guar gum; OSA: oxidized alginate; CA: citric acid; AM: acrylamide; CFG: corn fiber gum; AA: acrylic acid; Py–PAA: pyrene‐conjugated PAA; *γ*CDp: *γ*‐cyclodextrin polymer; PEG: polyethylene glycol; P‐BIAN: poly(bisiminoacenaphthenequinone); DA: dopamine; PEDOT: poly (3, 4‐ethylenedioxythiophene); PA: phytic acid; PSS: poly (styrenesulfonate); PEO: polyethylene oxide; TUEG: poly(etherthioureas); Alg: alginate; H‐bond: Hydrogen bond; EC: ethylene carbonate; DEC: diethyl carbonate; FEC: fluoroethylene carbonate; VC: vinylene carbonate; FEC: 4‐fluoro‐1,3‐dioxolan‐2‐one; EMC: ethyl methyl carbonate; DMC: dimethyl carbonate; LiPF_6_: lithium hexafluorophosphate.

These self‐healing binder designs use 3D network conformation to ensure Si‐based anode integrity and sticky functional groups to improve affinity with Si‐based anode particles. While the binder preserves the necessary 3D network, it is still challenging to obtain the homogenous distribution of Si particles in the presence of a significant volumetric content of carbonaceous components (such as conductive agent, graphite, etc.).^[^ ^364^ ^]^ 3D and multifunction polymeric binders produced by chemical bonding, electrostatic interactions, and coordination interactions possess particularly high electrical conductivity, flexibility, and stickiness. The self‐healing binders are expected to accelerate the practical application of Si‐based anodes. In order to further development of bio‐inspired anodes, bio‐inspired binders have been got extensive attention.

#### 3.2.2 Bio‐Inspired Binders

**Figure 7:**
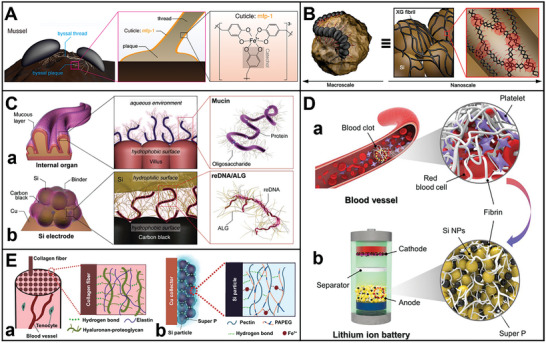
The bio‐inspired binders owning self‐healing capabilities: A) Mussel‐inspired binder.^[^ ^343^ ^]^ Copyright 2019, ACS Nano. B) Millipede‐inspired binder.^[^ ^366^ ^]^ Copyright 2015, Energy & Environmental Science. C) Mucin‐inspired binder. Conceptual illustration representing the a) analogy between mucin at biological interfaces and b) the DNA‐alginate hybrid binder at Si/carbon interfaces within anodes.^[^ ^367^ ^]^ Copyright 2018, Advanced Materials. D) Blood clot‐inspired binder. Schematics of a) blood clots (thrombus) in a blood vessel, b) Si anodes based on a fibrin binder.^[^ ^40^ ^]^ Copyright 2022, Energy Storage Materials. E) Sheath‐inspired binder. Schematic illustration of a) endotenon sheath in the tendon, b) bioinspired double‐network polymer binder design^[^ ^37^ ^]^ Copyright 2022, Nano‐Micro Letters.

Bio‐inspired binders have remarkable properties derived from natural materials with intrinsic adhesiveness or similar adhesive groups.^[^ ^24^ ^]^ Additives (binders and conductive additives) for bio‐inspired electrodes can be derived from biomass, showing promising results in LIB applications.^[^ ^185^ ^]^ Many biomass polymers and derivatives exhibit excellent mechanical robustness, facilitating lithium‐ion migration, protecting active materials, and capturing intermediates in the electrochemical process.^[^ ^365^ ^]^ Consequently, many cost‐effective and enviromentally friendly biomass‐based binders have been extensively used in LIB development. Furthermore, bio‐inspired binders with self‐healing capability mixed in anode preparation are exploited based on natural materials and creatures (**Figure** **7**), such as mussels,^[^ ^343^ ^]^ millipedes,^[^ ^366^ ^]^ mucins,^[^ ^367^ ^]^ blood clots,^[^ ^40^ ^]^ sheaths,^[^ ^37^ ^]^ plants,^[^ ^130^ ^]^ and spidroins.^[^ ^368^ ^]^ The binders possess both high tensile strength and elasticity, and strong electrode adhesion.^[^ ^368^ ^]^ They can maintain the integrity of conductive pathways, enhance their mechanical properties, and then improve their electrochemical performance.^[^ ^369^ ^]^ Thus, emerging bio‐inspired binders have improved the cycling lifetime of high‐capacity Si‐based bio‐inspired anodes.

Bio‐inspired electrodes can undergo modification using the catechol functional group or the mussel‐inspired binder. PDA's uniform and conformal coating capacity shields active materials from unfavorable electrolyte side reactions, significantly enhancing fundamental characteristics. Furthermore, the cycling lifetimes of newly developed high‐capacity anodes are considerably extended along with significant volume expansion owing to the wet adhesion resulting from catechol functional groups.^[^ ^370^ ^]^ Similarly, the capacities and cycle lifetimes of Si‐based anodes can be greatly enhanced by mussel‐inspired binders with exceptional wetness‐resistant adhesion capability while maintaining electrochemical stability.^[^ ^371^ ^]^ Drawing inspiration from the sticky byssus cuticle of mussels, a copolymer binder featuring Fe^3+^–(tris)catechol coordination cross‐links can induce a self‐healing effect for Si anodes (Figure 7A).^[^ ^343^ ^]^ The copolymer binder comprises monomer units with sufficient flexibility to improve interchain motions to efficiently restore Fe^3+^–(tris)catechol bonds. The high strength of the Fe^3+^–(tris)catechol coordination bond can repair the dissociated bond caused by the substantial volume expansion of Si. Consequently, using metallopolymers for metal–organic ligand coordination in interchain cross‐linking is promising for binders with self‐healing capabilities, ensuring sustainable cycling lifetimes for the high‐capacity anodes.

Further, newly applied polysaccharide binders, inspired millipede structures, are utilized to prepare high‐capacity Si anodes (Figure 7B).^[^ ^366^ ^]^ Similar to the strong adhesion observed in millipedes, which is based on adhesive pads on each leg, xanthan gum exhibits optimal structural performance by leveraging its double helical structure with side chains and ion‐dipole interactions.^[^ ^372^ ^]^ Drawing inspiration from mucin, an amphiphilic macromolecular lubricant secreted on the hydrophobic surface of the gastrointestinal interface aqueous serous fluid, a renatured DNA‐alginate binder for Si and Si–graphite blended anodes has been developed.^[^ ^367^ ^]^ The mucin‐inspired structure comprises a hydrophobic protein backbone and hydrophilic oligosaccharide branches. This binder facilitates the homogeneous distribution of anode materials and enhances adhesion to a current collector. Furthermore, by optimizing of the stiffness and stress relaxation through mixing fibrin with alginate followed by ionic cross‐linking, an advanced binder (blood clot‐inspired viscoelastic fibrin gel) with outstanding cycling stability was developed (Figure 7D).^[^ ^40^ ^]^ The mechanical and electrochemical performances of such anodes can be further enhanced by adding divalent cations during fibrinogen polymerization to promote covalent cross‐linking of fibrin or by controlling the fibrinogen concentration to adjust the mesh sizes of the fibrin network. Additionally, the endotenon sheath‑inspired binder, characterized by its excellent adhesive, mechanical properties, and self‐healing capacity mainly benefits from its reversible supramolecular hybrid network (Figure 7E).^[^ ^37^ ^]^ This binder manages the excessive volume expansion of silicon anodes during cycling with commendable mechanical strength, alleviates stress from silicon particle volume changes and achieves self‐healing capabilities. Moreover, the binder plays a role in forming SEI, contributing to anodes' reversible lithiation–delithiation behaviors. Consequently, the bio‐inspired network can extend its applicability to additional classes of anode materials with polarity on their surfaces, particularly those experiencing significant volume variations. Si anodes containing bio‐inspired binders exhibit superior adhesion, rate capability, charge capacity, and cycling performance while effectively accommodating the volume expansion of Si particles.

In summary, the binder is critical in the functionality of Si‐based anodes. Understanding the interconnected dynamics among electrochemistry, anode adhesion, and binder mechanics is critical for advancing state‐of‐the‐art binder design. Polymers integrating supramolecular functionalities offer promising avenues for binder development because strong supramolecular interactions can promote self‐healing effects by recovering cleaved crosslinks. Bio‐inspired binders, characterized by exceptional wetness‐resistant adhesion, can enhance the rate capacity, and cycling lifetime of Si‐based anodes while maintaining electrochemical stability under operational potentials. This self‐healing capability protects emerging high‐capacity active materials against substantial volume changes. The design of bio‐inspired binder design provides a promising strategy to prolong the cycling lifetime of bio‐inspired anodes, presenting significant potential for enhancing the electrochemical performance of LIBs.

### 3.3 Self‐Healing Liquid Metal Anodes

The alloy‐type anode, existing in a liquid state, is one of the most promising options for self‐healing electrodes, primarily owing to its exceptional fluidity and surface tension,^[^ ^32^, ^42^ ^]^ characteristics crucial for extending the cycling lifetime of LIBs. The volume expansion/contraction inherent in high‐capacity anodes like Si‐based and Sn‐based electrodes can lead to structural and mechanical fractures, resulting in subpar cycling performance.^[^ ^44^ ^]^ Room‐temperature liquid metal (LM) anodes leverage reversible solid–liquid phase transitions to attain self‐healing capabilities and deter the lithium dendrite formation during cycling.^[^ ^43^ ^]^ As the LM anode crystallizes during lithiation and transitions to a solid state, it can revert to a liquid state during delithiation. Consequently, cracks formed in the anode, particularly during delithiation, can self heal through solid‐to‐liquid transformation. The proposed approach of LM anodes, feasible with various low melting point alloys like liquid gallium (Ga), is promising for application in LIBs.^[^ ^122^ ^]^ Emerging Ga‐based LMs for anode materials encompass gallium–indium alloy (EGaIn),^[^ ^51^, ^163^ ^]^ gallium–tin alloy (EGaSn),^[^ ^43^, ^44^, ^45^, ^373^ ^]^ and gallium–indium–tin alloy (EGaInSn),^[^ ^41^, ^374^ ^]^ each offering self‐healing capabilities facilitated by Ga generated during the conversion reaction.^[^ ^331^ ^]^

**Figure 8:**
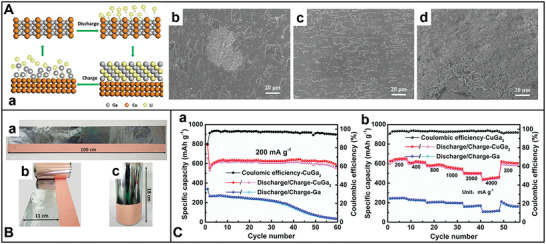
A) a) Schematic illustration illustrating the self‐healing process of the CuGa2 anode. The SEM images of CuGa_2_ films b) at the as‐prepared state, c) at full delithiation after the first cycle, and d) at full delithiation after 100 cycles. B) Photographs of CuGa_2_ film, which illustrate its scalability and flexibility. C) (a) Galvanostatic cycling performance of CuGa_2_ and Ga anode at 0.2 A g^−1^. b) The rate capability of the CuGa_2_ anode and Ga anode at different current densities.^[^ ^46^ ^]^ Copyright 2019, Journal of Power Sources.

The search for new anode materials with inherent self‐healing capabilities has become increasingly urgent to meet growing demands.^[^ ^375^ ^]^ Flexible, self‐supporting CuGa_2_ films are easily fabricated by applying liquid Ga onto commercial Cu foils, which can serve as anodes directly after annealing (**Figure** **8**A,B).^[^ ^46^ ^]^ The alloying–dealloying processes (CuGa_2_ + 2*x*Li^+^ + 2*x*e^−^ ↔ Cu + 2Li*_x_* Ga) are validated during the first and fourth cycles. Upon application of CuGa_2_ films, these anodes exhibit a higher capacity exceeding 630 mAh g^−1^ at 0.2 A g^−1^ and demonstrate improved rate capability, reaching 463.7 mAh g^−1^ at 4 A g^−1^ compared to pure Ga anodes (Figure 8C).^[^ ^46^ ^]^

The Ga‐based alloy achieves self‐healing capability through Ga's liquid–solid–liquid transition during lithiation–delithiation cycling processes. A novel self‐healing anode with ultra‐high capacity and cycling stability is synthesized by incorporating semiconducting metal phosphide with Ga into P.^[^ ^121^ ^]^ Additionally, incorporating a TiO_2_–C hybrid matrix enhances the mechanical integrity and electrical conductivity of the GaP alloy.^[^ ^123^ ^]^ TiO_2_‐based materials offer unique properties such as rapid lithium‐ion diffusion, affordability, enviromental friendliness, and safety. Consequently, the resulting anode exhibits outstanding rate capability and superb cycling performance, reaching 1012 mAh g^−1^ at 0.5 A g^−1^ after 500 cycles. A 3D free‐standing anode is fabricated by confining Ga‐based LM (EGaIn) within a matrix of carbon nanofibers and carbon nanotubes.^[^ ^163^ ^]^ The carbon nanotubes maintain complete conductive networks of the electrodes throughout cycling, ensuring a uniform lithiation reaction.^[^ ^111^, ^144^ ^]^ A dense carbon nanotube layer prevents EGaIn from detaching from the conductive substrates. This network structure provides effective pathways for electrons and ions and sufficient space to accommodate the volume expansion of EGaIn. As a result, the 3D free‐standing anode exhibits excellent ionic and electronic conductivity and mechanical stability.

The room‐temperature LM composed of Ga and Sn, known as EGaSn, is a novel self‐healing material with promising applications as an anode in LIBs. This LM anode exnibits intrinsic self‐healing properties at room temperature owing to its liquid nature. Given the high theoretical capacities of both Ga and Sn (769 and 990 mAh g^−1^, respectively), the EGaSn LM anode demonstrates remarkable performance, delivering high capacities of 775, 690, and 613 mAh g^−1^ at 0.2, 0.5, and 1 A g^−1^, respectively.^[^ ^59^ ^]^ Moreover, it exhibits outstanding cycling stability, maintaining a specific capacity of 400 mAh g^−1^ after over 4000 cycles at 4 A g^−1^, surpassing other metal anodes.^[^ ^44^ ^]^ Additionally, EGaSn serves as a liquid buffer for Si anodes during cycling, enabling the repair of cracks resulting from volume changes.^[^ ^373^ ^]^ With a reversible capacity of 670 mAh g^−1^ after 1000 cycles, the EGaSn anode demostrates excellent cycling stability and exceptional rate capability. Furthermore, novel EGaInSn nanoparticles exhibit a specific capacity of 474 mAh g^−1^ and maintain a reversible capacity of 77% after 500 cycles at 0.1 A g^−1^.^[^ ^41^ ^]^ Leveraging its high theoretical specific capacity, self‐healing capability, and fluidic properties, EGaInSn is a promising candidate for large‐scale energy storage applications. The disparate equilibrium lithiation–delithiation potentials between Si and Ge also mitigate instantaneous mechanical stress.^[^ ^42^ ^]^ By incorporating EGaInSn into nanoporous Si–Ge alloy, the modified anode effectively accommodates expansion and repairs surface damage. LM particles are successfully integrated within the porous structures on the anode surface, resulting in impressive performance. This anode achieves a capacity of 1200 mAh g^−1^ after 300 cycles at standard operating temperatures, while maintaining a capacity of 746.7 mAh g^−1^ after 100 cycles at −20 °C.^[^ ^42^ ^]^ Notably, the self‐healing LM anode exhibits highly reversible Li storage performance under normal and low‐temperature conditions.

In summary, the volumetric changes during lithiation–delithiation cycling induce structural cracks in conventional anodes, leading to decreased rate capability and cycling stability. However, Ga‐based room‐temperature LM exhibits remarkable electrochemical performance owing to its inherent self‐healing capability and fluidic characteristics. Consequently, LM is a promising candidate for large‐scale LIB applications. The design of LM anodes with self‐healing capabilities is a viable pathway for enhancing the cycling lifetime of bio‐inspired LIBs.

### 3.4 Self‐Healing Microcapsules

**Figure 9:**
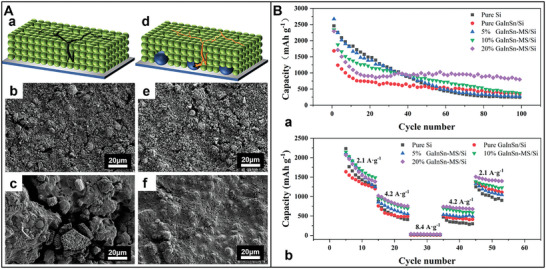
A) Schematic illustration of a) the pure Si anode and d) the EGaInSn–MS/Si hybrid anode. SEM images of b,c) the pure Si anode and e,f) 20% EGaInSn–MS/Si hybrid anode after 100 cycles. B) Cycling performance of the EGaInSn–MS/Si anode, pure EGaInSn/Si anode, and pure Si anode at 2.1 A g^−1^ a) and rate capability of the EGaInSn–MS/Si hybrid anode, pure EGaInSn/Si anode, and pure Si anode at 2.1, 4.2, and 8.4 A g^−1^ b).^[^ ^48^ ^]^ Copyright 2022, ACS Applied Energy Materials.

Engineering bio‐inspired anodes for LIBs is of great significance, and it can solve the restricted application of Si by their vast volumetric expansion and poor conductivity. The fluidity and metallic conductivity of gallium‐based alloys,^[^ ^374^ ^]^ such as gallium–indium–tin alloy (EGaInSn),^[^ ^41^ ^]^ and gallium–tin alloy (EGaSn),^[^ ^50^ ^]^ provide a possibility for the development of bio‐inspired electrodes containing self‐healing microcapsule. For instance, a self‐healing EGaInSn–MS/Si hybrid anode is generated applying an additive of EGaInSn possessing high electrical conductivity and mobility (**Figure** **9**A).^[^ ^48^ ^]^ EGaInSn is encapsulated into microcapsules via poly(urea‐formaldehyde) (PUF). When an LM microcapsule is included in the hybrid anode, the dispersed EGaInSn can both prevent oxidation into a solid‐state gallium oxide with reduced conductivity and facilitate fast fluidity and uniform dispersion. Therefore, when the bio‐inspired anode develops fractures, the built‐in self‐healing hybrid anode does not require manual splicing, accomplishing a spontaneous self‐healing capability. Furthermore, such anode possesses excellent structural integrity and cycling stability, and thus retains a capacity of 806.7 mAh g^−1^ after 100 cycles at 2.1 A g^−1^ (Figure 9B).

Further, a self‐healing anode, EGaSn encapsulated within microcapsules is prepared through an oil‐in‐water emulsion method. The liquid nature of EGaSn within the microcapsules enables a self‐healing mechanism to mitigate structural failures during prolonged cycling.^[^ ^50^ ^]^ The presence of voids in the microcapsules helps alleviate the volumetric changes of EGaSn during cycling, while the carbon shell enhances conductivity. Additionally, the self‐healing microcapsules exhibit a recoverable rate capability. Furthermore, the stable PPy layer acts as a protective “armor” on the LM's surface.^[^ ^374^ ^]^ Leveraging the properties of PPy, PAA is incorporated into the shell materials to reinforce the anode's integrity through hydrogen bonding. This “dual‐insurance” design enhances the self‐healing efficacy of EGaSn while improving electrochemical kinetics.^[^ ^45^ ^]^ A simple method involves synthesizing LM nanoparticles through carbon‐encapsulated EGaSn.^[^ ^43^ ^]^ Core–shell fibers with self‐healing EGaSn nanoparticles as the core and a carbon shell are produced using a straightforward coaxial electrospinning and subsequent carbonization process. These fibers, containing nanosized self‐healing LM particles within the shell's precisely engineered inner void space, serve as free‐standing anodes for LIBs. The resulting anodes exhibit outstanding rate capability and remarkably stable cycling performance, retaining a discharge capacity of 552 mAh g^−1^ after 1500 cycles at 1 A g^−1^.^[^ ^376^ ^]^ Thus, these customized bio‐inspired anodes demonstrate exceptional rate capability and cycling stability.

In summary, integrating LM microcapsules into the self‐healing Si anode structure offers a straightforward synthesis pathway for developing bio‐inspired anodes. Incorporating self‐healing microcapsules ensures the anode's self‐healing capability and high‐performance. These promising strategies present significant opportunities for creating cutting‐edge self‐healing anode materials. The LM microcapsules' self‐healing ability greatly enhances the bio‐inspired anodes' cycling performance. The unique core–shell structure, featuring a well‐designed void space, effectively mitigates volume changes in LM nanoparticles during lithiation–delithiation cycles. Furthermore, the self‐healing LM nanoparticles, comprising Ga and Sn, exhibit high capacities. These attributes position the self‐healing Ga‐based alloy‐encapsulated microcapsules as promising for practical applications.

### 3.5 Self‐Healing Current Collectors

**Figure 10:**
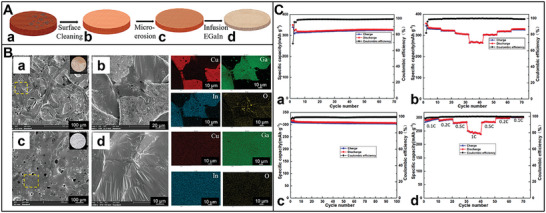
A) Schematic illustration of the fabrication process of the EGaIn@3D‐Cu current collector. B) SEM and EDS images of the EGaIn embedded 3D‐Cu foam with controllable loading mass. The mass loading of EGaIn is a,b) 0.0027 g, c,d) 0.005 g. C) Cycling and rate capabilities of the self‐healing graphite anode a,b) without and c,d) with scratch damage at 0.2C.^[^ ^51^ ^]^ Copyright 2021, Electrochemistry Communications.

Current collectors are obbligato components that provide electron transport and mechanical support of electrode materials in a LIB.^[^ ^377^, ^378^ ^]^ However, the low electronic conductivity and exfoliation from the Cu current collector contribute to poor rate capability and irreversible cycling performance. Herein, the emerging current collector can improve the interface capability and energy density and prolong the service life.^[^ ^379^ ^]^ A new self‐healing EGaIn@3D‐Cu current collector generates by introducing EGaIn into the micro‐etched 3D‐Cu foam (**Figure** **10**A).^[^ ^51^ ^]^ And the composite current collectors possessing excellent self‐healing capability and electric conductivity can be obtained at heating at 160 °C for 4 h (Figure 10B). In addition, the graphite anode possessing self‐healing capability exhibits exceptional capacity retention of 94.2%. The specific capacity of the graphite anode remains high, with values of ≈327.4 mAh g^−1^ before scratch damage and ≈309.6 mAh g^−1^ after damage (Figure 10C).^[^ ^51^ ^]^ Such an anode equipped with a self‐healing current collector is better than a commercial graphite anode coated on the 2D‐Cu foil. Thus, the self‐healing current collector can achieve self‐healing capability when the bio‐inspired anode is damaged.

In addition, siloxene, characterized by its 2D structure, exhibits unique properties such as small volume change and high theoretical capacity. A robust room‐temperature LM matrix, integrated with the siloxene structure, is achieved through heat treatment at 80 °C,^[^ ^380^ ^]^ facilitating the formation of metal bonds. This process allows LM to permeate the siloxene anode, enhancing conductivity and reacting with the Cu current collector to form a CuGa_2_ alloy. Leveraging the combined benefits of high electrical conductivity, environmental sustainability, and the self‐healing capability of LM, the modified siloxene anode demonstrates improved cycling stability.

In summary, the novel current collector exhibits self‐healing capabilities, effectively mitigating volume expansion, perforation damage, and mechanical stresses in LIBs when the electrode structure is compromised. Additionally, its electrochemical contacts with the active materials and the current collector are significantly reinforced through robust metal bonds, minimizing the exfoliation of electrode materials. With its superior self‐healing capability, this emerging current collector is promising as a key component for large‐scale LIB applications.

## 4 Fast Ion Transport Electrodes

Fast ion transport electrodes are developed to enhance the electrochemical performance of LIBs.^[^ ^381^ ^]^ Subtle structure variations lead to a different storage mechanism and control how ions are stored.^[^ ^109^ ^]^ Emerging electrode architectures can enhance the electrochemical performance of LIBs, enhancing attributes like energy density, power density, and lifetime.^[^ ^382^ ^]^ Microstructure design integrates the high activity of nanostructures and the thermal stability of microstructures.^[^ ^158^ ^]^ Drawing inspiration directly from nature, bio‐inspired structures offer many unique properties, including outstanding excellent mechanical robustness, large surface area, numerous active sites, and ion channels.^[^ ^383^ ^]^ In order to enhance the cycling lifetime of state‐of‐the‐art batteries, many bio‐inspired structured electrodes have been designed and prepared,^[^ ^384^ ^]^ such as human eye‐inspired structures for Li–O_2_ battery,^[^ ^385^ ^]^ pomegranate‐structured cathode for Li–S battery,^[^ ^386^, ^387^ ^]^ leaf‐inspired electrode,^[^ ^388^ ^]^ seeds‐inspired air cathode for Li–CO_2_ battery,^[^ ^389^ ^]^ bio‐inspired multiscale‐pore‐network structured carbon electrode,^[^ ^390^ ^]^ tree‐root‐inspired interfacial structured cathode for Zn–Ag_2_O battery,^[^ ^391^ ^]^ and plant roots‐inspired structured cathode for Li–S battery.^[^ ^392^ ^]^

### 4.1 Vertical Microchannel Structured Thick‐Film Cathodes

**Figure 11:**
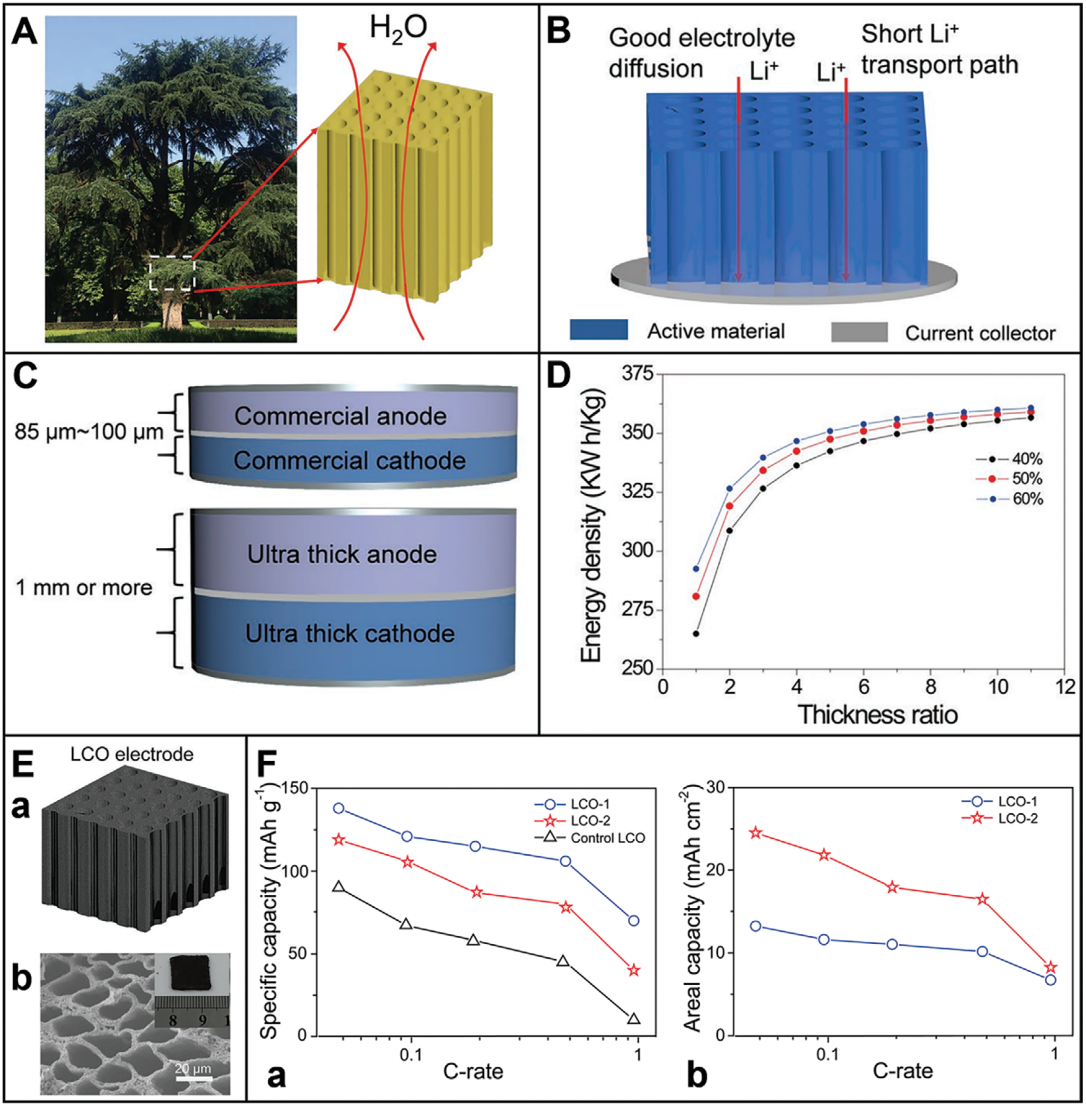
Wood‐inspired design for the ultrathick electrodes. A) The photograph of pinewood and its illustration of the wood anisotropic structure for the transport of water. B) The illustration of wood‐inspired cathode to shorten the lithium‐ion transport path. C) The model illustration for the comparison of commercial electrode and ultrathick electrode. D) The plot of the trends of the increase of energy density of LCO‐graphite cell with the increase of the thickness of electrodes at different porosities. E) The fabrication and characterization of ultrathick bulk LCO cathodes by wood templating. a) The ultrathick LCO cathode by wood templating. b) The top view of the LCO‐1 electrode. F) a) The plot of specific capacity versus discharge rate for LCO‐1, LCO‐2, and control LCO cathodes. b) The plot of areal capacity versus C‐rate for LCO‐1 and LCO‐2.^[^ ^62^ ^]^ Copyright 2018, Advanced Materials.

Bio‐inspired organic compounds and their potential structural diversity will aid in advancing the development of cathode materials for high‐performance LIBs.^[^ ^393^, ^394^ ^]^ In nature, the vertical microchannels in wood are the channels for water transport (**Figure** **11**A).^[^ ^62^ ^]^ These uniform microchannels permeate the entire wood‐templated cathode. The thick cathode combines a carbonaceous scaffold with a hierarchical structure and aligned channels. This wood‐inspired cathode effectively reduces the lithium‐ion transport distance, enhancing ion and electron conductivities and charge transfer kinetics (Figure 11B).^[^ ^202^ ^]^ The resulting LCO cathode has a thickness of up to 1 mm, ≈12 times thicker than conventional commercial cathodes (Figure 11C). The specific energy density of such a LIB is accordingly greatly increased when LCO cathode thickness ratio increases (Figure 11D). Based on vertical microchannels structure of wood, the bio‐inspired micro‐structures are designed into the ultrathick bulk LCO cathode (Figure 11E). The wood‐inspired LCO cathode possesses a high areal capacity of up to 22.7 mAh cm^−2^ and excellent rate capability (Figure 11F). It brings in ≈2 times higher lithium‐ion conductivity and 1.5 times lower tortuosity compared to normal LCO cathode. Porous structures offer significant potential to enhance the lithium‐storage performance of electrode materials in terms of cycling stability, specific capacity, and rate capability.^[^ ^395^ ^]^ The stress accumulation in the porous structured electrode can be effectively alleviated, which further ensures the enhancement of mechanical stability.^[^ ^13^ ^]^ Thus, bio‐inspired structured cathodes are the best candidate components for high‐energy LIBs.

Lithium‐ions are shuttled between the cathode and anode through the electrolyte, and the electrons are transferred through the external circuit during charge–discharge processes. For the solid‐state diffusion of Li in the electrode, the mean diffusion (or storage) duration is governed by the diffusion coefficient and the diffusion length. The characteristic diffusion time constant is given by the following formula:^[^ ^396^ ^]^

(3) $$\tau_{\mathrm{eq}}=L^{2}\;/2D$$

where *τ*_eq_, *L* and *D* represent the mean diffusion time, diffusion length and diffusion coefficient, respectively.

To enhance the kinetics of ions in the bio‐inspired electrodes, there are two approaches: One approach is increasing *D* by doping the electrode materials with foreign atoms. This leads to enhanced mixed conduction, however only limited rate‐performance augmentation is possible, and heteroatom insertion occasionally causes unstable crystal structure;^[^ ^397^ ^]^ Another approach involves reducing *L*, achievable through modifications to the nanostructures of the electrode materials. For example, higher rate capability can be attained by shortening transport distances for electrons and lithium ions, along with enhancing the electrode/electrolyte contact. Thus, applying the various nanostructures on the electrodes is an effective mean to achieve the superior electrochemical performance of LIBs.

### 4.2 Various Bio‐inspired Structured Electrodes

The development process employing biological fabrication is featured with low cost and can have mass production.^[^ ^398^ ^]^ The performance of various bio‐inspired electrodes with bio‐inspired structures is compared in **Table** **3**, and most of these structures are applied to anodes to enhance their electrochemical performances. These structures effectively reduce the diffusion distance of lithium‐ions in electrode pores filled with electrolytes by linking these pores with the free electrolyte.^[^ ^63^ ^]^ Consequently, the electrochemical performance of LIBs can be significantly enhanced. LIBs with bio‐inspired smart electrodes demonstrate notably higher capacity retention, reaching 93.8% after 1000 cycles at 10 C. In contrast to conventional electrodes, channels within the bio‐inspired structures improve their lithium‐ion diffusion kinetics. The natural porous structures are often associated with the high surface areas.^[^ ^399^ ^]^ Thus, the hollow cavity can provide free space for strain relaxation and volume‐change accommodation of the electrode materials during the lithiation–delithiation cycling process. As a consequence, the pulverization of electrode materials will be alleviated and hence the electrochemical performance achieves significant improvement.

**Table 3:** Factors involved in the bio‐inspired structures on bio‐inspired electrodes for LIBs.

Prototype	Electrode	Electrolyte	Performance	Structure	Year	Reference
Wood	LCO cathode	1 M LiPF_6_ in EC/DMC/EMC (1:1:1 wt.%)	> 70% capacity retention at 0.5C	Vertical microchannel structure	2018	[62]
Corn	NMC 811 cathode	1 M LiPF_6_ in EC/ EMC (1:1 vol.%)	85.71% capacity retention after 100 cycles at 0.2 A g^−1^	Hollow structure	2018	[400]
Crab shell	Si anode	1 M LiPF_6_ in EC/DEC (1:1 vol.%)	95% capacity retention after 200 cycles at C/5	Bouligand structure	2013	[65]
Silk	Nitrogen‐doped carbon nanosheets anode	1 M LiPF_6_ in EC/DMC	9% loss after 10000 cycles	Hierarchical porous structure	2015	[401]
Custard‐apple	Si anode	1 M LiPF_6_ in EC/DMC/DEC (3:4:3 vol.%)	1401 mAh g^−1^ after 500 cycles at 1 A g^−1^	Hierarchical porous structure	2017	[60]
Bacteria	Fe_3_O_4_–carbon/graphene anode	1 M LiPF_6_ in EC/DMC (1:1 vol.%)	1112 mAh g^−1^ after 200 cycles at 0.1 A g^−1^	Hierarchical structure	2017	[398]
Cage	Si/carbon anode	1 M LiPF_6_ in EC/DEC/EMC (1:1:1 vol.%) with 2 vol.% VC	86.94% capacity retention after 60 cycles at 0.2 A g^−1^	Porous and conductive framework structure	2018	[402]
Cheese	Carbon anode	1 M NaClO_4_ in EC/DEC (1:1 vol.%)	92.8% capacity retention after 80 cycles at 0.1 A g^−1^	Multi‐channel 3D porous structure	2019	[61]
Fungus	Carbon anode	1 M LiPF_6_ in EC/EMC/DMC (1:1:1 vol.%)	93.8% capacity retention after 1000 cycles at 10C	Hierarchically porous structure	2020	[403]
Jujube cake	SiO_2_ anode	1 M LiPF_6_ in EC/DEC (1:1 vol.%)	87% capacity retention after 100 cycles at 0.1 A g^−1^	Porous structure	2021	[150]
Nanofibrous	SnS/carbon anode	1 M LiPF_6_ in EC/DEC (1:1 vol.%)	612 mAh g^−1^ after 70 cycles at 1 A g^−1^	Hierarchical 3D porous network nanostructure	2021	[404]
Coral	Si anode	1 M LiPF_6_ in EC/DMC (1:1 vol.%)	72% capacity retention after 100 cycles at 0.25 A g^−1^	Porous structure	2021	[405]
Water lily seed	Si/carbon anode	1 M LiPF_6_ in EC/DEC (1:1 vol.%) with 5 wt.% VC	1235 mAh g^−1^ after 400 cycles at 0.5C	Compact structure	2022	[406]
Biomineralization	CuO/Fe_2_O_3_anode	1 M LiPF_6_ in EC/DEC/DMC (1:1:1 vol.%)	1130 mAh g^−1^ after 100 cycles at 0.1 A g^−1^	Hybrid structure nanosheets	2022	[407]
Ferroconcrete	Si anode	1 M LiPF_6_ in EC/DEC (1:1 vol.%) with 5 vol.% FEC	79% capacity retention after 200 cycles at 1.5 mA cm^−2^	Multiple layer structure	2023	[110]
Rambutan	Si oxycarbide anode	1 M LiPF_6_ in EMC/EC/DEC (1:1:1 vol.%)	98% capacity retention after 600 cycles at 1.0 A g^−1^	Hollow structure	2023	[408]

Then the bio‐inspired structured anodes will be discussed in the following. The bio‐inspired electrode exhibits a limiting current density that is 83% higher compared to random porous‐structured electrodes.^[^ ^409^ ^]^ One example is the pomegranate‐inspired structure, characterized by intriguing micro‐flower morphology enclosing core–shell binary Ni sulfide nanobeads, which effectively addresses poor cycling performance in energy storage devices.^[^ ^410^ ^]^ In this structure, pomegranate‐inspired architectures on Si nanoparticles are enveloped by a conductive carbon layer, creating ample space for expansion and contraction during lithiation–delithiation cycles. This hierarchical arrangement ensures the stability and spatial confinement of the SEI, leading to exceptional cycling stability with a capacity of 724.1 mAh g^−1^ after 1225 cycles at 1C. Furthermore, the micro‐structures reduce the electrode–electrolyte contact area, resulting in high Coulombic efficiency of 99.87% and volumetric capacity of 1270 mAh cm^−2^ while maintaining stable cycling even at areal capacities comparable to current commercial LIBs (3.7 mAh cm^−2^).^[^ ^58^ ^]^ Subsequently, multilayer vertically aligned carbon nanotubes supported by a Si film with ferroconcrete‐inspired structures are developed using a layer‐by‐layer technique to enhance both areal and volumetric capacities. These frameworks facilitate electron transport and stabilize the anode structure during cycling, with the Si cladding layer providing a high capacity. A 3‐layer ferroconcrete‐inspired Si anode film exhibits superior areal/volumetric capacity and remains 79% of its capacity after 200 cycles.^[^ ^110^ ^]^ The layer‐by‐layer manufacturing approach enables the design of ferroconcrete‐inspired film structures, offering insights into developing high‐performance electrodes with high areal and volumetric capacities for post‐LIBs with increased energy density.

A porous, Si anode inspired by coral exhibits a remarkable reversible capacity of 2451 mAh g^−1^, representing ≈70% of the theoretical capacity of Si at C/10.^[^ ^411^ ^]^ This anode sustains a capacity of 1956 mAh g^−1^, equivalent to 79.8% of the initial reversible capacity, even after 100 cycles. Furthermore, a coral‐inspired porous Si/carbon material owning hierarchical pores and core–shell structure makes such anode possess a stable cycling performance with a reversible capacity of 990.6 mAh g^−1^ after 100 cycles at 0.25 A g^−1^.^[^ ^405^ ^]^ A water lily seed‐inspired Si/carbon composite anode is prepared via the polymer (PMMA) coating approach, and thus such anodes have their own void space. The multiple Si particles are polymer‐coated rather than individual Si particles to achieve a more compact structure.^[^ ^406^ ^]^ The as‐prepared Si/carbon composite anode possesses a high Coulombic efficiency and high reversible capacity after 400 cycles. Additionally, the cage‐inspired porous carbon microspheres can assure strong electrical contact and quick transit for electrons and ions, as well as accommodating the volume expansion of the Si anode.^[^ ^402^ ^]^ The composite microspheres contain Si particles embedded within a porous carbon framework comprising interwoven carbon nanotubes, filled carbon blacks, and interconnected amorphous carbon derived from polymers. These Si‐embedded porous carbon microspheres provide an outstanding conductive framework for ions and electrons. Consequently, the bio‐inspired Si‐based and Si/carbon composite anodes exhibit high capacity and superior rate capability.

For the other anode materials, a novel 3D Jujube cake‐inspired porous SiO_2_@pourous carbon@Sn composite anode material can be synthesized. SiO_2_ microparticles are encapsulated within porous carbon, while Sn nanoballs are uniformly dispersed in SiO_2_@porous carbon structures resembling sesame seeds. This structure forms a robust and conductive 3D porous architecture inspired by Jujube cake, facilitating fast ion transfer and ensuring high structural stability.^[^ ^150^ ^]^ A bio‐inspired nanofibrous SnS/carbon composite, utilizing natural cellulose substance as the structural scaffold and carbon source, is also synthesized, exhibiting superior performance.^[^ ^404^ ^]^ The resulting nanocomposite material features a unique 3D porous structure with micro‐to‐nano morphological characteristics obtained from the initial cellulose substance. The hierarchical porous network structure of the nanofibrous carbon conductive matrix, coated with an ultrathin carbon layer and immobilized with SnS nanoflakes, enhances electrode–electrolyte contact, accommodates substantial volume variations of SnS, prevents active particle aggregation and facilitates electron transfer and lithium‐ion diffusion during cycling processes. Furthermore, a bacteria‐inspired, micro‐/nanostructured Fe_3_O_4_–carbon/graphene foam hybrid materials are fabricated as anodes.^[^ ^398^ ^]^ Such anode possesses an excellent rate capability and high reversible capacity of 1112 mAh g^−1^ after 200 cycles at 0.1 A g^−1^. Another anode, inspired by the biomineralization mechanism, involves synthesizing CuO/Fe_2_O_3_ hybrid ultrathin nanosheets. Polyvinylpyrrolidone‐decorated CuO nanosheets are used as growth modifiers to control the hydrolysis process of Fe^2+^. The abundant 2D/2D interfaces generated through this bio‐inspired synthesis method effectively mitigate self‐stacking phenomena during cycling, thus ensuring high operational stability.^[^ ^407^ ^]^ Another outstanding anode material features a 3D fungus‐structured carbon combined with CuC_2_O_4_·*x*H_2_O nanocrystals.^[^ ^403^ ^]^ The hierarchically porous carbon provides an ideal structure for fast electron and ion transport. At the same time, the unique redox properties of CuC_2_O_4_·*x*H_2_O nanocrystals suppess SEI formation, resulting in exceptional rate capability and cycling stability.

### 4.3 Biomass Carbon Anodes

On the other hand, harnessing cheap and sustainable bio‐materials as raw precursors to prepare valuable carbon anodes can alleviate the dependence on nonrenewable resources and bring great benefits to LIB to some degree.^[^ ^67^, ^412^ ^]^ The hollow nanofiber anodes are constructed by crab shells with the Bouligand structure consisting of highly mineralized chitin–protein fibers. Then these fibers can be applied to encapsulate Si to form anodes. Such anodes possess high specific capacities of 3060 mAh g^−1^ for Si and excellent cycling performance up to 200 cycles with 95% capacity retention.^[^ ^65^ ^]^ It is feasible to scale up bio‐inspired organic materials as electrode materials in LIBs.^[^ ^384^ ^]^ By integrating biomass carbons with Si and optimizing the properties of resulting composites like Si/carbon or SiO*_x_*/carbon, the energy density and cost‐effectiveness of LIBs can be significantly enhanced. Optimized Si‐based composite anodes, when paired with Ni‐rich cathodes decorated with functionalized bio‐inspired surface layers are promising for achieving high energy density.^[^ ^66^ ^]^ Inspired by cheese‐like structures and abundant heteroatoms, carbon anodes establish efficient ion–electron transport channels, enhance conductivity, and introduce numerous active sites. Owing to this well‐designed configuration, such an anode exhibits exceptional capacity retention of 104.8% and commendable rate capability of 567.5 mAh g^−1^ after 80 cycles at 0.1 A g^−1^.^[^ ^61^ ^]^

Further, hierarchical porous nitrogen‐doped carbon nanosheets have been synthesized by activating and graphitizing biomass‐derived natural silk.^[^ ^401^ ^]^ These nanosheets possess advantageous characteristics for electrochemical energy storage, including a high specific surface area of 2494 m^2^ g^−1^, a substantial volume of hierarchical pores measuring 2.28 cm^3^ g^−1^, nanosheet morphology, and a significant nitrogen‐doping level of 4.7%. Benefiting from the synergistic effects of these features, the anode demonstrates a remarkable reversible lithium storage capacity of 1865 mAh g^−1^, representing the highest performance among nitrogen‐doped carbon anode materials to date. Additionally, a custard‐apple‐inspired Si@nitrogen, O‐dual‐doped carbon hierarchical porous structure exhibits exceptional reversible capacity at high current density, couple with outstanding rate capability and a long cycling lifetime of over 4000 cycles as an anode for LIBs.^[^ ^60^ ^]^ The 3D free‐standing anodes, consisting of FeCo_2_O_4_ nanocluster arrays on lotus leaf substrates, exhibit remarkable performance, maintaining a stable Coulombic efficiency of 99.9% and a high areal capacity of 2.4 mAh cm^−2^ after 100 cycles.^[^ ^64^ ^]^ Even under extreme conditions, such as long‐term cycling at −10 °C, the capacity remains at 1.15 mAh cm^−2^ after 900 cycles, and at a high temperature of 45 °C, it remains at 1.95 mAh cm^−2^ after 150 cycles. The anode demonstrates recoverable rate capability under various conditions, highlighting its potential for practical applications. Furthermore, this fabrication approach has been successfully applied to produce numerous other composites, including NiCo_2_O_4_, ZnCo_2_O_4_, CuCo_2_O_4_ nanocluster arrays on lotus leaves, as well as NiCo_2_O_4_, ZnCo_2_O_4_, CuCo_2_O_4_, FeCo_2_O_4_ nanocluster arrays on bamboo leaves, showcasing its excellent applicability.

In summary, the bio‐inspired strategy will open a new avenue to adopt natural hierarchical structured electrodes to enhance the electrochemical performance of LIBs. The bio‐inspired structural design is a promising appoach for fabricating electrodes with exceptionally high areal and volumetric capacities. The large surface area and volume accommodate more ions, while active sites facilitate intermediate conversion. The interconnected macropores and mesoporous channels derived from a bio‐inspired 3D porous structure can accelerate the ion migration and promote electrolyte impregnation, and then greatly promote charge–discharge processes. Biomass material can be used as a low‐cost and sustainable nano template and is considered an exciting direction for nanostructured LIB materials. The strategy of bionic material synthesis combined with the self‐assembly method is used to improve the large volume variation of anode materials during cycling processes. The bio‐inspired smart electrodes have great potential to meet the challenges arising from the application of Si nanoparticles as anode for next‐generation large‐scale LIB.

## 5 Flexible Deformation Electrodes

FLIBs are the critical power components for wearable and flexible electronic devices due to their bent, folded, and stretched deformations.^[^ ^68^, ^182^ ^]^ Their development of high‐performance requires interdisciplinary efforts including materials, electrochemistry, and mechanics.^[^ ^289^ ^]^ Unlike conventional rechargeable LIBs, FLIBs are essential for bendable and biocompatible characteristics. Because learning from nature remains a widely employed strategy for discovering new inspirations,^[^ ^68^ ^]^ various approaches exist in biology for creating flexible structures from rigid segments.^[^ ^184^ ^]^ Highly evolved natural organisms are primary sources of inspiration for achieving rational designs in FLIBs.^[^ ^70^ ^]^ The utilization of bio‐inspired structures is an effective solution for overcoming these limitations. Additionally, bionics is another discipline that can contribute to FLIB design. Thus, bio‐inspired structures must exhibit these characteristics.

The diverse hierarchical architectures found in natural materials have evolved through natural selection to adapt to various environments.^[^ ^70^, ^413^ ^]^ These structures can provide valuable guidance for overcoming limitations in materials and engineering techniques. The architectural design at the device level is a more favorable way to release the strain in metal layers.^[^ ^182^ ^]^ Herein, a segmented deformation design of FLIB is manufactured inspired by kirigami. The stretchable FLIBs are also produced based on the idea of kirigami, namely, a combination of folding and cutting. The FLIBs based on kirigami patterns can achieve great stretchability (over 150%).^[^ ^181^ ^]^ Based on the bio‐inspired design, FLIB elliptical deformation of the real state can be converted into the circular strain of the ideal configuration.^[^ ^414^ ^]^ It can maintain > 95% capacity after > 20 000 bending deformations over 30 cycles. And the origami‐inspired FLIBs are manufactured by coating electrodes onto paper current collectors using a slurry, followed by packaging in standard materials and folding using the Miura pattern.^[^ ^415^, ^416^ ^]^ The design of FLIBs with outstanding mechanical characteristics and functionalities is based on the fusion of origami art, materials science, and functional energy storage devices.

**Figure 12:**
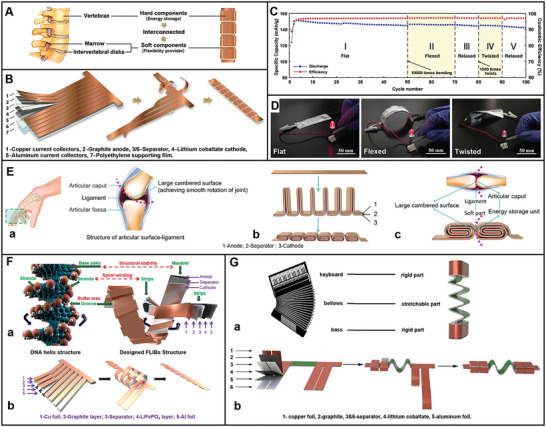
Various types of bio‐inspired designs for FLIBs. A) The schematic illustration of the structure and B) the fabrication process of the spine‐inspired FLIB. C) Charge–discharge cycling test in different configurations at 0.2C. D) The applications to charge light­emitting diode.^[^ ^71^ ^]^ Copyright 2018, Advanced Materials. E) Human joint‐inspired FLIB. a) The structure of an articular surface‐ligament owns the ligament connection and the cambered articular caput surface to achieve flexibility. b) The schematic of the structure and fabrication process of joint‐inspired FLIB. c) The bone corresponds to the thicker electrode stack, the ligament corresponds to the unwound part, and the joint possesses the larger cambered surface.^[^ ^417^ ^]^ Copyright 2021, Energy & Environmental Science. F) DNA helix‐inspired FLIB. a) DNA helix structure and bio‐inspired LIB design. b) During the spiral FLIB fabrication process, the multilayer electrode is first cut into the designed shape, and the extended strips are wound around the mandrel to form the energy stack.^[^ ^72^ ^]^ Copyright 2022, Nano Letters. G) Accordion‐inspired FLIB. a) Accordion and bio‐inspired FLIB design. b) The fabrication process of accordion‐like FLIB.^[^ ^73^ ^]^ Copyright 2019, Energy Storage Materials.

The bio‐inspired design can effectively avoid the plastic deformation of metal current collectors compared with conventional FLIBs.^[^ ^417^ ^]^ **Figure** **12**A,B illustrate a facile and scalable method to fabricating spine‐inspired FLIBs. Material and structural development play significant roles in state‐of‐the‐art FLIBs, involving all components inside.^[^ ^71^ ^]^ A thick, rigid segment for energy storage, achieved through winding the electrodes, is similar to the vertebrae of animals. At the same time, a thin, unwound, flexible part serves as connective tissue, similar to the marrow, interconnecting all stacks inspired by vertebrae. The energy density of bio‐inspired FLIB will be over 85% of that in conventional packing since the volume of the rigid electrode part is larger than that of the flexible interconnection. At a current density of 28 mA g^−1^, FLIBs equipped with LCO, and graphite deform from a flat to a flexed and twisted state during cycling. Despite this, the discharge capacity maintains over 94.3% after 100 cycles with a stable Coulombic efficiency exceeding 99.9% (Figure 12C).^[^ ^71^ ^]^ Figure 12D illustrates that a fully charged FLIB is used to power a light­emitting diode light, which further demonstrates its practical applications.

Further, many FLIB designs achieve extraordinary flexibility, power density, and energy density inspired by biological features. The other bio‐inspired FLIBs have been exploited based on the human joint,^[^ ^417^ ^]^ DNA helix,^[^ ^72^ ^]^ and accordion.^[^ ^73^ ^]^ Drawing inspiration from a human joint's articular surface–ligament structure, FLIBs are engineered for high energy density, multiple deformability, and exceptional durability (Figure 12E).^[^ ^417^ ^]^ In this design, the thick energy storage unit features reserved cambered surfaces at both ends, effectively buffering local stress within interconnected components. Moreover, the shape of the thick stack can be changed using various winding methods, providing FLIBs with ample deformability. In the other design, a bio‐inspired FLIB can realize spiral deformation based on DNA helix‐inspired structure (Figure 12F).^[^ ^72^ ^]^ The FLIB is mainly composed of some grooves for stress buffers and multiple thick energy stacks for energy storage. Such FLIB possesses less than 3% capacity degradation even after ≈31 000 times dynamic mechanical loadings.

In additional, the accordion‐inspired stretchable FLIB, a rigid energy storage unit, is connected by wrinkled and stretchable components (Figure 12G).^[^ ^73^ ^]^ The accordion‐inspired design is a new approach to decoupling mechanical stretching from energy storage, ensuring that stretching exerts minimal stress on electrode particles. Its tape/metal/tape sandwich structure reduces the maximum stress on the Al foil from 31.2 MPa to 17.1 MPa. Additionally, the protective tape provides mechanical support, preventing thin current collectors from breaking during stretching. The bio‐inspired structures significantly enhance the structural stability. In the meantime, a design of this kind can achieve a stretchability of 29% while retaining 77% of the volumetric energy density of conventional packing, as the volume of rigid segments is greater than that of the stretchable component. Benefiting from their novel bio‐inspired design, FLIBs possess a superior mechanical durability, high energy density, high power density, and excellent cycling stability.

In conclusion, bio‐inspired FLIBs disruptively develop the field of wearable electronics, which offer extra functional and physical design spaces. Their outstanding flexibility, mechanical durability, and electrochemical performance are highly promising for practical applications in various flexible and wearable electronics. The bio‐inspired design opens up unique opportunities to commercialize of FLIBs, addressing the current gap in achieving FLIB‐specific deformations to meet the varied requirements of future complex device designs. The bio‐inspired electrodes provide a new strategy to design stretchable FLIBs for stretchable devices emerging flexible devices.

## 6 Easy‐to‐Recycle Electrodes

**Figure 13:**
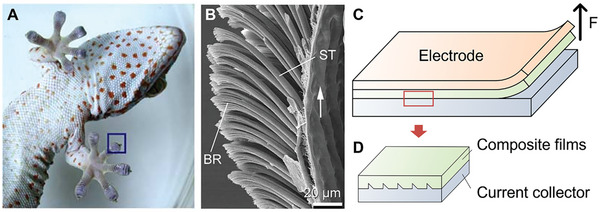
A) The hierarchical adhesive structures of gecko. B) SEM of rows of setae.^[^ ^420^ ^]^ Copyright 2005, Mechanics of Materials. C) Schematic of the 90° peeling tests. D) Enlarged view of red box in (C).

The bio‐inspired electrodes should be considered for their manufacturability and recycling from the early development stages. The recycling of the spent LIBs has significant potential to benefit our society economically and environmentally as well as economizing on raw materials.^[^ ^183^ ^]^ They can be recycled by simple mechanical separation, which is aiming at circular economy, and electrochemical performance.^[^ ^184^ ^]^ Various process chains have been used or are under development to recycle LIBs. The separation of the metal current collector from the composite film of the electrode, however, is a common and serious issue for LIB recycling. To design easy‐to‐recycle electrodes, directional adhesion is desired where interfacial separation along diverse directions requires various fracture energy.^[^ ^74^ ^]^ Highly selective adhesion can be achieved between surfaces by patterning unique structures.^[^ ^418^, ^419^ ^]^ Mimicking the biostructures of nature gives bioinspired smart materials their own switchable adhesion.^[^ ^75^ ^]^ The microstructures on foot hairs of geckos own excellent controllable attachment and detachment capability (**Figure** **13**A,B).^[^ ^420^ ^]^ Based on these structures, a microscale near‐surface architecture is designed on the interface between the current collector and the composite film (Figure 13C,D).^[^ ^76^, ^421^ ^]^ This interface owns controllable and directional adhesion, and thus improved adhesion. It can mitigate the severe volume changes of the composite electrode during lithiation–delithiation cycling. Further, the bio‐inspired composite film of bio‐inspired electrode can be easily pared off from the current collector in a certain direction for recycling.

Consequently, the emerging application of bio‐inspired structural adhesives is significant for the development of bio‐inspired electrodes. The delamination mechanisms and interfacial adhesion of bio‐inspired structures provide scientific footing for the realization of next‐generation easy‐to‐recycle electrodes. It will be of great interest to research more complicated patterns and the correlation between surface patterning inspired by structure in nature without influencing LIB electrochemical performance. This bio‐inspired strategy will be the potential technology to avoid an ever‐growing volume of electrode waste.

## 7 Self‐Extinguishing Electrodes

Thermal runaway is an inevitable safety issue in LIB applications. Intelligent monitoring plays an important role in improving battery safety. Sensors can detect moisture/humidity, gas, and other physical parameters, which can also be further applied in the field of LIB safety monitoring in the future.^[^ ^422^, ^423^ ^]^ Furthermore, it is crucial to pay attention to the thermal hazards of electrode materials, and taking the necessary precautions is of great significance. Self‐extinguishing mechanisms need to build into the structure of bio‐inspired electrodes as a preventive measure to address thermal runaways. The conventional flame‐retardant method of LIB is to modify its components to improve thermal safety.^[^ ^78^ ^]^ Modified components of bio‐inspired electrodes enable them to reduce the likelihood of short circuits and prevent the release of volatile gases.^[^ ^20^ ^]^ The flame‐retardant materials which mix into self‐extinguishing electrodes include epoxy resin,^[^ ^10^ ^]^ triphenyl phosphate (TPP),^[^ ^86^ ^]^ ammonium polyphosphate (APP),^[^ ^49^ ^]^ Al(OH)_3_,^[^ ^82^ ^]^ AlOOH,^[^ ^81^ ^]^ and 9,10‐dihydro‐9‐oxa‐10‐phosphaphenanthrene 10‐oxide (DOPO).^[^ ^83^ ^]^ During the combustion reaction, the flame‐retardant materials emit non‐combustible gas to reduce the oxygen concentration and put out the flame.^[^ ^424^ ^]^ Herein, bio‐inspired electrodes can address the above issues by self‐extinguishing binder,^[^ ^10^ ^]^ microcapsules,^[^ ^49^ ^]^ and current collectors.^[^ ^86^ ^]^ The following sections will discuss these innovations in detail.

### 7.1 Fire‐Extinguishing Binders

**Figure 14:**
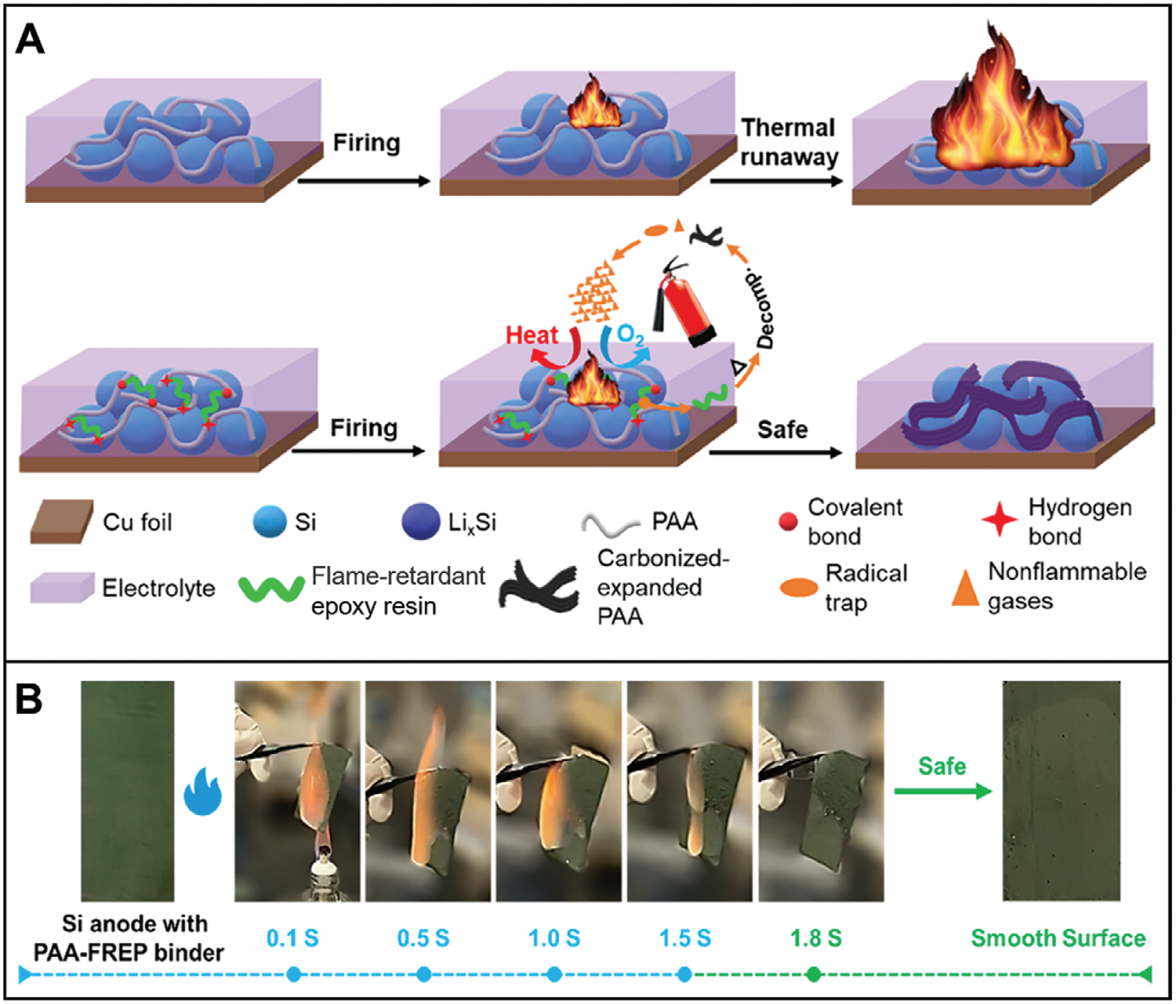
A) Safety comparison of two anodes containing PAA and PAA–flame‐retardant epoxy resin. B) Si anode containing PAA–flame‐retardant epoxy resin binder after immersion in an electrolyte.^[^ ^10^ ^]^ Copyright 2020, ACS Applied Materials & Interfaces.

As previously stated, the bio‐inspired electrodes prepared with a mixture binder of ethylenediaminetetraacetic acid and PAA possess long‐term cycling stability and excellent thermal safety.^[^ ^425^ ^]^ And the specific properties of emerging binders can ensure the service of bio‐inspired electrodes in a wide temperature range (−15 – 60 °C).^[^ ^360^ ^]^ Li−S batteries containing PA binder exhibit a remarkable cycle performance and hence possess flame‐retardant performance.^[^ ^80^ ^]^ To address the thermal runaway of LIBs, the fire‐extinguishing binder can be a preventative measure. For example, a fire‐extinguishing binder has been developed by cross‐linking PAA with a flame‐retardant epoxy resin containing nitrogen and phosphorus elements (**Figure** **14**A).^[^ ^10^ ^]^ This innovation is particularly crucial because thermal runaway and subsequent combustion of LIBs often originate from the anode.^[^ ^186^ ^]^ The synthesized fire‐extinguishing binder, along with the prepared Si anode, effectively suppresses flames through radical trapping, forming a protective layer, and releasing nonflammable gas (Figure 14B). Thus, this fire‐extinguishing binder can enhance the thermal safety of LIBs. Additionally, the cross‐linking between the flame‐retardant epoxy resin and PAA and the epoxy‐functional group can greatly enhance the mechanical properties and cycling stability of the fire‐extinguishing anode. More and more kinds of binders need to be further explored for future usage. This design strategy for a unique multifunction binder could be further extended to other LIB components to possess excellent thermal safety.

### 7.2 Fire‐Extinguishing Microcapsules

**Figure 15:**
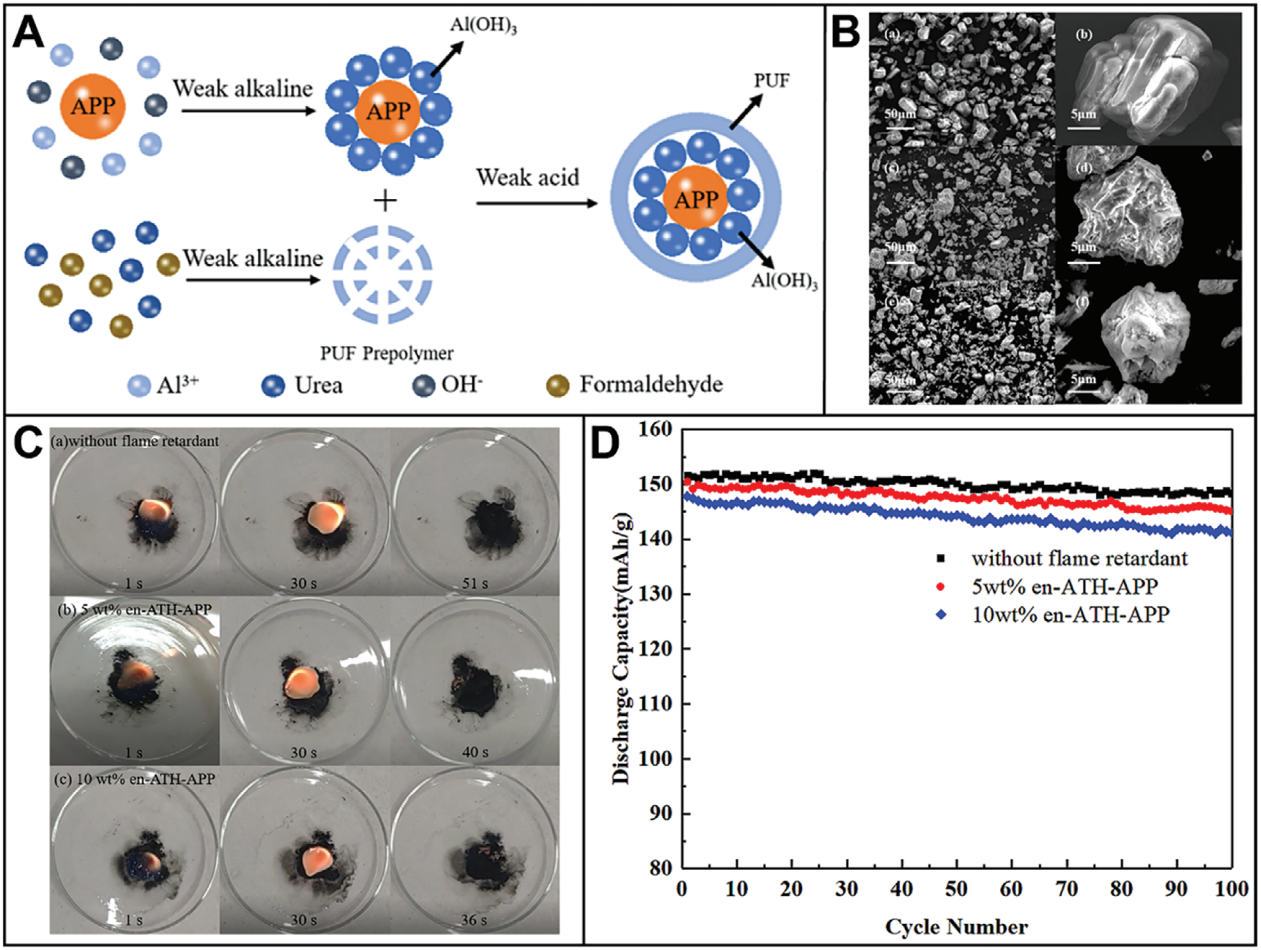
A) Schematic illustration of the Al(OH)_3_–APP encapsulated by PUF to obtain en‐Al(OH)_3_–APP. B) SEM images of a,b) APP, c,d) Al(OH)_3_–APP, and e,f) en‐Al(OH)_3_–APP. C) Combustion process images of a) 0.464, b) 0.467, and c) 0.470 g mixture. D) Cycling performance of LIBs with and without en‐Al(OH)_3_–APP.^[^ ^49^ ^]^ Copyright 2021, ACS Omega.

Outstanding flame‐retardant properties can be achieved via encapsulated boehmites, especially the smaller powder, without significantly sacrificing the electrochemical performances of the cathodes.^[^ ^81^ ^]^ Unlike conventional adding fire‐extinguishing microcapsules in electrolytes,^[^ ^426^, ^427^ ^]^ a microcapsule with flame retardants based on APP and Al(OH)_3_ has been synthesized for application in bio‐inspired electrodes (**Figure** **15**A).^[^ ^49^ ^]^ Al(OH)_3_–APP is prepared by coating the Al(OH)_3_ layer on the surface of the core APP. Next, Al(OH)_3_–APP is encapsulated by PUF to obtain en‐Al(OH)_3_–APP (Figure 15B). Thanks to the synergistic effect between APP and Al(OH)_3_,^[^ ^428^ ^]^ the en‐Al(OH)_3_–APP composite exhibits superior flame‐retardant properties compared to pure APP (Figure 15C). Moreover, the flame‐retardant efficiency of en‐Al(OH)_3_–APP is comparable to that of Al(OH)_3_–APP, indicating that PUF has minimal impact on flame retardancy. Additionally, en‐Al(OH)_3_ maintains excellent retardant properties compared to Al(OH)_3_ alone.^[^ ^82^, ^429^ ^]^ Incorporating en‐Al(OH)_3_–APP into LiFePO_4_ cathodes results in a slight decrease in discharge capacity for such LIBs (Figure 15D). Nevertheless, these cathodes demonstrate superior electrochemical compatibility for LIBs compared to those with pure APP or Al(OH)_3_–APP.

Moreover, various boehmite‐based flame retardants, including AlOOH‐S (0.35 µm), AlOOH‐L (0.95 µm), and their microcapsules (en‐AlOOH‐L and en‐AlOOH‐S) with a chemically stable PUF shell, can be combined into LiFePO_4_ cathodes. Smaller‐sized powders tend to form denser barriers against further decomposition of combustible materials, leading to significantly enhanced fire‐extinguishing efficiency compared to larger ones.^[^ ^81^ ^]^ When not microencapsulated, the inorganic AlOOH and Al(OH)_3_ are hydrophilic, and their addition has no detrimental effect on the dispersion of cathode materials.^[^ ^82^ ^]^ However, en‐AlOOH exhibits slightly lower retardancy compared to AlOOH. Moreover, flame retardants such as TPP and DOPO can be encapsulated within chemically stable PUF, and blended into LiFePO_4_ cathodes. The PUF shell has an approximate thickness of ≈75 nm, with the volume ratio to the microcapsules of en‐TPP and en‐DOPO ranging from 3% to 7%. Despite the relatively low volume percentage of the PUF shell, the fire‐extinguishing microcapsules retain excellent flame retardancy. The fire‐extinguishing efficiencies of en‐TPP and en‐DOPO as cathode additives are 32% and 37%, respectively. Compared to cathodes containing TPP and DOPO, those incorporating en‐TPP and en‐DOPO demonstrate higher capacities and significantly lower impedance.^[^ ^83^ ^]^ Because the organic TPP is hydrophobic and its addition can disrupt to the dispersion of cathode materials, en‐TPP exhibits only half the retardancy of TPP.^[^ ^82^ ^]^ Encapsulation also improves the wetting and dispersion of the initially hydrophobic TPP in the water‐based cathode slurry. Despite the fact that DOPO is inherently hydrophilic, encapsulation nevertheless increased its ability to disperse in the cathode slurry.

In summary, mixing fire‐extinguishing microcapsule into the cathode is a new avenue to improve flame retardancy without a significant electrochemical sacrifice of the cathode. The PUF shell is the crucial coating material used in fire‐extinguishing microcapsules. With the flame retardants microencapsulated, their additions improve the rheology of cathode slurries. With the smaller microencapsulated retardants used, the dispersion of cathode materials is better, and thus the contact between conductive agent and active powder becomes better. Moreover, the electrochemistry is less likely to degrade with an increase in the amount of microencapsulated flame retardants added than it will be with the retardants used in their original form. Encapsulation improves microencapsulated flame retardant decentrality and can be advantageous to slurry casting and electrochemical performance. This newly designed strategy for the bio‐inspired cathode with fire‐extinguishing microcapsules results in LIB possessing a lower impedance and higher rate capability.

### 7.3 Fire‐Extinguishing Current Collectors

The thermal safety issue is a critical challenge in realizing high‐energy LIBs.^[^ ^86^ ^]^ Since metal foil current collectors possessing high density are the essential component of LIBs yet do not contribute to rate capability.^[^ ^84^ ^]^ Furthermore, the current collector is used to collect the electrons from the electrode and transport them to an external circuit.^[^ ^430^ ^]^ Besides, most commercial current collectors are made of metal foil which is quite expensive.^[^ ^431^ ^]^ Herein, the rational design of current collectors can achieve exceptional electrochemical performance,^[^ ^432^ ^]^ decrease the maximum temperature of thermal runaway, and enhance the thermal safety of LIBs.^[^ ^188^, ^433^ ^]^ Thus, the newly fire‐extinguishing lightweight polymer‐based current collector possesses specific capabilities to enhance the energy density and address the thermal safety issue of LIB.^[^ ^84^ ^]^ The application of up‐to‐date current collectors with specific structure and composition can be effective to tackle these shortcomings.

**Figure 16:**
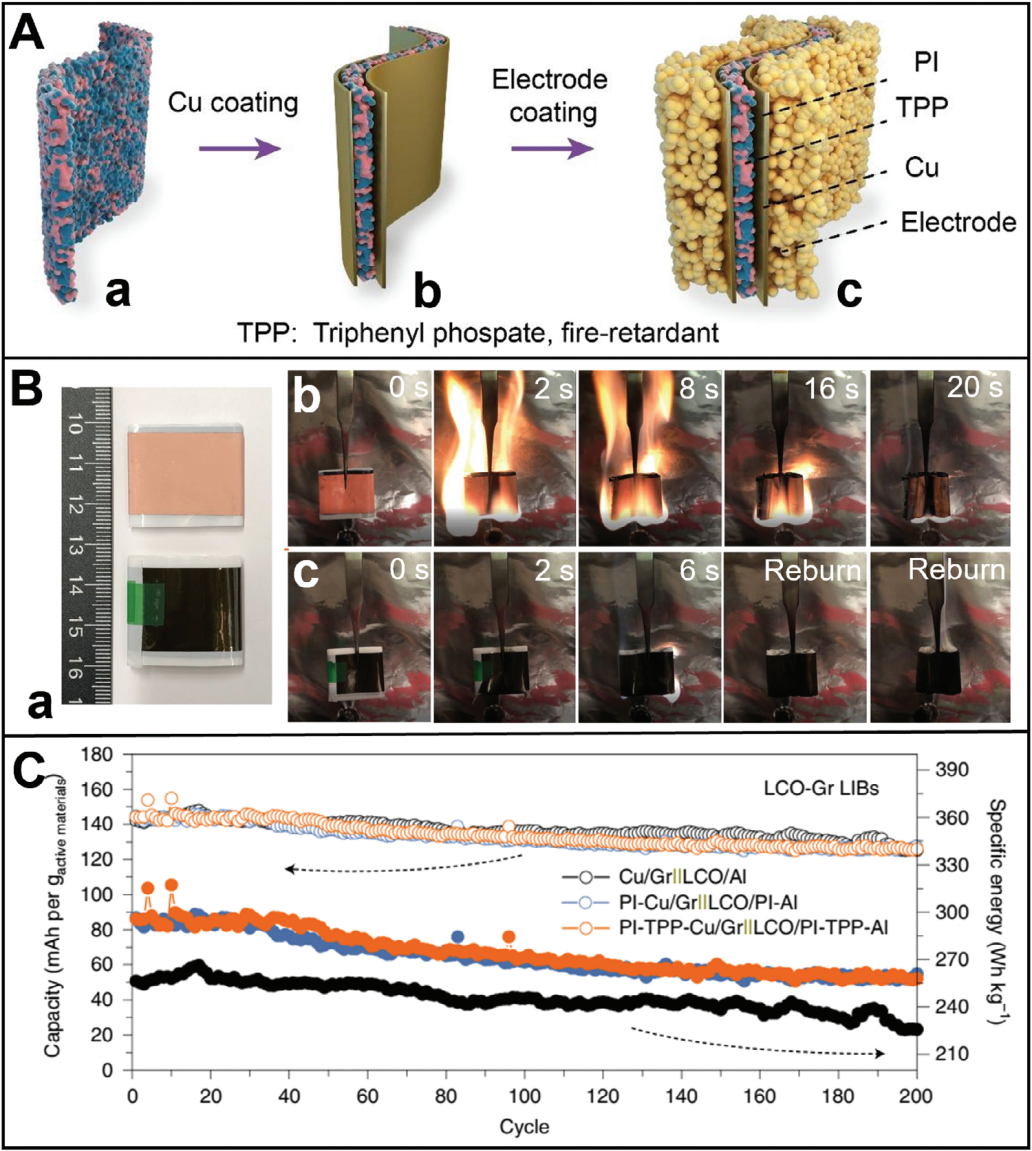
A) The overall synthesis schematic of PI–TPP–Cu current collector‐based electrode: a) PI–TPP, b) PI–TPP–Cu current collector, and (c) fire‐extinguishing electrode. B) Flame retardancy test on LIBs. a) Photos of the assembled Cu/Gr||LCO/Al (top) and PI–TPP–Cu/Gr||LCO/PI–TPP–Al (bottom) pouch full cells. Flame retardancy test results for the Cu/Gr||LCO/Al (b) and PI–TPP–Cu/Gr||LCO/PI–TPP–Al c) pouch full cells. C) Galvanostatic cycling of Cu/Gr||LCO/Al (black), PI–Cu/Gr||LCO/PI–Al (blue), and PI–TPP–Cu/Gr||LCO/PI–TPP–Al (orange) full cells at 0.5C.^[^ ^86^ ^]^ Copyright 2020, Nature Energy.

An emerging fire‐extinguishing current collector minimizes the “dead weight” within LIBs and simultaneously enhances thermal safety. **Figure** **16**A illustrates that the ultralight polyimide‐based current collector (9 µm thick, specific mass 1.54 mg cm^−2^) has been developed by sandwiching a polyimide (PI) embedded with triphenyl phosphate (TPP) flame retardant between two super thin Cu layers (≈500 nm).^[^ ^86^ ^]^ Compared to conventional current collectors made of pure Cu foils, which are bulky and heavy, PI–Cu current collectors are significantly lighter. The as‐prepared PI–TPP–Cu current collector is ultralight and possesses efficient flame‐retardant properties by incorporating TPP and subsequently coating the current collector with ultrathin Cu foil layers on both sides (Figure 16B). The electrochemical performances of LIBs containing Gr and LCO based on each current collector are shown in Figure 16C. Compared to LIBs equipped with the thinnest commercial current collectors (6 µm), LIBs assembled with self‐extinguishing current collectors demonstrate a 16 – 26% enhancement in specific energy. Additionally, they promptly self‐extinguish fires under extreme conditions like thermal runaway and short circuits.

In addition, the other high thermal conductivity and ultralight of current collector with a sandwich structure, the Cu@graphene‐like thermal film composite, is prepared by the vacuum evaporation method and derived from polyimide.^[^ ^87^ ^]^ Various materials can be applied to frame current collectors with special functions, and the electrochemical performances and thermal safety of LIBs mostly hinge on the usage of materials and the corresponding 3D structures.^[^ ^85^ ^]^ Compared with current commercial Cu foil, the fire‐extinguishing current collector possesses a higher thermal conductivity of > 550 mm^2^ s^−1^ (vs. 164 mm^2^ s^−1^ of Cu foil) and a lesser areal density of < 7.0 mg cm^−2^ (vs. 17.50 mg cm^−2^ of commercial Cu foil). The emerging current collectors with ingenious designs will balance the Coulombic efficiency and local current density for high‐performance LIBs by combining different materials with specific functions.

In short, the emerging fire‐extinguishing lightweight polymer‐based current collector simultaneously enhances the energy density and thermal safety of LIBs. Therein, flame retardant encapsulated in the current collector can eliminate potential negative effects on the side reactions and electron/ion pathway. The fire‐extinguishing current collectors will be the advantageous competitor of the conventional current collectors for future excellent thermal safety, energy density, and electrochemical performance of LIBs due to their higher thermal conductivity, lighter mass, and rational electrical conductivity.

## 8 Thermoresponsive Switching Electrodes

Designed reversible thermoresponsive switching electrodes are also a considerable strategy to enhance the thermal safety of LIBs.^[^ ^90^ ^]^ Emerging smart materials possess extraordinary properties, which can alter significantly in controllable approaches when they undergo the appropriate stimulation, such as environmental, thermal, electrical, magnetic, and mechanical changes.^[^ ^89^ ^]^ Then the changed manifestations enable a rapid revert to their premier states once the external stimulation vanishes. Thus, the essential to developing bio‐inspired electrodes possessing thermal‐responding capabilities is to select suitable positive temperature coefficient (PTC) materials. PTC needs extraordinary properties, such as high response speed near the Curie temperature, appropriate Curie temperature, high electronic conductivity at normal operating temperatures, and high chemical and electrochemical stability.^[^ ^92^ ^]^ These polymer PTC composites involve high‐density polyethylene, PVDF, PMMA, ethylene vinyl acetate, etc.^[^ ^88^ ^]^

During regular operations of thermoresponsive switching LIBs, the current flows from the current collector, passing through the positive tab to the metallic foil, bottom disk, top disk, PTC, positive terminal contact, and ultimately to the external load.^[^ ^27^ ^]^ PTC in the bio‐inspired electrodes is a kind of protective device whose resistivity increases with temperature.^[^ ^16^ ^]^ The conductive polymer is one of the PTC materials employed for circuit protection in LIBs. And its resistance, which ranges from 1 to 10 milliohms and is suitable for use at room temperature, changes quickly between the operational and non‐operating states.^[^ ^434^ ^]^ The primary capability of thermoresponsive switching electrodes is to protect LIB against high currents due to overcurrent or external short‐circuit and over‐temperature, and thus avoid thermal runaway.

### 8.1 Thermal‐Responding Additive Materials

**Figure 17:**
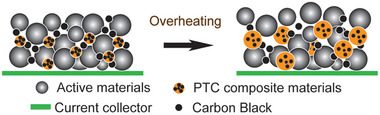
Conceptual illustration of PTC composite cathode.^[^ ^88^ ^]^ Copyright 2019, Small Methods.

As previously stated, directly embedding PTC in cathode materials is a simplest method to achieve a self‐thermoresponsive switching function. Therein, the PTC, as the conductive matrix of the cathode, contains a carbon/polyethylene composite (**Figure** **17**).^[^ ^88^ ^]^ Due to the volume expansion of polyethylene at high temperature, the PTC can quickly change from an electrical conductor to an insulator when inner temperature of LIB increases to the Curie temperature. The peak current within the LIB will be significantly reduced as a result of the materials' conductive network being broken. This will effectively turn off the series of chemical events that lead to thermal runaways.^[^ ^435^, ^436^ ^]^ Upon reaching its glass‐transition temperatures, the polymer component within the PTC material undergoes a significant phase transition, changing into an amorphous state. Concurrently, the distance and conduction channel between implanted carbon black particles also expand owing to the polymer's volume expansion, which results in a nonlinear and rapid rise in PTC resistance. When cooled below its glass‐transition temperatures, the polymer recovers to its crystalline state.^[^ ^27^ ^]^ When the PTC is subjected to temperatures above 100 °C as a result of external short circuits, the conductive polymer also warms up and transforms into a high‐resistance state, which reduces the current demand on the LIB.^[^ ^88^ ^]^ When the PTC‐containing LIB reaches an operational temperature, the polymer transforms back into a conductive state, restoring LIB to its primal operation state.

LIBs containing PTC thermistors are safer than the ones without PTC thermistors, but PTC thermistors can cause higher resistance and produce more thermal losses.^[^ ^27^ ^]^ For instance, one PTC compound consisting of polyethylene and carbon black is a conductive material.^[^ ^436^ ^]^ LIB resistance containing PTC, which corresponds to ohmic resistance increased several times, while the resistance associated with charge transfer increased by more than one order of magnitude at 140 °C. Furthermore, the ethylene vinyl acetate is a based PTC material that embeds into the LiFePO_4_ cathode. Such cathode possesses an obvious current‐limiting effect when the operational temperature is above 90 °C. This temperature is obviously lower than the critical thermal runaway temperature (140 °C).^[^ ^437^ ^]^ Since LIB voltage reached the set voltage early and the short‐circuit current barely flowed due to an increase in impedance, LIB temperature can hardly increase after the short circuit.

Further, the other PTC material, poly(3‐octylpyrrole): poly(styrenesulfonate)/carbon composite, mixed in the LCO cathode can improve the thermal safety.^[^ ^19^ ^]^ The electrochemical performance of LIBs containing PTC is superior to traditional ones at room temperature, but they also have a reliable thermoresponsive switching capacity at higher temperatures. Thus, PTC material possesses a strong tolerance to short‐circuiting, thermal impact, and overcharge. And the acetylene black as a second conductive material added in PTC cathodes can effectively enhance the electrochemical performance. LIBs equipped with PTC cathodes containing a small amount of acetylene black have better discharge characteristics and a longer cycling lifetime than the ones without acetylene black.^[^ ^438^ ^]^ The short‐circuit current of PTC–acetylene black is lower than 1 A at 140 °C, which was almost the same as the current of LIBs containing PTC.^[^ ^435^ ^]^ And the voltage of LIBs containing such PTC–acetylene black decreased precipitously at 135 °C because of a drastic increase in PTC cathode resistivity. Thus, the addition of acetylene black into PTC cathodes improves the electrochemical performance while maintaining LIB safety.

In conclusion, cathode materials containing different compositions can achieve specific capabilities. This thermal‐responding mechanism stems from the PTC effect, wherein the conductive network of cathodes transitions to an insulating state under thermally challenging conditions. LIBs equipped with PTC cathodes possess not only high safety performance from thermal runaway but also excellent electrochemical performance. Moreover, the material choice and fabrication technique of the thermal stability cathode are facile and fully compatible with the current industrial manufacturing process, which makes it convenient for application in practical LIBs. This new strategy of bio‐inspired electrode design is used to construct safer LIBs.

### 8.2 Thermal‐Responding Sandwich Structures

The sandwich structure can be seen everywhere in nature and possesses structural and mechanical stability.^[^ ^416^ ^]^ Advanced sandwich structures can be integrated into the structural design of an effective thermal protection system due to their advantages of low density and high performance.^[^ ^439^ ^]^ Thus, in addition to mixing or in situ coating cathode materials with PTC materials, a novel thermal‐responding cathode framework can be achieved by sandwiching a PTC material between the cathode layer and the Al foil current collector.^[^ ^88^, ^440^ ^]^ In this configuration, PTC materials also serve as a coating layer on the current collector. Coating the current collector with PTC composites enables rapid shutdown of the LIB when it overheats, quickly restoring its functionality when normal operating conditions return.^[^ ^91^ ^]^ These thermal‐responding sandwich structures activate only when the temperature reaches a critical threshold.

**Figure 18:**
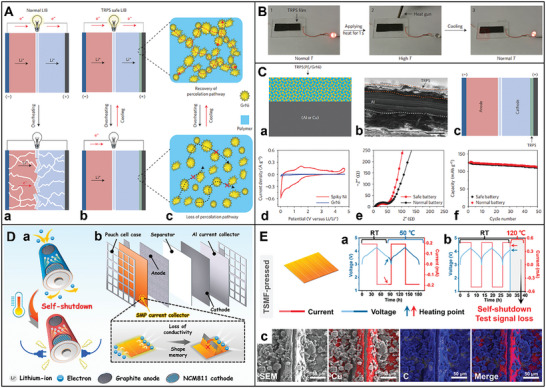
A) Schematic illustration of safe LIB design. a) A normal LIB consists of an anode, a separator, a cathode, and a flammable electrolyte. On abnormal heating, the separator will melt, resulting in internal shorting of LIB. The marked increase in temperature will cause a thermal runaway and permanently damage the LIB structure. b) The thermal‐responding LIB has one or two current collectors coated with a thin thermal‐responding layer. c) The thermal switching mechanism of the thermal‐responding material. B) Demonstration of the thermal switching capability of a thermal‐responding film using an LED connected to the circuit. C) LIB performance. a) Schematic illustration of thermal‐responding film‐coated current collectors. The composite can be coated on various substrates with excellent adhesion. b) SEM image of a polyethylene/GrNi thin film (≈15 µm, 20 vol.% GrNi) coated on an Al foil current collector. c) Schematic illustration of a safe LIB based on the thermal‐responding film coated on Al foil current collector, LCO as cathode, and lithium as an anode. d) Cyclic voltammetry curves of original spiky Ni and GrNi. The GrNi illustrates a much smaller current from undesired side reactions. e) Electrochemical impedance spectra of normal and thermal‐responding LIB. f) Cycling performance of normal and thermal‐responding LIB at 25 °C. Reversible thermoresponsive polymer switching: TRPS.^[^ ^441^ ^]^ Copyright 2016, Nature Energy. D) Design of the shape‐memorized current collectors with micropatterns. a) Self‐shutdown performance of the thermal‐responding LIBs with smart current collectors before thermal runaway. b) The thermal‐responding LIB internal structure and the trigger mechanism of the automatic cut‐out current collector. E) Electrochemical performance of LIBs fabricated using shape‐memorized current collector. a) Cycling profile for thermal‐responding LIBs under room temperature (RT) and 50 °C. b) Cycling profile for thermal‐responding LIBs under room temperature and 120 °C. c) SEM and EDS analysis (carbon, cuprum element, and merge) of the recovered triprism micropattern current collector after 120 °C heating treatment.^[^ ^200^ ^]^ Copyright 2022, Nano Letters.

In practical application, the conventional LIBs will damage at risky temperature (**Figure** **18**A‐a).^[^ ^90^ ^]^ A class of ultrafast, reversible thermal‐responding materials is composed of electrochemically stable graphene‐coated spiky nickel nanoparticles blended within a polymer matrix featuring a high thermal expansion coefficient aimed at enhancing the safety of LIBs (Figure 18A).^[^ ^441^ ^]^ Upon activation, the thermal‐responding film experiences a significant increase in resistance, promptly disabling the LIB under high temperatures or large current flow conditions. The polymer matrix of the thermal‐responding sandwich structure expands, and thus separates the conductive particles, decreasing the value of electrical conductivity by a factor of 10^7^ – 10^8^ on heating.^[^ ^196^ ^]^ Thus, LIBs containing bio‐inspired electrodes with thermal‐responding sandwich structures can be protected without damage. The LIB containing thermal‐responding sandwich structures operates normally at operating temperatures (Figure 18A‐b). Since the quantum tunneling effect enabled by the spiky nanostructure (GrNi), the thermal‐responding film possesses a high electrical conductivity at normal operating temperatures (Figure 18A‐c). The polymer contracts as it cools and reestablishes the original conductive channels.

The thermal‐responding LIB with this thermal‐responding material possesses excellent electrochemical performance at normal operating temperatures and switches off quickly under abnormal conditions (Figure 18B). It can recover normal function without compromising performance during cycling. Figure 18C shows that the polyethylene/GrNi composite exhibits comparable specific capacities of 125 mAh g^−1^ and maintains 95% capacity retention after 50 cycles, highlighting the exceptional cycling stability of the cathode‐side material. This stability is attributed to the utilization of graphene‐coated nano‐spiky nickel particles, which have great electrochemical stability, high temperature sensitivity, and high electrical conductivity at a low particle fraction. Consequently, the sandwich structure with the thermal‐responding material possesses high room‐temperature electrical conductivity, ultrafast thermal switching, a large operating voltage window, excellent mechanical flexibility, and a large decrease of electrical conductivity upon heating. At typical operating temperatures, the bio‐inspired cathode not only performs electrochemically similarly to a traditional cathode, but it also has the necessary thermoresponsive switching ability to turn off the electrode reaction.

Further, the thermal‐responding conductive polymer as a coating layer on the Al foil current collector is used to fabricate a thermoresponsive switching cathode. The novel temperature‐responsive cathode coated an ultrathin layer of poly(3‐octylthiophene) (less than 1 mm) in between the Al foil current collector and LCO layer to constitute a sandwich structured cathode.^[^ ^92^ ^]^ And poly(3‐dodecylthiophene) is a PTC material used as a coating layer of Al foil current collector as well.^[^ ^199^ ^]^ Thermal‐responding sandwich‐structured cathodes demonstrate comparable electrochemical performance to conventional LCO cathodes under standard operating conditions.^[^ ^88^ ^]^ However, when the internal temperature reaches 90 °C, a transformative process occurs, rendering the cathode highly resistive. This effectively interrupts the electrode current, thereby halting the LIB reaction.^[^ ^442^ ^]^ Moreover, a PTC layer can be easily attained by dispersing conductive fillers of multiwall carbon nanotubes within a mixed plastic matrix of PVDF and PMMA polymers.^[^ ^443^ ^]^ The multiwall carbon nanotubes surface and PMMA molecules possess strong H‐bonding interaction. PVDF polymer own the large thermal expansion coefficient. Its resistance coefficient can significantly increase by three orders of magnitude at 110 − 120 °C and suddenly return to the original value at normal operating temperatures even after undergoing multiple thermal cycles. The emerging PTC layer (2–3 µm thick) is a carbon‐coated LiFePO_4_, PVDF, and Super P composite structure. This PTC layer exhibits remarkable structural integrity, serving as a supporting layer between the current collector and the NMC 532 layer.^[^ ^444^ ^]^ The initiation of the PTC effect within the protective layer is prompted by the volume expansion of PVDF at elevated temperatures (above 80 °C). This expansion effectively disrupts electron flow, leading to a significant increase in cathode resistance. Consequently, the bio‐inspired cathode demonstrates a strong yet reversible PTC effect. Thus, the PTC layer effectively prevents thermal runaway without compromising the electrochemical performance of LIBs.

In addition, shape memory polymer is also the other kind of bio‐inspired thermal‐responding material. A newly shape‐memorized current collector has been developed by combining shape memory polymer with micro/nanofabrication technologies, which can successfully prevent thermal runaway under LIB's internal overheating conditions (Figure 18D).^[^ ^200^ ^]^ Unlike conventional current collectors utilizing commercial Cu foils, the shape‐memorized current collector is made from a micropatterned shape memory micron‐sized film with copper deposition. This unique design facilitates unrestricted electron migration and transport through the shape‐memorized current collector when the external circuit is connected under normal conditions (Figure 18E). However, in the event of overheating the collapsed micropattern on the surface transforms, reverting to its original shape to restore functionality. Figure 18E‐c illustrates that the appearance of the pointed shape will puncture the Cu layer that is being covered, creating a ravine that is fractured and obstructing the connection to the thick metal layer. In this scenario, free electron migration on a current collector with shape memory is almost impossible, which results in circuit disconnection. LIB containing a shape‐memorized current collector is able to run normally at temperatures lower than 90 °C, while it quickly achieves self‐shutdown capability before the occurrence of LIB combustion and explosion. The shape‐memorized current collector possesses ideal conductivity at normal operating temperature and turns to be insulative at overheating temperature.

In summary, the thermal‐responding mechanisms of the PTC layer and shape‐memorized current collector provide new insights for designing thermoresponsive switching electrodes. A reliable, fast, reversible strategy for the thermal‐responding sandwich structures on bio‐inspired electrodes possesses outstanding safety during overheating scenarios and maintains electrochemical performance without degradation. This emerging type of PTC electrode can be readily applied to other cathodes to construct safer LIBs owing to its ease of fabrication, cost‐effectiveness, and notably, excellent compatibility with current LIB technology. Moreover, this approach offers a promising pathway for designing reversible thermo‐responsive materials and enhancing the safety of LIBs.

### 8.3 Thermal‐Responding Microspheres on Electrodes

The specific capabilities of autonomic and thermoresponsive switching for bio‐inspired electrodes can be achieved by incorporating thermoresponsive polymer microspheres onto anode layers. When subjected to a significant current, the temperature of the PTC element increases rapidly owing to the the generation of Joule heat within the PTC element.^[^ ^93^ ^]^ At temperatures surpassing its melting point, the PTC experiences a rise in resistivity. Thus, the abnormal and concomitant high resistance of the PTC element prevents current flow.

On the one hand, carbon nanotubes are applied as a coating onto polyethylene microspheres, achieved through straightforward and scalable solvent evaporation method followed by surface treatment and subsequent mixing with carbon nanotubes.^[^ ^445^ ^]^ Polyethylene microspheres are used as a switch‐off additive by coating on the LiFePO_4_ cathode. Melting of the polyethylene at high temperatures results in the formation of an insulating film that impedes the flow of lithium‐ions. By melting the polyethylene microspheres, this type of LIB gains the ability to switch‐off. This approach significantly reduces the need for additive loading, enabling the bio‐inspired cathode to achieve rapid switch‐off within 60 s using only 1 mg of additive. This method can achieve enhanced safety, whereas LIB can hardly recover the charging/discharging capacity once the temperature returns to normal.

**Figure 19:**
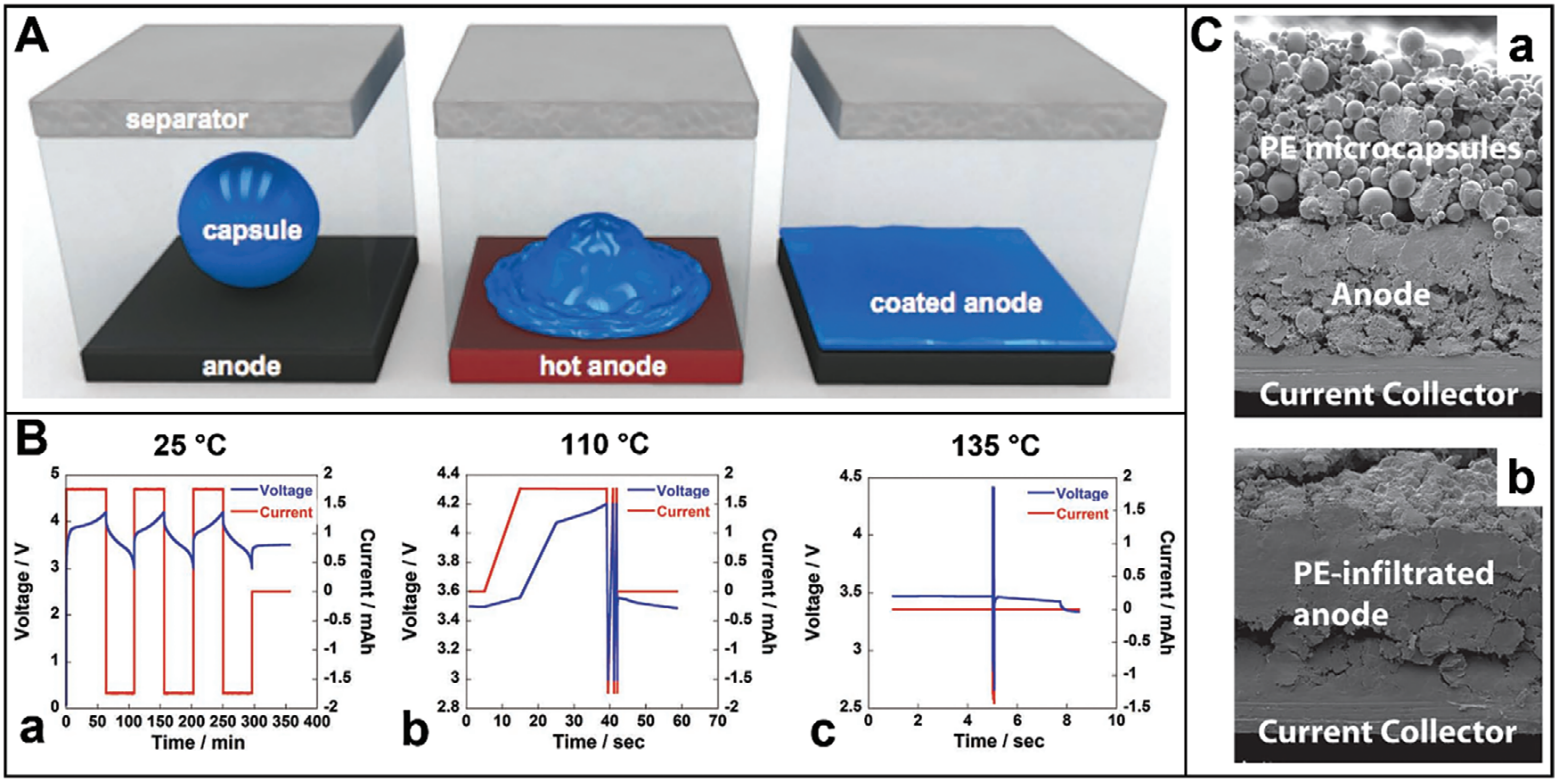
A) Schematic illustration of microsphere‐based switch‐off concept for LIBs. B) Voltage and current profiles for LIBs containing a polyethylene microsphere functionalized anode. a) Room‐temperature cycling profile for a LIB equipped with a commercial separator and polyethylene microspheres (12.7 mg cm^−1^ coverage) on the anode. b) Cycling profile demonstrating switch‐off achieved using polyethylene microspheres on the anode at 110 °C. c) Cycling profile at 135 °C for LIB previously switch‐off at 110 °C. C) SEM images of anode cross sections. a) Anode with polyethylene microspheres before cycling and b) incorporation of anode with polyethylene microspheres after cycling at 110 °C.^[^ ^446^ ^]^ Copyright 2012, Advanced Energy Materials.

On the other hand, the emerging thermal‐responding microspheres on electrodes have been developed. For instance, the microspheres melt and coat the anode with a nonconductive barrier, halting lithium‐ion transport and switching off LIB when the internal LIB environment reaches a critical temperature (**Figure** **19**A).^[^ ^442^, ^446^ ^]^ For polyethylene microspheres coated on the anode layer, the initial capacity of LIB is unaffected by the presence of the polyethylene microspheres. LIB shutdown at 110 °C, and then heated to 135 °C with no further change in the voltage or current profile (Figure 19B). The anode surfaces have undergone autonomic switch‐off by melting, wetting, and resolidification of polyethylene into the anode and polymer film formation at the anode interface (Figure 19C). Thus, LIB is shut down due to microsphere activation.

In short, the thermal‐responding microspheres coat on the anode layers are helpful for thermal‐responding LIBs. The mechanism of the switch‐off is that the microspheres melt film prevents ionic flow, and results in disabling LIB. Thermal‐responding microspheres as a rapid switch‐off additive own the flexibility usage characteristic and hence are applicable in a wide range of LIB chemistries to attain improved thermal safety. Thus, the resistance of the thermal‐responding microspheres significantly rises upon activation, resulting in a sharp fall in the current, which restricts heat generation in bio‐inspired smart LIB.

### 8.4 Thermal‐Responding Skin on Cathode Particles

**Figure 20:**
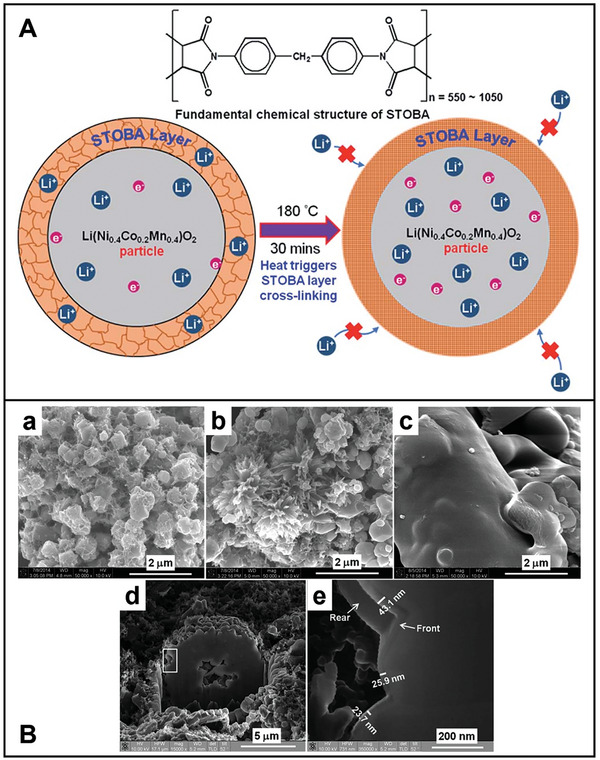
A) Schematic illustration of the change in morphology of the STOBA layer in the STOBA‐coated NMC 442 particle after thermal treatment. B) SEM images of STOBA material a) without and with thermal treatment at b) 150 °C and c) 180 °C, and d) cross‐section of a STOBA‐coated NMC 442 particle. e) The enlarged image of the marked region of d).^[^ ^94^ ^]^ Copyright 2014, RSC Advances.

The polymer coating as a thermal‐responding skin does not impact cycling performance at ambient temperature and a thermal shutdown function at an elevated temperature.^[^ ^447^, ^448^ ^]^ For instance, the morphological change of the self‐terminated hyper‐branched oligomers (STOBA) layer from the porous to nonporous state at thermal runaway temperature.^[^ ^94^ ^]^ STOBA, as a thermal‐responding skin is coated onto Li(Ni_0.4_Mn_0.4_Co_0.2_)O_2_ (NMC 442) particles and subsequently melted to create a dense film at high temperatures (**Figure** **20**). The thermal safety mechanism of the STOBA‐cathode is ascribed to several factors, including the inhibition of gas evolution, passivation of the cathode–electrolyte contact, and blocking of lithium‐ion and electron transfer at the interface.^[^ ^447^ ^]^ The STOBA coating effectively neutralizes the surface activity of the cathode particles, and thus the constitution of CEI under high voltage is restrained. The rate of heat generation caused by electrolyte decomposition and CEI breakdown at the heated charged cathode surface is greatly decreased. Thus, thermal‐responding skin is an effective coating design for suppressing the constitution of by‐products from electrolyte disintegration reactions and stabilizing the cathode structure at high voltage and temperature.

Further, NMC 811 particle coating with poly(3‐octylthiophene) is a “killing two birds with one stone” strategy to concurrently enhance the thermal safety and cycling stability of cathode materials.^[^ ^449^ ^]^ Exposure to hazardous temperatures promptly suppresses activity without significantly compromising capacity. Consequently, the thermal‐responding skin does not allow LIBs to overheat and prevents the occurrence of thermal runaway. At standard operating temperatures, the PTC‐coated NMC 811 cathode exhibits remarkable capacity retention, with a retention rate of 92.3% after 200 cycles at 0.5C.^[^ ^449^ ^]^ It can recover its charging/discharging capabilities while the temperature returns to normal. And NMC 622 particle surfaces coated with oligomers (N,N′‐bismaleimide‐4,4′‐diphenylmethane with 5,5‐dimethylbarbituric acid) significantly improved the cycling stability, rate capability, and thermal stability.^[^ ^104^ ^]^ Such cathode possesses excellent thermal safety in the charged state, a decreased reaction heat flow of 114.0 J g^−1^, and a delayed exothermic reaction temperature of 544.2 K. Furthermore, LCO particle coated a conducting polymer ‘‘skin’’, the poly(3‐decylthiophene), has a PTC transition temperature of 80 – 110 °C.^[^ ^448^ ^]^ This kind of material not only enhances the cycling stability at normal operating temperature but also achieves a thermal switch‐off function at risky temperatures, which provides self‐activating thermal protection for LIBs.

In summary, the thermal‐responding skin coating cathode particle can inhibit the constitution of by‐products from electrolyte disintegration reactions and stabilize the cathode structure at high voltage and temperatures. Since the thermal‐responding skin cathode coating contributes to the inhibition of thermal runaway, both Joule‐heating and heat generation by electrolyte‐oxide interfacial reactions can be reduced during the initial stage of heating up. It plays a significant role in preventing internal short‐circuiting and thermal runaway. In addition, the developed method can enlarge the exploitation of the materials mixed in the emerging bio‐inspired electrodes for building thermostable and safer LIBs.

## 9 Outlook

In the long‐term cycling, innovative LIB technologies such as novel electrode chemistries and 3D architectures are to be pursued to ultimately enable fast charging and widespread electric vehicle adoption. Establish effective feedback loops between the battery management system and LIB sensing to enable built‐in self‐healing mechanisms to be properly triggered by an external stimulus. For instance, different on‐demand self‐healing capabilities will be developed using various chemical and physical methods. Develop supramolecular assemblies relying on hydrogen bonding for reversible crosslinking to repair electrode and interface fracturing while being compatible with the targeted LIB chemistry. Conventional Si‐based anodes and emerging anode materials such as Sn‐ and Ga‐based anodes with self‐healing capabilities will be used in bio‐inspired smart LIBs. Instead of the present commercial PAA binder and carbon black combinations, a multifunction polymeric binder with self‐healing capabilities could potentially be utilized. The wisely engineered bio‐inspired electrodes containing capsules holding organic and inorganic healing agents with various capabilities can be triggered to self‐healing by a magnetic, thermal, or electric stimulus.

The developments of bio‐inspired synthesis and structures can achieve various specific capabilities on bio‐inspired electrodes. Bio‐inspired porous Si particles are a promising candidate for next‐generation LIBs owing to their attribute, including high specific capacity, low lithiation–delithiation potential, low cost, and environmental friendliness. The intricate nature of biomass materials or bio‐inspired structures provides possible strategies for advancing bio‐inspired smart LIBs. Carbon‐based electrode materials can be derived directly from abundant biomass sourses. The synthesis of bio‐inspired materials and integration of smart structures progressively assume a pivotal role in electrochemical energy storage and conversion technologies. Design and manufacture low‐cost bio‐inspired structures on smart electrodes possessing controlled functionalities and porosity for ion diffusion to improve cycling lifetime and electrochemical performance.

The development of FLIBs is critical for the next generation of electronics. Based on bio‐inspired structures, they achieve deformations such as twisting and bending. Thus the break causing their failure to work or even serious safety problems during the practical application will be addressed. Specific capacity, rate capability, and cycling performance must be effectively maintained even after repeated deformation. Bio‐inspired concepts offer valuable insights into the development of structural materials conducive to flexible device applications while also paving the way for innovative bio‐inspired funcionalities ‐in future technologies.

The fire‐extinguishing binder consisted of flame‐retardant epoxy resin and PAA, which could enhance the thermal safety of LIBs. Additionally, incorporating other flame retardants into binders could enable the creation of self‐extinguishing electrodes. Employing encapsulation by PUF could help mitigate the hydrolysis of flame retardants, thus minimizing their adverse impact on the electrochemical performance of the cathodes. The encapsulation process enhances the dispersity of microencapsulated retardant, facilitating better slurry casting and improving the resulting electrochemical performance. Considering attributes such as lighter mass, higher thermal conductivity, and reasonable electrical conductivity, fire‐extinguishing current collectors could emerge as favorable alternatives to commercial current collectors, meeting the requirements for enhanced thermal safety, energy density, and electrochemical performance of future LIBs.

The characteristics of emerging smart materials undergo significant, controllable changes in response to various stimuli, including thermal, electrical, environmental, mechanical, pH variations, and magnetic influences. These altered properties promptly revert to their original states once the external stimulus ceases. PTC thermistors are resettable devices that passively restrict high current surges, effectively preventing overcurrent situations. Integrating PTC materials into bio‐inspired electrodes is a promising solution to enhance thermal responsiveness to high temperatures. When triggered, these electrodes temporarily deactivate LIBs, only to restore conductivity once the hazards subside. However, further research is necessary to minimize adverse effects on electrode conductivity and LIB performance.

Research on bio‐inspired electrodes is gaining momentum as we look ahead. This field offers an interdisciplinary field for researchers from diverse backgrounds to explore innovative concepts, materials, structures, and designs. Progress in high‐performance bio‐inspired electrodes will drive the evolution of LIBs, extending their lifetimes, enhancing safety, and enabling flexibility and wearability, thus enriching our daily lives. While many emerging components have yet to be fully optimized for commercial LIB applications, they hold significant potential in advancing the future usability of LIBs.

## 10 Summary

**Figure 21:**
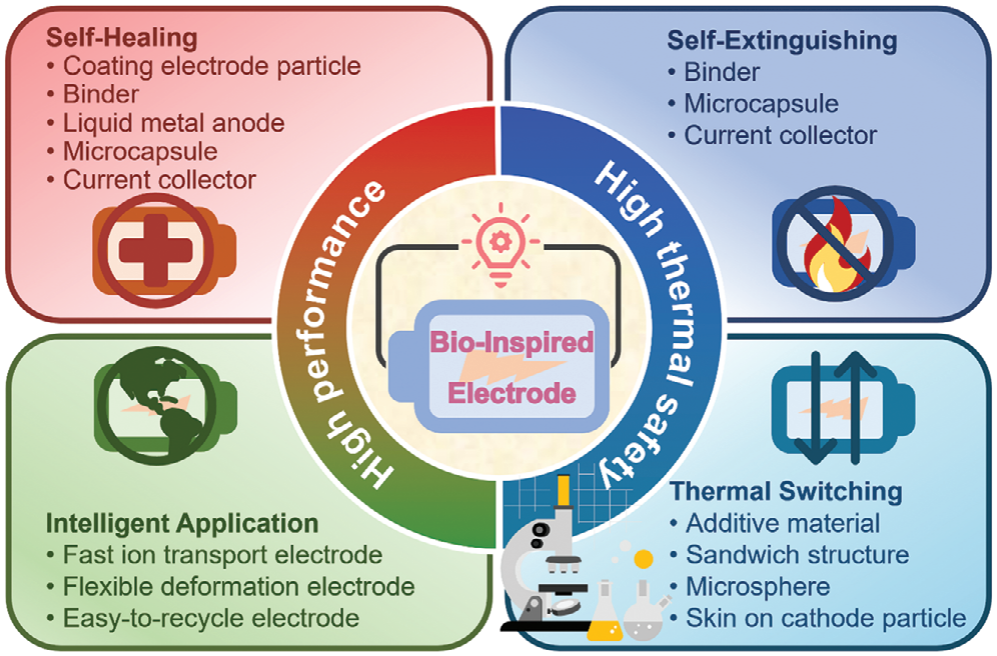
The future development directions of bio‐inspired electrodes for LIBs.

Without a doubt, LIB is the major kind of power source for energy storage devices. The efficient measures of a prolonged lifetime decreased fires caused by thermal runaway, and enhanced electrochemical performance advance its applications. Over the past decades, considerable efforts have been dedicated to enhancing cycling stability, rate capability, and thermal safety by designing various bio‐inspired structured smart electrodes. This review summarizes the latest research endeavors, emphasizing electrode materials, innovative design strategies, additive agents, current collectors, and bio‐inspired electrodes with bio‐inspired functions. The advantages of the bio‐inspired structures as well as the perspective of this research field are also discussed. The smart features of new electrodes include self‐healing, self‐extinguishing, thermoresponsive switching, flexible deformation, and easy‐to‐recycle function, which can be achieved by developing core‐shell structured electrodes (cathode and anode) particles, new binder, current collectors, microcapsules, and bio‐inspired structures (**Figure** **21**).

LIBs equipped with self‐healing electrodes possess longer durability, better electrochemical performance, and higher thermal safety. Developing bio‐inspired electrodes utilizing self‐healing materials is one of the most promising avenues for their practical implementation. These electrodes leverage supramolecular reactions and covalent/free radical re‐bonding to achieve self‐healing through supramolecular interactions or reversible chemical bonds. While theoretically capable of achieving permanent healing, these mechanisms may exhibit relatively lower efficiency in practice. The durability of LIBs can be prolonged by using preventive steps—bio‐inspired electrodes containing self‐healing materials, such as self‐healing binders, current collectors, microcapsules, and LM anodes. The bio‐inspired electrodes owning self‐healing capabilities can maintain the structural integrity during lithiation–delithiation cycling process.

The bio‐inspired electrodes equipped with fire‐extinguishing current collectors and capsules can release flame retardants during thermal runaway, which are effective ways to improve LIB safety. The measures to prevent the occurrence of thermal runaway include adding flame‐retardant additives into a fire‐extinguishing binder, current collector, and capsule. A fire‐extinguishing binder is formulated by cross‐linking PAA with a flame‐retardant epoxy resin containing phosphorus and nitrogen elements. This innovative approach to crafting a multifunctional binder can extended to various other components of LIBs or energy storage devices, thereby enhancing their thermal safety. The fire‐extinguishing lightweight polymer‐based current collector increases the energy density of LIBs and improves their thermal safety. Additionally, the prepared fire‐extinguishing microcapsules effectively enhance the thermal safety of LIBs during the initial stages of thermal runaway while maintaining their electrochemical performance. These microcapsules exhibit exceptional flame retardancy within the electrode mixture without significantly compromising the electrochemical properties of the bio‐inspired electrodes.

The thermoresponsive switching electrodes containing thermal‐responding additive materials, thermal‐responding sandwich structure and thermal‐responding microspheres, and thermal‐responding skin, can rapidly switch LIB off when subjected to overheating and quickly resume functionality once normal operating conditions are restored. Coating the cathode with additive materials effectively prevents the formation of byproducts from electrolyte decomposition reactions and stabilizes the cathode structure in LIBs under high charging voltage and temperature conditions. By addressing the critical issues of poor cycling stability and inadequate thermal safety of electrode materials, this approach presents a new avenue for advancing the commercial application of bio‐inspired electrodes.

Materials and structures in nature have evolved to the most efficient forms and adapted to various environmental conditions over thousands of years. The requirement for bio‐inspired structures is designed to enable LIBs to possess high power density and cycling stability. Herein, bio‐inspired concepts will be introduced in bio‐inspired electrodes designed to enhance the electrochemical performance of LIB. Bio‐inspired concepts can seamlessly integrate into fabricating electrode materials for energy storage and conversion. Furthermore, careful attention should be given to the synergy between electrode structures and materials. When selecting bioresources, consideration should also be given to the coherence of the final products because they may vary in nature. By mimicking natural architectures and biological processes, the unique structures of active materials required for ensuring high cycling performance can be rationally designed and prepared. Bio‐inspired hierarchically nanostructured electrodes with void spaces accommodate volume changes associated with lithiation and delithiation. It is anticipated that this review will inspire further advancements in LIB lifetime and thermal safety, especially for emerging bio‐inspired electrodes possessing high electrochemical performance. Consequently, bio‐inspired electrodes possess spatiotemporal management of self‐healing, fire‐extinguishing, thermoresponsive switching, recycling, and flexibility, which have the best application prospects for next‐generation high‐energy‐density LIBs in the future.

## Conflict of Interest

The authors declare no conflict of interest.
